# Decoupling Dissolution from Water Oxidation at Single-Faceted High-Entropy Perovskite Electrodes

**DOI:** 10.1021/jacs.6c09065

**Published:** 2026-10-21

**Authors:** Subhajit Bhunia, Esteban D. Gadea, Alessandro R. Mazza, Mohana V. Kante, Gangli Wang, Sheng Dai, Thomas Z. Ward, Valeria Molinero, Henry S. White

**Affiliations:** † Department of Chemistry, 7060 University of Utah, 315 South 1400 East, Salt Lake City, Utah 84112, United States; ‡ Center for Nanophase Materials Sciences, 6146 Oak Ridge National Laboratory, Oak Ridge, Tennessee 37830, United States; § Chemical Sciences Division, Oak Ridge National Laboratory, Oak Ridge, Tennessee 37831, United States; ∥ Department of Chemistry, 1373 Georgia State University, Atlanta, Georgia 30302, United States; ⊥ Materials Science and Technology Division, 5112 Los Alamos National Laboratory, Los Alamos, New Mexico 87545, United States

## Abstract

High-entropy oxides (HEOs) are promising anode materials for the oxygen evolution reaction (OER) due to their wide compositional space and electronic flexibility. However, their electrochemical dissolution and OER remain concurrent, preventing decoupled analysis of dissolution kinetics and stability. Here, we present a combined atomic force microscopy (AFM) and scanning electrochemical cell microscopy (SECCM) strategy to decouple lattice dissolution from water oxidation under well-defined reaction conditions. Structurally analogous, atomically flat, single-faceted (001) *AB*O_3_ perovskite electrodes, La_1–*x*_Sr_*x*_(Cr_0.2_Mn_0.2_Fe_0.2_Co_0.2_Ni_0.2_)­O_3_ with *x* = 0 and 0.3, serve as model systems to probe dissolution and OER kinetics in acid. Pipet-probed localized cyclic voltammetry and chronoamperometry reveal that the measured current contains contributions from both OER and lattice dissolution, the latter producing nanoscale dissolution pits beneath the SECCM droplet. By correlating pit-growth kinetics with total OER charge as a function of pulse duration (τ), overpotential (η), and pH, we quantitatively separate dissolution from OER. The proportional scaling between dissolution charge and total charge indicates that OER and dissolution proceed within a common kinetic regime, sharing a common intermediate. A rate-law-based analysis yields the standard dissolution rate constant (*k*_d_^0^ cm/s), transfer coefficient (α), and proton reaction order (*b*) for the dissolution of the 001 facet of both HEO-P. Our results unravel how aliovalent *A*-site substitution (30% Sr^2+^) alters dissolution kinetics, charge partitioning between dissolution and OER, and thickness-dependent stability, thereby enabling quantitative disentangling of the intrinsic activity and stability of HEO electrodes.

## Graphical abstract

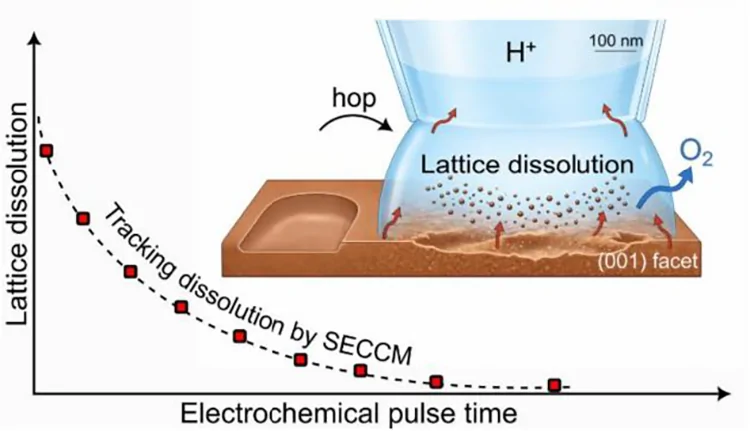

## Introduction

Proton exchange membrane water electrolyzers (PEMWEs) have advanced to a commercial level and offer a promising route toward industrially relevant hydrogen production.^1^ A persistent obstacle, however, is the lack of acid-stable anodes; operation at low pH and high anodic potentials imposes a fundamental bottleneck between activity and stability under strongly corrosive conditions.^2,3^

In recent years, high-entropy oxides (HEOs) have gained significant attention for the oxygen evolution reaction (OER) because of their vast compositional space, multiple active sites, and tunable electronic structures.^4–6^ In high-entropy configuration, several metal cations coexist in proximity offers a mean field electronic environment that optimizes binding of *OH, *O, and *OOH species, thereby lowering the overall overpotential.^7,8^

Despite these advantages, the practical implementation of HEO-based anodes in PEMWEs remains challenging because of their limited electrochemical stability at low pH and at industrially relevant current densities, even though they exhibit excellent stability at high pH.^9^ Under acidic conditions, dissolution occurs concurrently with water oxidation, leading to ambiguous evaluations of intrinsic activity and stability.^10^ Moreover, oxygen bubble accumulation and complex dissolution–redeposition events create challenges to delineate dissolution from oxygen evolution events.^11–13^

Most stability assessments for acidic OER have been developed for precious-metal oxides such as IrO*_x_* and RuO*_x_*.^14–16^ Fewer quantitative studies address dissolution of nonprecious oxides under low-pH, high-potential operation, despite emerging evidence that selected single- and multinary oxides can exhibit appreciable stability in acid under certain synthetic and device-relevant conditions.^17–25^ Recent studies have increasingly focused on developing experimental strategies to disentangle electrochemical dissolution from oxygen evolution in both single-oxide and complex multicomponent catalyst systems.^26–28^ Quantitatively resolving these processes under identical reaction conditions remains challenging due to heterogeneity in material characteristics.

To address the gap, we employed scanning electrochemical cell microscopy (SECCM), which has emerged as a powerful, high-throughput, and versatile approach for probing local structure–property relationships.^29,30^ SECCM can be used to study the kinetics of electrochemical processes occurring on homogeneous catalyst films. The fluidic meniscus formed at the tip of a nanopipet acts as a picolitre electrochemical cell, enabling measurements with excellent spatial resolution. Each scan operates as a droplet-confined electrochemical system, providing precise control over overpotential (η) and reaction time (τ). Specifically, for O_2_-evolving reactions, SECCM offers an inherent advantage by creating a three-phase boundary around the droplet.^31–33^ The short diffusion length defined by the droplet radius facilitates rapid oxygen escape through the air–liquid interface, minimizing bubble accumulation. Simultaneously, the conical pipet geometry promotes efficient ion diffusion from the electrode directly to the pipet, reducing redeposition of dissolved species.

Using SECCM, the dissolution process can be confined to a small catalyst layer region of various predefined thickness, and its kinetics within this region can be systematically examined by adjusting η, τ, and pH. Building on these capabilities, we coupled SECCM with AFM to probe catalyst dissolution during OER. By colocating the electrochemical reaction site with AFM spectroscopy, changes on the electrode surface can be imaged as a function of τ, η, and pH.

We investigate two structurally analogous epitaxially (001)*AB*O_3_-type perovskite high-entropy oxide (HEO-P) thin films: La­(Cr_0.2_Mn_0.2_Fe_0.2_Co_0.2_Ni_0.2_)­O_3_ (La-HEO-P) and its 30% Sr-substituted (at the *A*-site) counterpart, La_0.7_Sr_0.3_(Cr_0.2_Mn_0.2_Fe_0.2_Co_0.2_Ni_0.2_)­O_3_ (LaSr-HEO-P), as a model system. The La-HEO-P electrocatalyst exhibits superior performance relative to its single metal oxide components in alkaline OER. This motivated us to choose this catalytic material and the corresponding Sr-substituted variant.^5^ Substitution of Sr^2+^ for La^3+^ provides controlled electronic tuning of the perovskite lattice while preserving crystal structure and surface orientation. These two electronically distinct but structurally identical systems provide a basis to isolate the doping-induced effects on electrochemical behavior without introducing additional structural complexity and offer a stringent platform to examine how hole doping influences HEO-P electrode dissolution and OER activity at low pH.

By combining SECCM and AFM, we quantify the spatiotemporal pit growth evolution and fit the results to a rate-law-based kinetic expression. This analysis yields the standard rate of dissolution (*k*_*d*_^0^), proton reaction order (*b*), and a transfer coefficient (α) for dissolution, and enables direct comparison between La-HEO-P and LaSr-HEO-P.

Finally, we correlate these results with the coulomb ratio between lattice dissolution and OER as a function of η and pH for both HEO-P. Together, these measurements support a mechanism to distinguish how aliovalent doping in HEO-P influences the kinetic branching between OER and dissolution.

## Result and Discussion

We chose two high entropy *AB*O_3_ perovskites, La-HEO-P and LaSr-HEO-P (Figure 1a), where *A*-site composition has induced hole doping on *B*-site sublattice. For each composition, both thick and thin films were prepared: 89– and 29–nm thick La-HEO-P, and 100– and 29–nm thick LaSr-HEO-P (Figure S1). Epitaxial (001)-oriented La-HEO-P and LaSr-HEO-P films were synthesized on Nb-doped conducting SrTiO_3_ (Nb: STO) substrates by pulsed-laser deposition to achieve atomically smooth, single-crystalline electrodes for SECCM measurements. Each film was structurally characterized by high-resolution X-ray diffraction (HRXRD, Figure S2), X-ray reflectometry (XRR, Figure S3), and atomic force microscopy (AFM, Figure S4) to determine the crystal orientation, thicknesses, and surface morphology (Figure S4). HRXRD and XRR confirmed epitaxial growth of the (001)-oriented perovskite films with well-defined thicknesses, while AFM confirmed subnanometer surface roughness (<1 nm). Quantitative EDS mapping and XPS were performed at multiple locations across the HEO-P electrodes, confirming a homogeneous elemental distribution with negligible variation in the relative elemental composition, demonstrating the compositional uniformity of the high-entropy oxide films (Figures S5–S12). The single-crystal, atomically flat, and compositionally uniform catalyst films ensure that the SECCM measurements remain equivalent everywhere on the surface.

**Figure 1:**
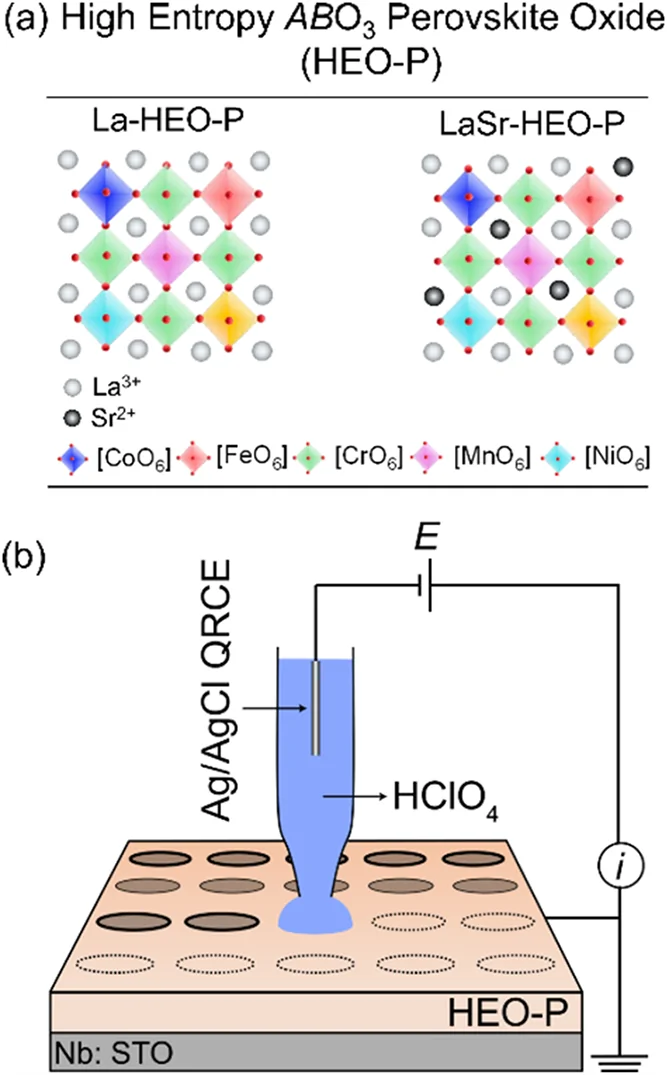
(a) High entropy perovskite (HEO-P) catalyst composition; (b) Schematic for SECCM scan hopping experiment on HEO-P 001 faceted electrode.

Figure 1b shows a schematic of the SECCM configuration. Experiments are conducted using nanopipets with a diameter (*d*_pipet_) of ∼750 to 800 nm, filled with 100 mM NaClO_4_:HClO_4_ electrolyte adjusted to different pH, and equipped with an Ag/AgCl quasi-reference counter electrode (QRCE). During each landing event on the HEO-P surface, the meniscus formed a droplet-confined electrochemical cell on the perovskite surface, enabling localized cyclic voltammetry (CV) and chronoamperometry *i–t* measurement under a well-defined potential bias between the HEO-P (working electrode) and QRCE (Figure S13).

Localized cyclic voltammograms (CVs) were then recorded by scanning between 1.1 and 1.8 V vs RHE using SECCM, corresponding to the water oxidation potential window at pH 1. From the first cycle, both electrodes behave as OER-active anodes, showing a sharp anodic rise, with onset potentials of ∼1.64 V for La-HEO-P-89 nm and ∼1.69 V for LaSr-HEO-P-29 nm (determined at a current density of ∼2 mA/cm^2^ where current was normalized by pipet diameter).

Upon repeated cycling, both materials display a characteristic evolution of current of gradual activation followed by deactivation (Figure 2a,b). Such temporal evolution suggests possible surface changes under OER bias and indicates that the observed current is intrinsically coupled to concurrent surface changes. To quantify cycle-by-cycle evolution, the current at ∼ 1.74 V was plotted as a function of cycle number for both electrodes (Figure 2c). LaSr-HEO-P-29 nm displays a sharper activation–deactivation transition reaching a maximal current of ∼0.6 nA by the sixth cycle, whereas La-HEO-P-89 nm attains a similar value by the eighth cycle. The overlay of CVs at their respective maximum currents reveals their similar *i–E* response. Both materials achieve a current density of ∼40 mA cm^–2^ (normalized by geometric area of pipet) at potentials of ∼ 1.70 V for La-HEO-P-89 nm and 1.71 V for LaSr-HEO-P-29 nm (Figure 2d).

**Figure 2:**
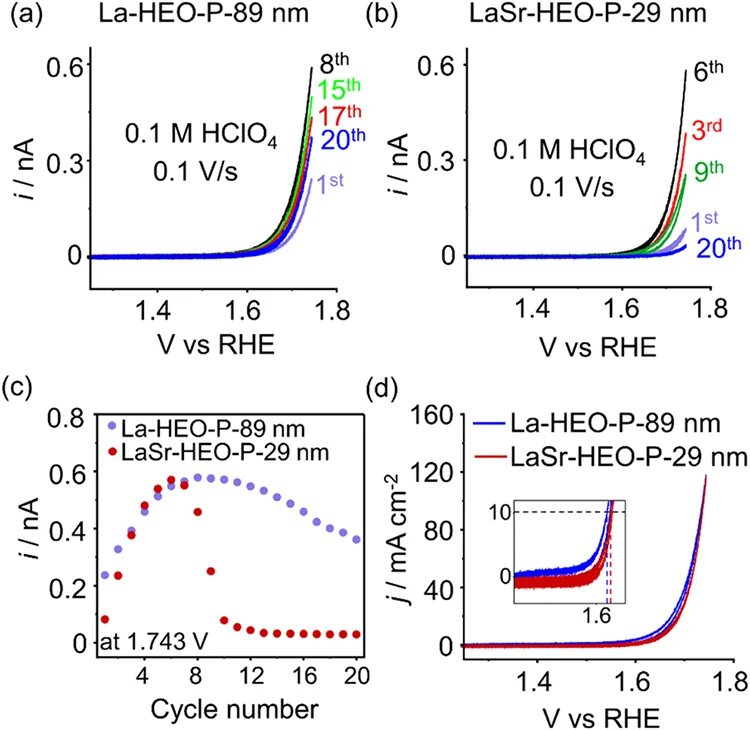
(a) OER cyclic voltammetry (CV) curves for La-HEO-P-89 nm from CV cycling; (b) CV curves for LaSr-HEO-P-29 nm from CV cycling; (c) Plot of currents for La-HEO-P-89 nm and LaSr-HEO-P-29 nm with respect to different CV cycles; (d) Overlay of CV curve (highest *i*–*E*) for La-HEO-P-89 nm and LaSr-HEO-P-29 nm, where, pipet radius was used to normalize the current.

Chronoamperometric *i–t* transients similarly exhibit a pronounced maximum followed by decay during single landings for both HEO-P films (Figure S14). While the anodic current reflects water oxidation, these measurements are performed under conditions where perovskite dissolution is thermodynamically and kinetically accessible (high oxidative bias and low pH). As a result, the observed activation–deactivation and the transient “current humps” (CV and *i–t*) can arise from concurrent surface change, e.g., dissolution pit formation. The current, therefore, will arise from a combination of dissolution and OER, as depicted in Figure 3a.

**Figure 3:**
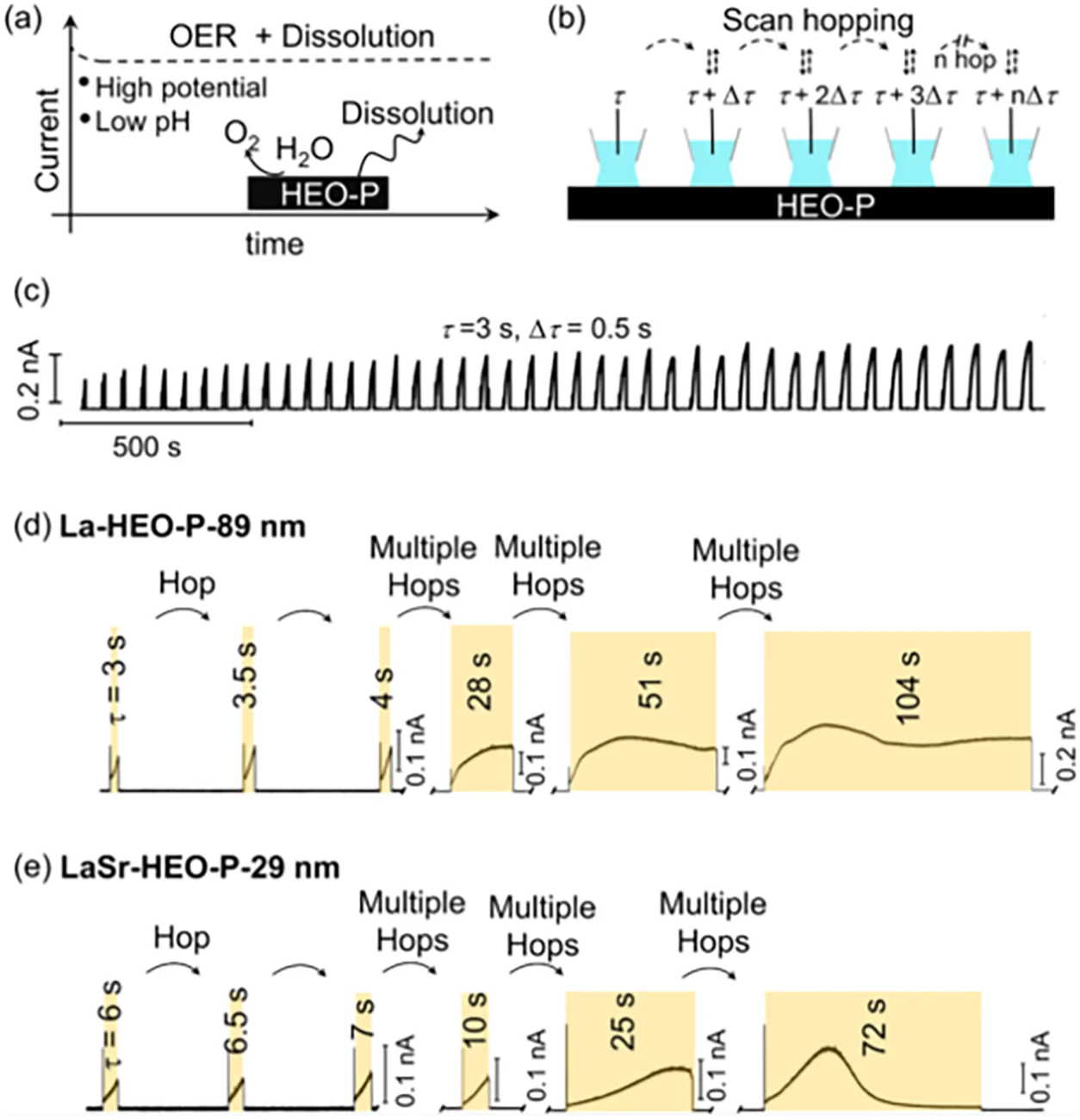
(a) Illustration of dissolution coupled water oxidation under corrosive environment; (b) Schematic of *i*–*t* scanning of HEO-P as a function of pulse time (τ) by nanopipet hop over the electrode; (c) Representative *i*–*t* trace from single scan-hopping experiments on La-HEO-P-89 nm electrode (0.1 M HClO_4_, overpotential of 475 mV). Zoomed version of *i*–*t* plots at different τ (yellow shaded) during hops over La-HEO-P-89 nm (d) and LaSr-HEO-P-29 nm.

A key advantage of our study is that the HEO-P electrodes are atomically smooth, binder-free, and single-facet films. This eliminates other common contributions (carbon oxidation, binder degradation, and multifaceted heterogeneity) and allows the oxidative current to be interpreted in terms of processes intrinsic to the perovskite. Droplet-confined electrolysis on the flat geometric film leaves a dissolution footprint after each electrochemical operation, as evidenced by AFM imaging following the SECCM measurements. This method can reveal how the pit develops and changes as a function of time.

To understand how dissolution pits grow with time, we performed a series of *i–t* experiments in hop–scanning mode, where the duration of the pulse (τ) was systematically increased in small steps (Δτ). Each pulse was applied at a new location on the HEO-P surface so that every *i–t* scan started on a fresh and pristine area (Figure 3b). For both catalysts, we covered the full τ–window by running several hop–scanning sequences over different pulse-time segments and then stitching these segments together to create a continuous τ–profile. This time-resolved protocol was carried out for both La-HEO-P-89 nm and LaSr-HEO-P-29 nm at an overpotential η = 475 mV (using 1.23 V vs RHE as the *E* ^0^ for the OER), which was chosen to access an optimal kinetic window: at lower η, pit initiation is too sluggish to observe within practical pulse durations, whereas at higher η, dissolution becomes too fast to resolve early pit growth.

For La-HEO-P-89 nm, we examined a broad τ range of 3–126 s by combining four scan-hopping sequences where short to intermediate durations (3–77 s) were sampled with fine increments (Δτ = 0.5 s), while longer times (77–126 s) used a larger increment (Δτ = 1 s). LaSr-HEO-P-29 nm was probed over a τ–range of 6–72 s (assembled from three scan-hopping sequences) using a uniform increment of 0.5 s, providing sufficient temporal resolution to reliably capture the pit growth process (Figures S15 and S16). Figure 3c represents an *i–t* trace from a single scan hopping experiment for La-HEO-P-89 nm. A few representative scans at different τ scales have been provided in Figure 3d,e. Notably, shorter pulses reproduce the corresponding early portion of the full current profile, indicating that the current evolves along a deterministic trajectory as a function of τ for a given set of conditions.

To correlate electrochemical pulses to the underlying surface responses, we next correlate each landing event with its postreaction footprint. Figure 4 shows a representative SEM image of the La-HEO-P-89 nm surface after the scan-hopping experiment, where darker spots correspond to the reaction footprints. The inset in Figure 4 highlights the hopping pattern during the pipet trajectory. After SECCM scanning, the same regions were imaged by AFM, which resolves the three-dimensional topography of each footprint and quantifies the resulting surface morphological change (Figures S17 and S18). The image shows that each landing produced a well-resolved pit, suggesting that the HEO-P electrodes undergo uniform dissolution rather than particle exsolution, supported by EDS spectroscopy (Figure S19).

**Figure 4:**
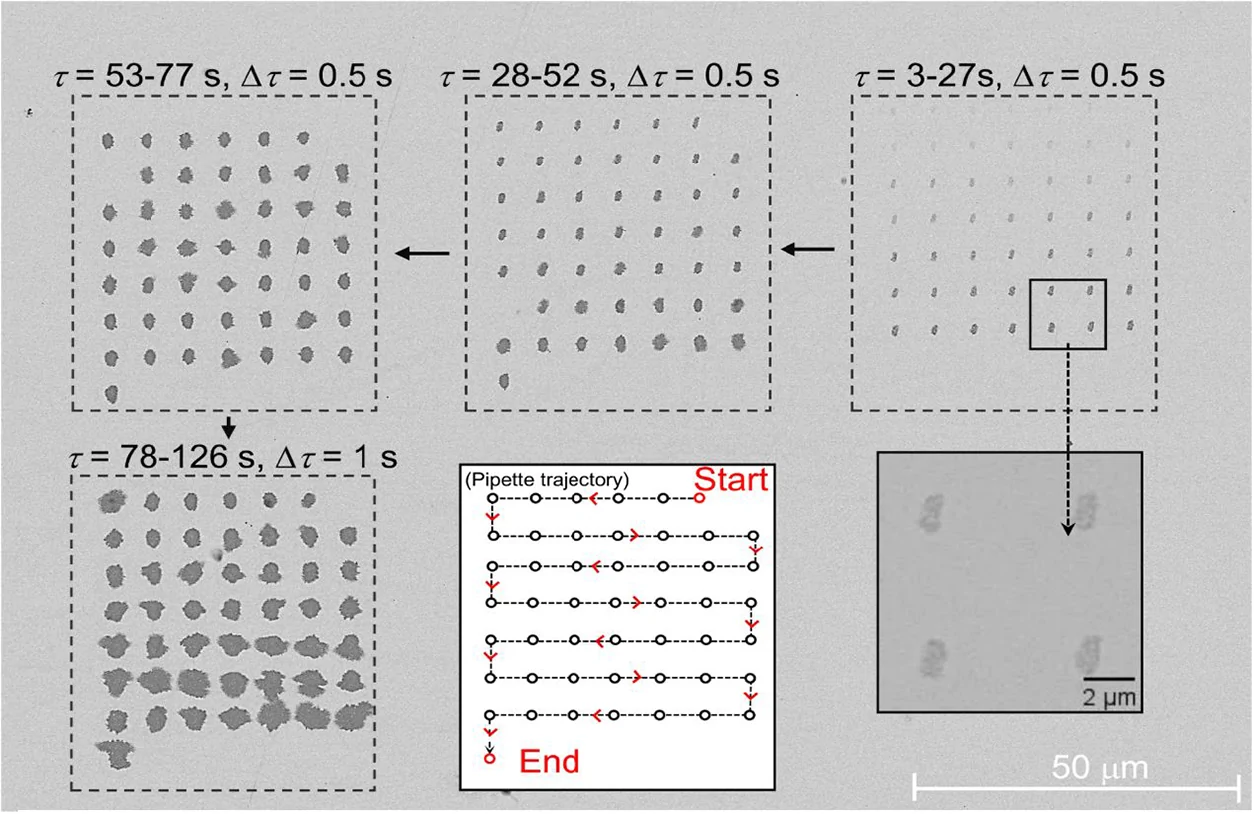
Representative SEM image of the La-HEO-P-89 nm electrode after nanopipet hops as a function of τ. The dashed boxes indicate the regions where measurements were performed at different τ and Δτ values. The hopping trajectory is shown in white shaded box. The experiment was done using a single pipet (*d*_pipet_ = ∼800 nm).

Pit geometry was extracted from AFM topography to obtain depth, surface area, and volume (Section S3 and Figure S20). The pulse duration associated with each pit was assigned by overlaying the pipet trajectory onto the AFM map, enabling accurate assignment of τ to their corresponding pit geometry (Tables S2 and S3). This procedure provides a quantitative measure between electrochemical pulse duration and pit depth, area, and volume as a function of τ. Figure 5a,b show representative AFM line profiles of the dissolution pits as a function of τ. The corresponding pit depth vs τ plots for La-HEO-P-89 nm (Figure 5c) and LaSr-HEO-P-29 nm (Figure 5d) reveal a progressive attenuation in film thickness with increasing τ, followed by saturation at a depth comparable to the initial film thickness previously determined by XRR (Figure S3). Analysis of the pit surface area as a function of τ further indicates faster lateral dissolution for La-HEO-P-89 nm relative to LaSr-HEO-P-29 nm (Figure S21). Collectively, these results demonstrate that pit growth proceeds through coupled vertical deepening and lateral spreading with increasing τ.

**Figure 5:**
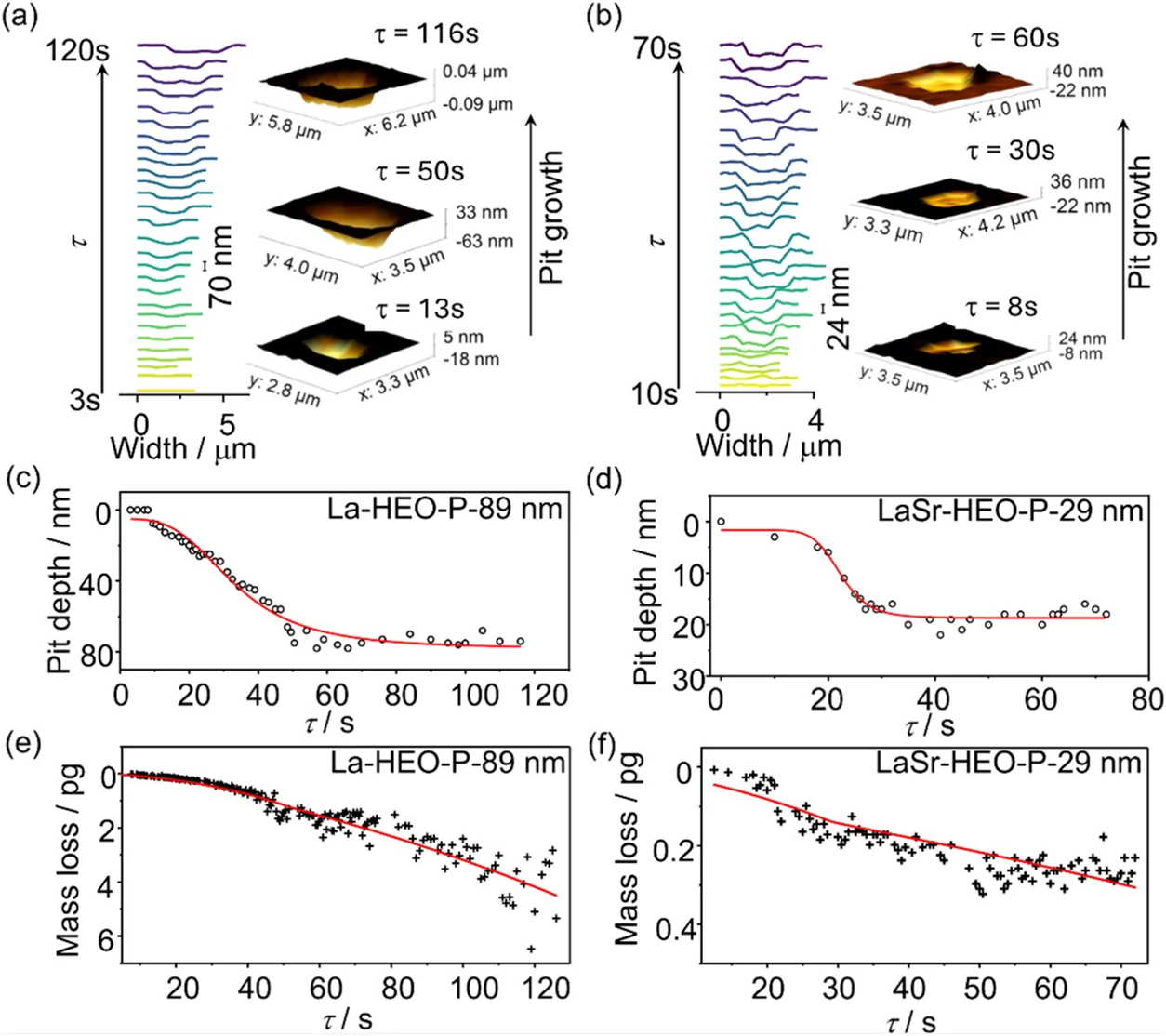
(a) *Ex-situ* characterization of La-HEO-P-89 nm electrode after experiments using Atomic Force Microscopy (AFM). Left panel is the overlay of AFM line scans from individual dissolution pits, and the right panel is three-dimensional visualization of pits at intermediate τ in-between 3–126 s window; (b) *Ex-situ* characterization of LaSr-HEO-P-29 nm electrode after experiments using AFM. Left panel is the overlay of AFM line scans from individual dissolution pit, and the right panel is the three-dimensional visualization of pits at intermediate τ in-between 6–72 s window; (c) Dissolution pit depth vs τ plot for La-HEO-P-89 nm, and for LaSr-HEO-P-29 nm (d). Mass loss of HEO-P vs τ plot for La-HEO-P-89 nm (e), and for LaSr-HEO-P-29 nm (f). Solid lines are fits to the pit-growth kinetic model introduced in the main text (see Figure 7) and described in detail in Methods; they are shown here to guide interpretation of the τ-dependence.

The mass loss vs τ plots are shown in Figure 5e,f for La-HEO-P-89 nm and LaSr-HEO-P-29 nm, respectively, where the mass dissolved can be estimated from the pit volumes and density for both HEO-P (Section S4). Despite larger mass loss in La-HEO-P-89 nm, absolute dissolution is not a transferable stability metric due to differences in thickness, evolving surface area, and charge throughput. Normalization to the oxidative charge per pulse is therefore required.

We make this normalization explicit by separating the dissolution pathway from water oxidation through charge partitioning (Figure 6a,b). Specifically, we define *Q*_total_ as the total charge resulting from OER and lattice dissolution. We delineate *Q*_total_ into a contribution attributable to lattice dissolution by estimating *Q*_pit_ from the mass loss (Δ*m*_pit_) using a conservative maximal-oxidation accounting based on Pourbaix diagrams, which involves oxidation of both the *B*-site cations (Figure S22) and the lattice oxide (O^2–^) (Section S4 and Table S4). This yields an upper bound of *Q*_pit_. *Q*_total_ is estimated from each landing by integrating the corresponding SECCM *i-t* transient, *Q*_total_ = *∫ i*(*τ*)­d*t*.

**Figure 6:**
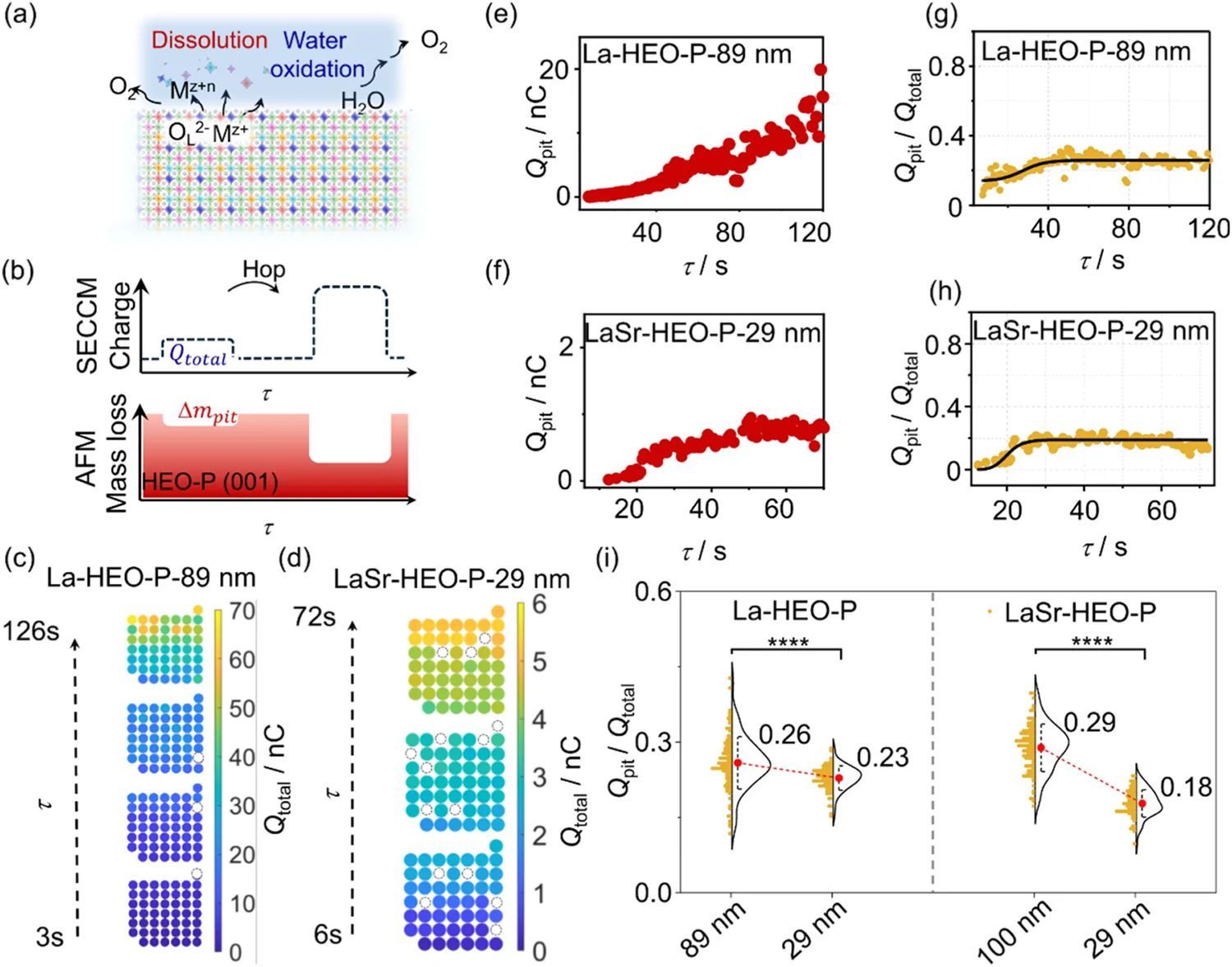
Schematic for decoupling dissolution and water oxidation from colocating SECCM–AFM area (a and b); (c, d) *i*–*t* charge (*Q*_total_ = *∫i*(τ)­d*t*) mapping on La-HEO-P-89 nm from τ = 3–126 s (left panel), on LaSr-HEO-P-29 nm from τ = 6–72 s (right panel); (e) *Q*_pit_ as a function of τ for La-HEO-P-89 nm; (f) *Q*_pit_ as a function of τ for LaSr-HEO-P-29 nm; (g) *Q*_pit_/*Q*_total_ as a function of τ for La-HEO-P-89 nm; (h) *Q*_pit_/*Q*_total_ as a function of τ for LaSr-HEO-P-29 nm; (i) Half Violin plots of *Q*_pit_/*Q*_total_ for La-HEO-P-89 nm, La-HEO-P-29 nm, LaSr-HEO-P-100 nm, and LaSr-HEO-P-29 nm where, yellow solid circles show individual data points (*N* = 116 for La-HEO-P-89 nm, 85 for La-HEO-P-29 nm, 101 for LaSr-HEO-P-100 nm and 78 for LaSr-HEO-P-29 nm) and the black curve indicates data density, with the red circle and dotted bar representing the mean value and ± 1standard deviation, respectively.

Figure 6c,d show heatmaps of *Q*_total_ for La-HEO-P-89 nm and LaSr-HEO-P-29 nm, respectively. Each circle corresponds to a single landing within a hop-scanning sequence, arranged in 7 × 7 spots over an area of ∼36 × 36 μm^2^. The landing sequence can be followed by tracing the circles along the pipet trajectory, from the first landing (bottom) to the last landing (top), following the pattern shown in the inset of Figure 4. A gradual accumulation of *Q*_total_ was observed at short-to-intermediate τ, consistent with the expected behavior for atomically smooth, single-facet films under controlled droplet confinement. However, at longer τ, *Q*_total_ exhibits significant dispersion. We attribute this increasing variability primarily to electrolyte spreading beyond the confined meniscus footprint after prolonged contact.

We quantify dissolution by estimating *Q*_pit_ for each landing and plotting it as a function of τ (Figure 6e,f for La-HEO-P-89 nm and LaSr-HEO-P-29 nm, respectively). *Q*_pit_ shows a similar trend to *Q*_total_ across the τ profile. Because *Q*_pit_ and *Q*_total_ are correlated across landings, their ratio (Figure 6g,h), makes charge partitioning a more robust basis for comparing the branching between OER and lattice dissolution.

Partitioning *Q*_total_ into charge consumed by lattice dissolution (*Q*_pit_) and water oxidation yields lower-bound estimates of the OER faradaic efficiency, FE_OER_ = 1 – *Q*_pit_/*Q*_total_, of ∼74% for La- HEO-P-89 nm and ∼82% for LaSr-HEO-P-29 nm (Figure S23). We therefore use the dimensionless ratio *Q*_pit_/*Q*_total_ to quantify dissolution propensity and its evolution with τ. For both electrodes, *Q*_pit_/*Q*_total_ initially increases and then stabilizes (Figures 6e,h). The mean ratio is 0.26 ± 0.03 for La-HEO-P-89 nm and 0.18 ± 0.02 for LaSr-HEO-P-29 nm, indicating that La-HEO-P-89 nm has ∼44% greater dissolution propensity. Thus, Sr substitution suppresses lattice dissolution and directs a larger fraction of the oxidative charge toward OER.

While these results indicate La-HEO-P-89 nm and LaSr-HEO-P-29 nm have different dissolution propensities, the two electrodes also differ in film thickness. To determine whether the observed differences originate from composition rather than thickness of the electrode, we prepared two additional HEO-P electrodes with near-complementary thicknesses, a thinner La-HEO-P (∼29 nm, La-HEO-P-29 nm) and a thicker LaSr-HEO-P (∼100 nm, LaSr-HEO-P-100 nm) to isolate the influence of film thickness on dissolution and OER kinetics. Following our method under identical conditions (Figures S24 and S25), the *Q*_pit_/*Q*_total_ value was estimated to be 0.23 ± 0.01 and 0.29 ± 0.03 for La-HEO-P-29 nm and LaSr-HEO-P-100 nm, respectively. All the *Q*_pit_/*Q*_total_ values (after stabilization) for both HEO-P were presented in half violin plots, where the left half of the violin represents the kernel density distribution of *Q*_pit_/*Q*_total_ (Figure 6i).

To assess whether the evolution of *Q*_pit_/*Q*_total_ is intrinsically related to the dissolution/OER branching or merely reflects film perforation, we correlated the evolution of *Q*_pit_/*Q*_total_, *Q*_total,_ and pit depth as a function of τ. Figures S26 and S27 show, respectively, that *Q*_pit_/*Q*_total_ reaches a stable value well before film perforation for La-HEO-P-29 nm (as indicated by the vertical dashed line), whereas stabilization occurs immediately before perforation for LaSr-HEO-P-100 nm. These results indicate that stabilization of *Q*_pit_/*Q*_total_ and film perforation are independent phenomena. We speculate that the initial rise of *Q*_pit_/*Q*_total_ is due to dissolution involving slow nucleation and growth.

The scan-hopping protocol was extended across a range of η to probe how dissolution kinetics depend on driving force. For each HEO-P electrode, we collected *i–t* traces at different η ranging from 414 to 514 mV. At each η, we collected *i–t* traces over an optimized τ–window chosen to capture the evolution of pit geometry (Figures S28–S31). Representative SEM images of the postexperiment surfaces show the footprints of the scan-hopping area (white dashed square in Figures S32 and S33) for different η. AFM imaging of every scan-hopping region (Figures S34–S37) provides a quantitative pit-geometry data set (depth and volume) as a function of τ and η. The pit-depth vs τ plot demonstrates the electrochemical nature of the dissolution process, with pit growth exhibiting a strong dependence on η (Figure S38).

This trend is mirrored in terms of total oxidative charge, *Q*_total_ (τ), and the dissolution-associated charge, *Q*_pit_ (τ), at each η. For La-HEO-P-89 nm and La-HEO-P-29 nm, *Q*_pit_/*Q*_total_ ranges from 0.20 ± 0.06 to 0.24 ± 0.02 and 0.21 ± 0.04 to 0.24 ± 0.02 respectively, while LaSr-HEO-P-29 nm and LaSr-HEO-P-100 nm ranges from 0.11 ± 0.03 to 0.17 ± 0.03 and 0.32 ± 0.02 to 0.28 ± 0.01 respectively over η *=* 454–514 mV. Notably, *Q*_pit_/*Q*_total_ shows no significant dependence on η for all HEO-P electrodes (Figures S39–S42). The overall observation indicates that changes in overpotential primarily change the overall extent of oxidation, with only a limited shift in branching between OER and dissolution. In contrast, film thickness exerts a composition-dependent influence on this branching, with La-HEO-P exhibiting minimal thickness sensitivity, whereas LaSr-HEO-P shows a pronounced thickness dependence.

To estimate the effect of [H^*+*^] on HEO-P electrode dissolution, La-HEO-P-29 nm and LaSr-HEO-P-100 nm were examined using ∼25 mM HClO_4_ filled pipet at the same OER overpotential employed in 100 mM HClO_4_, resulting in measurable pit growth (Section S7, Figures S43 and S44) at both pH. We focused on a modest variation of pH because this narrow window already produces a measurable suppression of dissolution while allowing the same SECCM pulse protocol. Additionally, keeping the small difference in pH reduces the likelihood of crossing Pourbaix boundaries, where changes in stable oxidation states and dissolution products could alter dissolution stoichiometry.

The scaling between *Q*_pit_ and *Q*_total_ on both temporal (after the initial transient) and the potential window suggests that the lattice dissolution and the OER scale proportionally under anodic polarization, i.e., that dissolution proceeds in the same kinetic regime as water oxidation rather than through a separate, independently rate-limiting pathway. However, the dissolution current cannot be delineated from the total current. Using Faraday’s law, the rate of dissolution can be expressed in terms of the current density *j*_*d*_, *V*_pit_ and *A* (time-dependent evolution of pit volume and liquid–solid contact area)

1 $$\frac{\Delta \left(V_{pit}/A\right)}{\Delta \tau }=\frac{M_{\mathrm{HEO}‐P}}{\rho_{\mathrm{HEO}‐P}nF}j_{d}\equiv k_{d}\left({\mathrm{cm}\;s}^{-1}\right)$$

where ρ_HEO‑P_ and *M*_HEO‑P_ is the density and molar mass of the HEO-P, respectively, and *n* is the number of electrons transferred per formula unit. Equation 1 also defines the dissolution rate constant, *k*_d_. Our measurements of pit growth indicate that *k*_d_ depends on pH and potential. Based on these dependencies, we propose the following expression for *k*_d_

2 $$k_{d}=k_{d}^{0}10^{-b\mathrm{pH}}\exp\left[\frac{\alpha F\eta }{RT}\right]$$

In eq 2, *k*_d_^0^ corresponds to the *k*_d_ at pH = 0 and η = 0. In this form, *b* sets the pH-dependence of log_10_ *k*_d_:

3 $$b=-\frac{\partial {\;\log}_{10}\;k_{d}}{\partial \mathrm{pH}}$$

This makes *b* straightforward to extract from the plot of log_10_ *k*_d_ vs pH at fixed η, exactly as α is estimated from the plot of ln *k*_d_ vs η at fixed pH. In the following, we extract *k*_d_ by modeling the advancing dissolution front using AFM images obtained during pit growth.

We model the evolution of the pit geometry using an axisymmetric geometry that captures the vertical and lateral advancement of the dissolution front (Figure 7). The model assumes a constant *k*_d_, resulting in the pit base advancing downward while the pit opening expands radially. Because the meniscus continuously fills the evolving cavity, the liquid–solid contact area always matches the pit opening. Once the HEO-P layer is perforated, i.e., when the receding base reaches the insulating/nonreactive substrate at the contact time τ_c_, further dissolution cannot increase the physical depth, and the pit evolves predominantly laterally.

**Figure 7:**
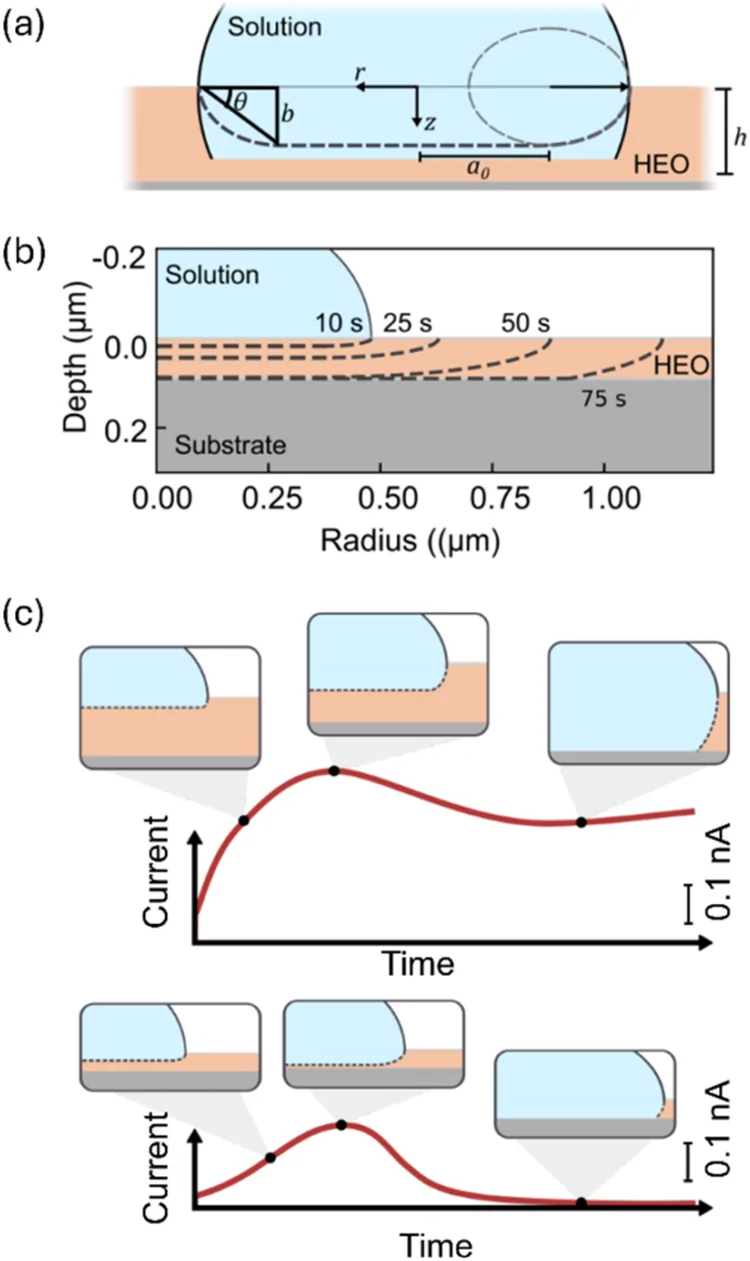
(a) Geometric schematic of the pit model: footprint radius *a*_0_, base depth *b*(τ) = *k*_dt_, wall angle θ, and film thickness *h*; (b) Cross-sectional geometry at increasing times, shown as a half-slice (axial symmetry), with the droplet above and the substrate at *z* = 0. The equations that describe the pit geometry and time evolution are presented in the method section (*vide infra*); (c) Representative SECCM *i*–*t* trace, replotted for La-HEO-P-89 nm (top) and LaSr-HEO-P-29 nm (bottom), HClO_4_ 100 mM and η = 475 mV. Inset shows the pit geometries inferred from the pit-growth model at three times: early growth (τ < τ_c_), near the current maximum (τ ≈ τ_c_), and after perforation (τ ≥ τ_c_). The current maximum correlates with the modeled contact time, when the pit base reaches the substrate, and the growth mode transitions from vertical deepening to predominantly lateral expansion.

To reproduce the experimentally observed anisotropy (faster lateral expansion than vertical advance), we introduce a single shape parameter, the wall angle, θ, and represent the surface by revolving an elliptical arc in the *r–Z* cross-section (Figure 7a,b). This construction yields closed-form, piecewise expressions for the pit area and volume in the precontact (τ < τ_c_) and postcontact (τ ≥ τ_c_) regimes (eqs 6–17 in the Experimental and Method section). The geometric model provides a direct physical interpretation of the characteristic “hump” observed in the SECCM current transients. Figure 7c replots the representative *i–t* traces for HEO-P-89 nm and LaSr-HEO-P-29 nm and overlays snapshots of the pit geometry inferred by the model at three key times: early growth (τ < τ_c_), near the current maximum (τ ≈ τ_c_), and after perforation (τ ≥ τ_c_).

In the model, the exposed contact area between HEO-P and the droplet initially increases as the pit deepens and the three-dimensional wall area develops, producing a rising current. As the pit base reaches the substrate (inert toward OER) at the contact time τ_c_, pit growth transitions from predominantly vertical to predominantly lateral growth. This geometric transition reduces the fraction of oxide actively exposed within the droplet footprint, consistent with both the transient current response observed in the *i*–*t* curves and the activation/deactivation process on the localized SECCM CVs (Figure 2). At longer times, the residual current is controlled by how much oxide remains available for continued dissolution/oxidation under the droplet. Accordingly, the thicker La-HEO-P-89 nm film sustains a larger posthump current than the thinner LaSr-HEO-P-29 nm, consistent with the AFM-measured depths approaching the layer thickness and with the model’s postcontact regime (τ ≥ τ_c_) in which additional charge drives lateral expansion rather than further deepening. We fit the simulated geometry (initial opening radius and wall angle) model to the AFM pit volumes data set to estimate *k*_d_ at each η for all HEO-Ps electrodes (Figure 8a–d). In doing so, each overpotential yields a best-fit dissolution rate that is directly tied to the independently measured pit geometry. Then a plot of the *k*_d_ values as a function of η was fitted using the rate law expression (Figure 8e).

**Figure 8:**
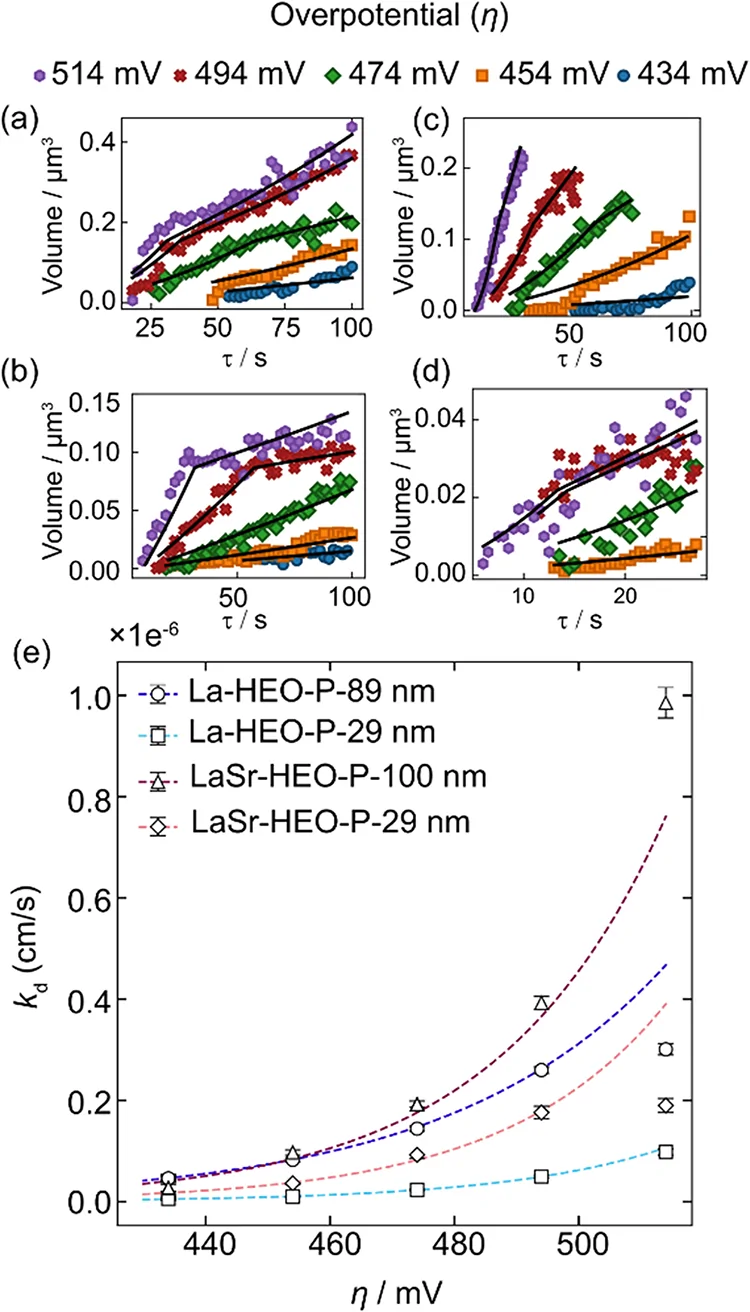
(a) Pit volume *V*(τ) from AFM scan-hopping (100 mM HClO_4_) for the La-HEO-P-89 nm; (b) for La-HEO-P-29 nm; (c) for LaSr-HEO-P-100 nm; (d) for LaSr-HEO-P-29 nm electrode at different η (symbols). Black lines are best fits of the volume-evolution model using a single geometry across data sets, while the dissolution rate is allowed to vary with potential. (e) Dissolution rates extracted from panels (a–d) versus overpotential, with the fitted electrochemical kinetic relation (eq 2) for HEO-Ps, respectively. The *k*_d_ values at η = 514 mV for La-HEO-P-89 nm and LaSr-HEO-P-29 nm were excluded due to mass transport limitations under aggressive OER.

Using eq 2, the linear fit of the log (*k*_d_) vs η (at pH ∼1) plot yields α and the apparent rate constant (*k*_d_^0^ × 10^–*b*×1^) (pH = 1, η ≈ 0). Independently, we estimated *b* by measuring *k*_d_ at two different pH (∼1 and 1.6) at constant η which was used for estimating *k*_d_^0^ (pH = 0, η ≈ 0) for all HEO-P electrodes (Table 1).

**Table 1:** Parameters for Electrochemical Dissolution of HEO-P Electrodes

electrode	film thickness (nm)	*k*_d_^0^ × 10^−*b*×1^ (×10^–14^/cm s^–1^)^a^	α^a^	*b* ^b^	*k*_d_^0^(× 10^–14^/cm s^–1^)^c^
La-HEO-P	∼89	18	0.74		153
	∼29	0.028	0.99	0.93	0.24
LaSr-HEO-P	∼100	0.52	0.94	0.49	1.6
	∼29	0.08	1		0.25

a Values were reported as best-fit ± 92% confidence interval (Figure 8e).

b See Section S7 for details.

c Values were calculated using values determined from La-HEO-P-29 nm and LaSr-HEO-P-100 nm electrodes

Notably, reduction of film thickness from ∼89 to ∼29 nm suppressed *k*_d_^0^ by × ∼650-fold for La-HEO-P, whereas only × ∼6-fold suppression was observed for LaSr-HEO-P upon reducing the thickness from ∼100 to ∼29 nm. In contrast, the effect on the dissolution propensity differed markedly between the two compositions. The *Q*_pit_/*Q*_total_ (median value for η *=* 454–514 mV) ratio remained essentially unchanged for La-HEO-P upon reducing the thickness from ∼89 to ∼29 nm, whereas LaSr-HEO-P exhibited an ∼2-fold decrease in *Q*_pit_/*Q*_total_ when the thickness was reduced from ∼100 to ∼29 nm. Overall, film thinning influences the two HEO-P compositions in fundamentally different ways: La-HEO-P exhibits dramatic kinetic stabilization with little to no change in the dissolution branching ratio, whereas LaSr-HEO-P undergoes only modest kinetic stabilization but a significant shift in reaction branching from lattice dissolution toward OER.

To gain qualitative insight into the OER pathway on both catalysts, we examined the pH dependence of the OER current on the RHE scale (Figures S45 and S47). Specifically, we plotted log *j* as a function of pH at fixed V (vs RHE) and extracted the apparent proton reaction order, *b*_OER_ = (∂ log *j*/∂pH)_RHE_.This analysis gives *b*_OER_ = −0.41 ± 0.03 for La-HEO-P, and −0.19 ± 0.03 for LaSr-HEO-P (Figures S46 and S48), consistent with *b* value trend estimated from the dissolution channel. Consistently, the overpotential shift with pH at fixed current density, (∂V/∂pH)_*j*_, is larger for La-HEO-P (0.048 ± 0.004 V/pH) than for LaSr-HEO-P (0.016 ± 0.003 V/pH), indicating a stronger pH sensitivity of OER kinetics (non-PCET) for La-HEO-P than that of LaSr-HEO-P.^34–36^

To probe whether lattice oxygen redox may be implicated under OER cycling, we analyzed the O 1s XPS spectra of both HEO-P. The spectra shown in Figure S49, were deconvoluted into lattice oxygen (O^2–^, 528.13–528.59 eV), oxidized oxygen species (O^–^/O_2_^2–^, 528.68–529.03 eV), and chemisorbed water oxygen (H_2_O, 531.32–532 eV) (Table S6). Both HEO-P catalysts exhibit a substantial contribution from oxidized oxygen species, where LaSr-HEO-P shows a slightly higher fraction of O^–^/O_2_^2–^ than that of La-HEO-P.^37–39^ Together with the nonzero *b*_OER_ values, the presence of O^–^/O_2_^2–^ species provides a qualitative indication of lattice oxygen participation (LOP) during OER cycling for both HEO-P. In addition, *Q*_pit_ scales with *Q*_total_, and the dissolution fraction *Q*_pit_/*Q*_total_ exhibits near independence of η. Collectively, all the observations demonstrate that OER and dissolution accelerate similarly with η, sharing a kinetically relevant intermediate (or set of closely related intermediates).

We propose a qualitative scheme to describing dissolution-coupled OER (Figure 9a), where both catalysts are suggested to follow the lattice oxygen-mediated (LOM) OER pathway, in agreement with previous studies.^40–42^ In this scheme, a fully coordinated surface motif can enter a lattice-oxygen branch leading to O–O bond formation and O_2_ evolution. Along this pathway, formation of an undercoordinated intermediate (schematized as V_O_–M–V_O_) provides a bifurcation point: it can be replenished by a water molecule and enter into the OER cycle (e.g., V_O_–M–V_O_ → O–M–V_O_), or it can proceed down a dissolution branch via limited V_O_ compensation and result in the formation of a dissolution pit. The replenishment of V_O_ by H_2_O molecules and the H^+^ transfer pathway in acidic electrolytes differs fundamentally from the alkaline OER pathway. In acid, the refilling of V_O_ by H_2_O is intrinsically hindered because the strongly hydrogen-bonded interfacial water structure restricts the passage of neutral H_2_O molecules across the inner Helmholtz plane to the catalyst surface. In contrast, H^+^ transport proceeds rapidly through the hydronium–water network via the Grotthuss hopping mechanism. Consequently, vacancy compensation becomes a proton-dependent and kinetically limiting interfacial step and causes overall catalyst stabilization.^43,44^

**Figure 9:**
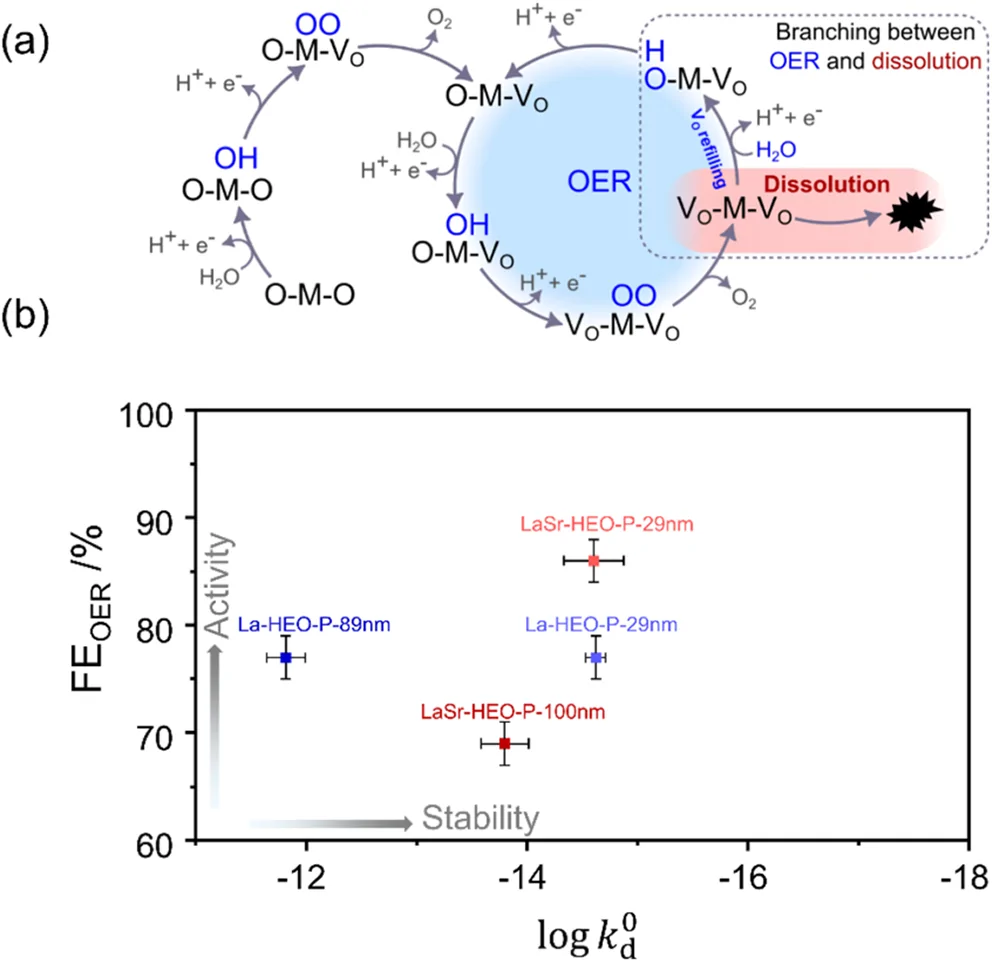
(a) Proposed mechanism for LOM-based coupling between OER and dissolution; (b) correlation between faradaic efficiency and *k*_d_^0^ for HEO-P electrodes.

However, the origin and electrochemical consequence of this stabilization differ between the 30% Sr^2+^ doped and nondoped electrodes. For La-HEO-P, the thinner film exhibits markedly improved dissolution resistance without a significant change in faradaic yield, indicating that film thinning suppresses OER and lattice dissolution to a comparable extent while largely preserving the charge partitioning between the two processes. In contrast, for LaSr-HEO-P, thinning the film not only stabilizes the catalyst, albeit to a more moderate extent, but also increases the faradaic yield (Figure 9b), indicating a redistribution of charge from lattice dissolution toward OER.

Aliovalent substitution of Sr^2+^ at the La^3+^ site introduces *A*–site cation deficiency, which modifies the electronic structure of the *B*O_6_ framework (*B* = Cr, Mn, Fe, Co, Ni) and promotes lattice oxygen redox and introducing oxygen nonstoichiometry.^45–48^ Consequently, the Sr-doped films are expected to exhibit an oxygen-vacancy concentration gradient, as progressive strain relaxation beyond a critical film thickness modifies the defect distribution throughout the film.^49^ This results in a strong coupling between film thickness and lattice oxygen/vacancy dynamics. Reducing the film thickness likely limits subsurface defect accumulation while facilitating more efficient utilization of oxidizing equivalents for oxygen evolution, thereby shifting the reaction branching away from lattice dissolution and toward OER.

## Conclusion

In conclusion, we establish a combined SECCM–AFM workflow that directly quantifies catalyst dissolution during acidic OER at the nanoscale. Localized droplet-confined electrochemical pulses on atomically smooth, single-facet perovskite oxide films generate reproducible dissolution pits, whose depth, area, and volume can be measured by AFM as functions of pulse duration, overpotential, and pH.

By integrating SECCM *i–t* transients to obtain the total oxidative charge *Q*_total_ and converting AFM-derived mass loss into a theoretical dissolution charge (*Q*_pit_), we quantitatively separate the dissolution channel from oxygen evolution. This enables direct estimates of the OER faradaic efficiency, providing a rigorous basis to resolve electrodes under identical microscopic reaction environments.

To investigate electrochemical stimuli-driven pit growth kinetics, we introduce a pit-geometry model coupled with a rate-law description of dissolution, yielding effective electrochemical kinetic descriptors (*k*_*d*_^0^, reaction orders, and an apparent transfer coefficient). Applying this framework to isostructural La-HEO-P and 30% Sr-substituted La-HEO-P (LaSr-HEO-P) provides a quantitative correlation of the relationship between aliovalent doping and OER stability and benchmarking them under standard conditions.

We also obtain mechanistic insights from experiments using electrodes of different thicknesses. The proportional scaling of *Q*_pit_ and *Q*_total_ across η, and τ indicates that OER and dissolution operate within the same kinetic regime and share a common branching intermediate. The pH-dependent OER and dissolution behavior, together with the presence of O^–^/O_2_^2–^ species identified by XPS, supports that the HEO-P catalyst most likely involves lattice-oxygen participation, where the balance between V_O_ generation (dissolution ) vs V_O_ compensation (electrolyte refilling) decides overall catalyst stability. Analysis at different thickness levels unravels that reducing film thickness suppresses the intrinsic dissolution kinetics (*k*_d_^0^) in both HEO-P systems but through composition-dependent stabilization pathways. For LaSr-HEO-P, the suppression of *k*_d_^0^ is accompanied by a concurrent decrease in *Q*_pit_/*Q*_total_, indicating improvement in OER faradaic efficiency. In contrast, the substantial suppression of *k*_d_^0^ for La-HEO-P, accompanied by little to no change in *Q*_pit_/*Q*_total_ indicates enhanced resistance toward dissolution without altering the partitioning of charge toward OER.

Overall, our decoupled analysis using combined SECCM–AFM approach provides a powerful platform for delineating and quantifying lattice dissolution during OER, thereby enabling systematic investigation of the interplay among electrode design, dissolution kinetics, charge partitioning, and catalyst structure. The resulting mechanistic insights offer valuable design principles for developing more durable OER electrocatalysts and guiding their translation to bulk-scale electrodes. Extending the approach to combinatorial or composition-gradient electrode libraries could enable high-throughput screening and quantitative benchmarking across broad compositional spaces for activity–stability optimization.

### Experimental and Methods

### Chemicals and Materials

Perchloric acid (HClO_4_, Sigma-Aldrich, 70%), potassium chloride (KCl, Sigma-Aldrich, 99%), and sodium perchlorate (NaClO_4_, Sigma-Aldrich, ≥98%) were used as supplied by the manufacturer. All solutions were prepared using 18.2 MΩ cm water purified using a Barnstead Smart2Pure system.

### HEO-P PLD Growth

La-HEO-P (001, ∼89 and ∼29 nm) and LaSr-HEO-P (001, ∼100 and ∼29 nm) were synthesized using pulsed laser deposition (PLD) technique from stoichiometric single-phase ceramic targets onto Nb-SrTiO_3_ (001) substrates. Deposition was carried out using a KrF excimer laser with a fluence of 0.85 J/cm^2^ and a pulse rate of 5 Hz, with a 5 cm distance between the target and substrate. The films were grown at an oxygen pressure of 90 mTorr and substrate temperatures of 625 °C for La-HEO-P and 635 °C for LaSr-HEO-P, respectively. Following deposition, the samples were cooled to room temperature under 200 Torr of oxygen.^50,51^

### Probe Fabrication

Nanopipets used for all SECCM experiments were fabricated using a Sutter P-2000 laser puller (Sutter Instrument Co.) from glass capillaries (O.D. One mm, I.D. 0.7 mm). The pulling parameters were set as *Heat* = 475, *Filament* = 3, *Velocity* = 30, *Delay* = 145, and *Pull* = 170, yielding two nearly identical pipets with tip radii in the range of ∼ 375 – 450 nm. The nanopipet tip radius (*r*_pipet_) was estimated electrochemically by recording the ionic current–voltage (*i–E*) response in 0.1 M KCl, with silver chloride-coated silver wire (Ag/AgCl) quasi-reference counter electrodes (QRCE) placed inside the pipet and in the bulk solution. The slope of the linear *i–E* response corresponds to the pipet conductivity κ, which is related to the pipet radius by the following expression:^52^

4 $$r_{\mathrm{pipet}}=\frac{1}{\kappa R_{P}}\left(\frac{1}{4}+\frac{1}{\mathrm{\pi tan}\;\theta }\right)$$

where, κ is the electrolyte conductivity (0.0116 S/cm for 0.1 M KCl, 20 °C), *R*_p_ is the measured resistance from the *i–E* curve, and θ is the cone angle of the nanopipet (∼10°)

### Scanning Electrochemical Cell Microscopy (SECCM)

SECCM is a well-established technique that is described in literature.^53^ All experiments were done using a home-built SECCM setup. The nanopipet was filled with aqueous HClO_4_ electrolyte, and a calibrated QRCE was placed inside the pipet at an appropriate position. The droplet meniscus at the end of the pipet was used to probe the HEO-P electrodes (working electrode).

The pipet was first mounted on an xyz-piezoelectric positioner (P-611.3S, Physik Instrumente, Germany) for micrometer maneuvering. The probe was coarsely positioned above the HEO-P electrode using stepper motors (8303 Picomotor Actuator, Newport, U.S.A.) and for fine movement piezoelectric positioner was used. Scanning of the HEO-P electrode was carried out hopping mode, where each hop consists of retract, XY movement, and approach, followed by electrochemical scanning. During the approach, the surface feedback current (*i*_surf_) from the HEO-P surface was used to detect when the droplet meniscus had made contact with the HEO-P surface. Note that the nanopipet itself did not physically come into contact with the substrate.

The current from the HEO-P electrodes was measured with a patch-clamp amplifier Dagan Chem-clamp. Data were recorded every 4 μs, averaged 256 times, to yield a data acquisition rate of 4 × (256 + 1) = 1028 μs per point. Warwick Electrochemical Scanning Probe Microscopy software (WEC-SPM, www.warwick.ac.uk/electrochemistry) was utilized to achieve fast data acquisition and fine control of the instrument, where an FPGA card (PCIe-7852R, National Instruments) was used and controlled by a LabVIEW 2024 (National Instruments) interface. The entire SECCM setup was placed in a faraday cage equipped with heat sinks and vacuum panels to minimize thermal drift and noise. The raw data from SECCM were processed using the MATLAB R2024a (MathWorks, Inc., USA.) software package. Data plotting was carried out using the OriginPro 2025b (OriginLab, USA.) software package.

Ag/AgCl was chosen as QRCE because it is commonly used for its excellent stability in acid electrolytes.^54^ The QRCE was prepared from a Ag wire (0.25 mm diameter, Goodfellow, U.K., 99.99%) (Section S1). The potential of this electrode was routinely calibrated by measuring the open-circuit potential, OCP, against a saturated calomel electrode (SCE, Saturated KCl) before and after the SECCM experiment. The QRCE potential can be converted to the reversible hydrogen electrode (RHE) scale using the following equation:^55^

5 $$E_{\mathrm{vs}\;\mathrm{RHE}}=E_{\mathrm{vs}\;\mathrm{QRCE}}+E_{\mathrm{SCE}\left(\mathrm{sat}.\;\mathrm{KCl}\right)}^{0}+{\mathrm{OCP}}_{\mathrm{QRCE}}+0.059\times \mathrm{pH}$$

where *E*_vs QRCE_ is the experimental potential, *E*_SCE (sat. KCl)_^0^ is the standard reduction potential of SCE, and OCP_QRCE_ is the open circuit potential of QRCE measured against commercial SCE.

### Surface Characterization

SEM imaging was done using a FEI Quanta 600F scanning electron microscope with a field emission electron source. HEO-P electrodes were mounted on aluminum stubs using double–sided carbon tape. SEM images were acquired under high–vacuum mode (∼10^–6^ Torr). No coating was needed for image acquisition. EDS elemental analysis of micro areas was done with the use of an EDAX Octane Elect X-ray detector, within the same SEM instrument. Beam energy and currents were optimized for capturing the best images and to achieve appropriate X-ray counts for EDS analysis. X-ray diffraction (XRD) measurements were done using a Rigaku SmartLab multipurpose X-ray diffractometer with Cu Kα radiation. Film thickness was determined by X-ray reflectivity (XRR) measurements using a Bruker D8 Discover system with Cu Kα radiation and LEPTOS software based on the Parratt formalism. AFM experiments were performed using a Dimension Icon microscope (Bruker, USA) using ScanAsyst Peak Force Tapping (in air) mode. ScanAsyst in Air cantilevers (tip radius: 2 nm, spring constant: 0.4 N m^–1^) were used for scanning. Pit surface area and volumes were calculated from AFM images using a triangulation-based approach in Gwyddion-software (Gwyddion 2.67).^56^ X-ray photoelectron (XPS) spectra were collected using a Kratos Axis Ultra DLD system in a UHV system equipped with a hemispherical analyzer (monochromatic Al Kα X-ray source). Sample charging was compensated using the instrument’s neutralizer. Data were analyzed using the CASA XPS software.

### Pit-Growth Model

The pit is modeled as an axisymmetric cavity whose *r* – *z* cross-section is an elliptical wall revolved about the symmetry axis (Figure 8a). At time τ, the wall is described by an ellipse centered at (*r*,*z*) = (*a*_0_,0),

6 $$\frac{{\left(r-a_{0}\right)}^{2}}{R_{r}{\left(\tau \right)}^{2}}+\frac{z^{2}}{b{\left(\tau \right)}^{2}}=1$$

with vertical semiaxis

7 $$b\left(\tau \right)=k_{d}\tau$$

and radial semiaxis

8 $$R_{r}\left(\tau \right)=\frac{b\left(\tau \right)}{\tan\theta }$$

where *a*_0_ is the initial footprint radius, θ is the wall angle relative to the top surface (0 < θ < 90°), and *k*_d_ is the single kinetic parameter fit from the data. The rim (opening) radius follows as

$$a\left(\tau \right)=a_{0}+\frac{b\left(\tau \right)}{\tan\theta }$$

The physical oxide thickness is *h*. Before the pit reaches the substrate (*b*(τ) < *h*), the visible cavity extends from *z* = 0 to *z* = *b*(τ). After the pit reaches the substrate (*b*(τ) ≥ *h*), the observed cavity cannot deepen further, but the wall continues to evolve according to the same ellipse (eq 6). The visible shape is the upper segment of the ellipse truncated by the plane *z = h*, while the wall remains tangent to a virtual base at *z* = *b*(τ) (Figure 7). This is implemented through

9 $$Z\left(\tau \right)=\min\left(b\left(\tau \right),h\right),\zeta \left(\tau \right)=\frac{Z\left(\tau \right)}{b\left(\tau \right)}\in \left[0,1\right]$$

and the contact time is

10 $$\tau_{c}=\frac{h}{k_{d}}$$

At a given depth *z*∈[0,*Z*(τ)], the radial coordinate of the wall is

11 $$r\left(z;\tau \right)=a_{0}+R_{r}\left(\tau \right)\sqrt{1-(z/b{\left(\tau \right)}^{2}}$$

Revolving this contour about the axis gives the cavity volume

12 $$V\left(\tau \right)=\pi \int_{0}^{Z\left(\tau \right)}r{\left(z;\tau \right)}^{2}dz$$

which evaluates the closed-form expression used in the fitting script:

13 $$V\left(\tau \right)=\pi \left[a_{0}^{2}Z+a_{0}R_{r}b\left(\zeta \sqrt{1-\zeta^{2}}+\arcsin\zeta \right)+R_{r}^{2}b\left(\zeta -\frac{\zeta^{3}}{3}\right)\right]$$

with *b* = *b*(τ), *R*_*r*_ = *R*_*r*_(τ), *Z* = *Z*(τ), and ζ = ζ­(τ) (eq 9). This single formula covers both regimes automatically, precontact: *b* < *h*⇒*Z* = *b*, ζ = 1, and postcontact: *b* ≥ *h* ⇒ *Z* = *h*, ζ = *h*/*b*.

Although *k*_*d*_ is extracted by fitting the volume (eq 13), the same geometry provides the exposed surface area used to interpret the current transient and the “hump” discussed in the main text. We write the exposed area as the sum of (i) the area of the horizontal “cap” at the truncation plane *z* = *Z*(τ) and (ii) the lateral wall area of the truncated ellipse segment:

14 $$A\left(\tau \right)=A_{\mathrm{cap}}\left(\tau \right)+A_{\mathrm{wall}}\left(\tau \right)$$

The cap radius at *z* = *Z* is *r*(*Z;*τ) from eq 11, so

15 $$A_{\mathrm{cap}}\left(\tau \right)=\pi r{\left(Z;t\right)}^{2}=\pi {\left[a_{0}+R_{r}\sqrt{1-\zeta^{2}}\right]}^{2}$$

The wall area follows from the surface-of-revolution formula,

16 $$A_{\mathrm{wall}}\left(\tau \right)=2\pi \int_{0}^{Z\left(\tau \right)}r\left(z;t\right)\sqrt{1+{\left(\frac{dr}{dz}\right)}^{2}}dz$$

with,

17 $$\frac{dr}{dz}=-\frac{R_{r}}{b}\frac{z/b}{\sqrt{1-{\left(\frac{z}{b}\right)}^{2}}}$$

Equations 14–17 are not required to extract *k*_d_ from *V*(τ). As illustrated in Figure 8, the transition from *Z* = *b*(τ) to *Z* = *h* at τ = τ_c_ produces a change in the growth mode (from vertical deepening to lateral widening), which is reflected in the evolution of *A*(τ) and the current hump.

### Numerical Fitting Procedure for *k*_d_ and Its Uncertainty

For each data set {τ_*i*_,*V*_*i*_}, we determine *k̂*_d_ by minimizing the sum of squared residuals,

18 $$\mathrm{SSE}\left(k_{d}\right)=\sum_{i=1}^{N}{\left[V\left(\tau_{i};k_{d}\right)-V_{i}\right]}^{2}$$

using a bounded golden-section search on *k*_d_ ∈ [*k*_d,min_, *k*_d,max_], with *V*(τ_*i*_;*k*_d_) computed from eq 13.

Standard error on *k̂*_d_. With one fitted parameter, we estimate the standard error by linearization,

19 $$\mathrm{Var}\left({\mathrm{k̂}}_{d}\right)\approx \frac{s^{2}}{J^{T}J},s^{2}=\frac{\mathrm{SSE}\left({\mathrm{k̂}}_{d}\right)}{N-1}$$

where *J*_*i*_ = ∂*V*(τ_*i*_;*k*_d_)/∂*k*_d_|_*k̂*_d__ is computed by symmetric finite differences at *k̂_d_*.

## Supplementary Material



## Notes

XRD, XRR, EDX analysis, XPS analysis, *i–t* traces for scan–hopping experiments as a function of pulse time, AFM images and analysis, Calculation for *Q*_pit_ for both HEO-P from Faraday’s law of oxidative dissolution, *i–t* traces for scan–hopping experiments as a function of OER overpotential, *Q*_pit_/*Q*_total_ plot as a function of overpotential, pH-dependent OER studies, X-ray photoelectron spectroscopy for O 1s (PDF)
