# Supplementary material for: Decoupling Dissolution from Water Oxidation at Single-Faceted High-Entropy Perovskite Electrodes
Source: J Am Chem Soc. 2026 Oct 21;148(43):46593–606. doi: 10.1021/jacs.6c09065 (PMC13741833; doi:10.1021/jacs.6c09065)
Supplement: Supplementary file 1 [file ja6c09065_si_001.pdf]

Decoupling Dissolution from Water Oxidation at Single-Faceted High-Entropy Perovskite Electrodes

Subhajit Bhunia,[1#] Esteban D. Gadea,[1#] Alessandro R. Mazza,[5] Mohana V. Kante,[2] Gangli Wang,[4*] Sheng Dai,[3*] Thomas Z. Ward,[2*],Valeria Molinero,[1*] Henry S. White[1*]

Affiliations:

[1]Department of Chemistry, University of Utah, 315 South 1400 East, Salt Lake City, Utah 84112, United States

[2]Center for Nanophase Material Sciences, Oak Ridge National Laboratory, Oak Ridge, Tennessee, 37830, United States

[3]Chemical Sciences Division, Oak Ridge National Laboratory, Oak Ridge, Tennessee 37831, United States

[4]Department of Chemistry, Georgia State University, Atlanta, GA, 30302, United States

[5]Materials Science and Technology Division, Los Alamos National Laboratory, Los Alamos, New Mexico 87545, United States

*Authors to whom correspondence should be addressed, *white@chem.utah.edu*, *Valeria.Molinero@utah.edu*, *wardtz@ornl.gov*, *dais@ornl.gov*, *glwang@gsu.edu*.

Figure S1. Schematic of HEO-P electrodes 5
Figure S2. HRXRD profiles of (a) La-HEO-P and (b) LaSr-HEO-P 5
Figure S3. X-ray reflectivity (XRR) profiles of (a) La-HEO-P and (b) LaSr-HEO-P 6
Figure S4. The AFM images of RMS roughness (*R*q) for HEO-P electrodes. 6
Figure S5. EDX analysis of a representative La-HEO-P film. 7
Figure S6. EDX analysis of a representative LaSr-HEO-P film. 8
Figure S7. XPS analysis for La-HEO-P on the film and LaSr-HEO-P from various location. ... 9
Figure S8. La3d and Ni2p XPS spectra for HEO-Ps. 9
Figure S9. Co2p XPS spectra for HEO-Ps. 10
Figure S10. Fe2p XPS spectra for HEO-Ps. 10
Figure S11. Mn2p XPS spectra for HEO-Ps. 11
Figure S12. Cr2p XPS spectra for HEO-Ps. 11
Figure S13. OCP vs time plot for QRCEs 12
Figure S14. *i– t* traces from single pipette landing at 1.72 V on La-HEO-P-89 nm and LaSr-HEO-P-29 nm electrodes. 13
Figure S15. *i–t* traces from each scan-hopping on La-HEO-P-89 nm. 15
Figure S16. *i–t* traces from each scan-hopping on LaSr-HEO-P-29 nm. 15
Figure S17. *Ex-situ* AFM characterization of La-HEO-P-89 nm. 16
Figure S18. *Ex-situ* AFM characterization of LaSr-HEO-P-29 nm. 17
Figure S19. EDS spectra analysis for time-dependent *i–t* hopping experiments on La-HEO-P-89 nm. 18
Figure S20. AFM image analysis for OER reaction area on La-HEO-P-89 nm. 19
Figure S21. Plot for Surface area of the dissolution pits as a function of τ for La-HEO-P-89 nm and for LaSr-HEO-P-29 nm. 28
Figure S22. Observed changes to cation charge state population relative to *A*-site doping.... 28
Figure S23. Faradaic efficiency of OER for HEO-P electrodes 31
Figure S24. *i–t* traces, *ex-situ* AFM, and plot for Q_{total} and Q_{pit} as a function of τ for La-HEO-P-29 nm 32
Figure S25. *i–t* traces, *ex-situ* AFM, and plot for Q_{total} and Q_{pit} as a function of τ for LaSr-HEO-P-100 nm. 33
Figure S26. Analysis of pre-parforation and post-perforation regime for La-HEO-P-29 nm . 34
Figure S27. Analysis of pre-parforation and post-perforation regime for LaSr-HEO-P-100 nm. 34

Figure S28. i–t traces collected from each scan-hopping experiment as a function of η for La-HEO-P-89 nm. 36
Figure S29. i–t traces collected from each scan-hopping experiment as a function of η for LaSr-HEO-P-29 nm. 36
Figure S30. i–t traces collected from each scan-hopping experiment as a function of η for La-HEO-P-29 nm.. 37
Figure S31. i–t traces collected from each scan-hopping experiment as a function of η for LaSr-HEO-P-100 nm 37
Figure S32. Representative SEM image of the i–t hopping experiments as a function of η on the La-HEO-P-89 nm electrode. 38
Figure S33. Representative SEM image of the i–t hopping experiments as a function of η at different overpotentials on LaSr-HEO-P-29 nm electrode 38
Figure S34. AFM image of the area where i–t hopping experiments as a function of η were performed on La-HEO-P-89 nm electrode. 39
Figure S35. AFM image of the area where i–t hopping experiments as a function of η were performed on LaSr-HEO-P-29 nm electrode. 40
Figure S36. AFM image of the area where i–t hopping experiments as a function of η were performed on La-HEO-P-29 nm electrode. 41
Figure S37. AFM image of the area where i–t hopping experiments as a function of η were performed on LaSr-HEO-P-100 nm electrode. 42
Figure S38. Representative plot for pit depth attenuation as a function of η for La-HEO-P-89nm and LaSr-HEO-P-29 nm. 43
Figure S39. Q_{total} as a function of τ at different η and Violin plot of $Q_{\mathrm{pit}}/Q_{\mathrm{total}}$ as a function of η for La-HEO-P-89 nm 43
Figure S40. Q_{total} as a function of τ at different η and Violin plot of $Q_{\mathrm{pit}}/Q_{\mathrm{total}}$ as a function of η for LaSr-HEO-P-29 nm.. 44
Figure S41. Q_{total} as a function of τ at different η and Violin plot of $Q_{\mathrm{pit}}/Q_{\mathrm{total}}$ as a function of η for La-HEO-P-29 nm. 44
Figure S42. Q_{total} as a function of τ at different η and Violin plot of $Q_{\mathrm{pit}}/Q_{\mathrm{total}}$ as a function of η for LaSr-HEO-P-100 nm.. 45
Figure S43. AFM image of the area where i–t hopping experiments was done using 25 mM $HClO_4$ filled pipette on La-HEO-P-29 nm and LaSr-HEO-P-100 nm 46
Figure S44. Volume loss profile using 25 mM $HClO_4$ electrolyte filled pipette for La-HEO-P-29 nm and LaSr-HEO-P-100 nm 46

Figure S45. pH dependent CV plot for La-HEO-P-89nm 47
Figure S46. Proton reaction order and V vs pH plot at fixed current density for La-HEO-P-89 nm. 47
Figure S47. pH dependent CV plot for LaSr-HEO-P-29 nm 48
Figure S48. Proton reaction order and V vs pH plot at fixed current density for LaSr-HEO-P-29nm. 48
Figure S49. O1s XPS spectra for La-HEO-P-89 nm and LaSr-HEO-P-29 nm.......................... 49
Section S1. Characterization of Ag/AgCl QRCE 12
Section S2. Experimental details of hop scanning with *i-t* on HEO-P electrodes as a function of τ 14
Section S3. AFM image analysis.. 19
Section S4. Conversion of pit volume to mass and charge 29-31
Section S5 Thickness dependence study for both HEO-P electrode 32-33
Section S6. Experimental details of hop scanning with *i-t* on HEO-P electrode as a function of η... 38
Section S7. Experimental details of hop scanning with *i-t* at different pH 46
Table S1. Parameters for the scan-hopping experiments as a function of τ............................ 14
Table S2. AFM analysis results as a function of pulse time for La-HEO-P-89nm. 20-24
Table S3. AFM analysis results as a function of pulse time for LaSr-HEO-P-29nm. 24-27
Table S4. Change of Oxidation states for HEO-P compositions 30
Table S5. Parameters for the scan-hopping experiments as a function of η 35
Table S6. Binding energy (B.E.) of various components of O1s spectra of HEO-P electrodes 49
References. 50

HEO-P electrode	*h* / nm
La-HEO-P-89 nm	~ 89 nm
La-HEO-P-29 nm	~ 29 nm
LaSr-HEO-P-100 nm	~ 100 nm
LaSr-HEO-P-29 nm	~ 29 nm

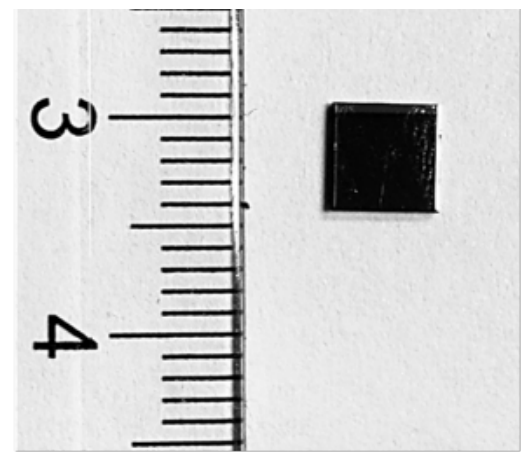

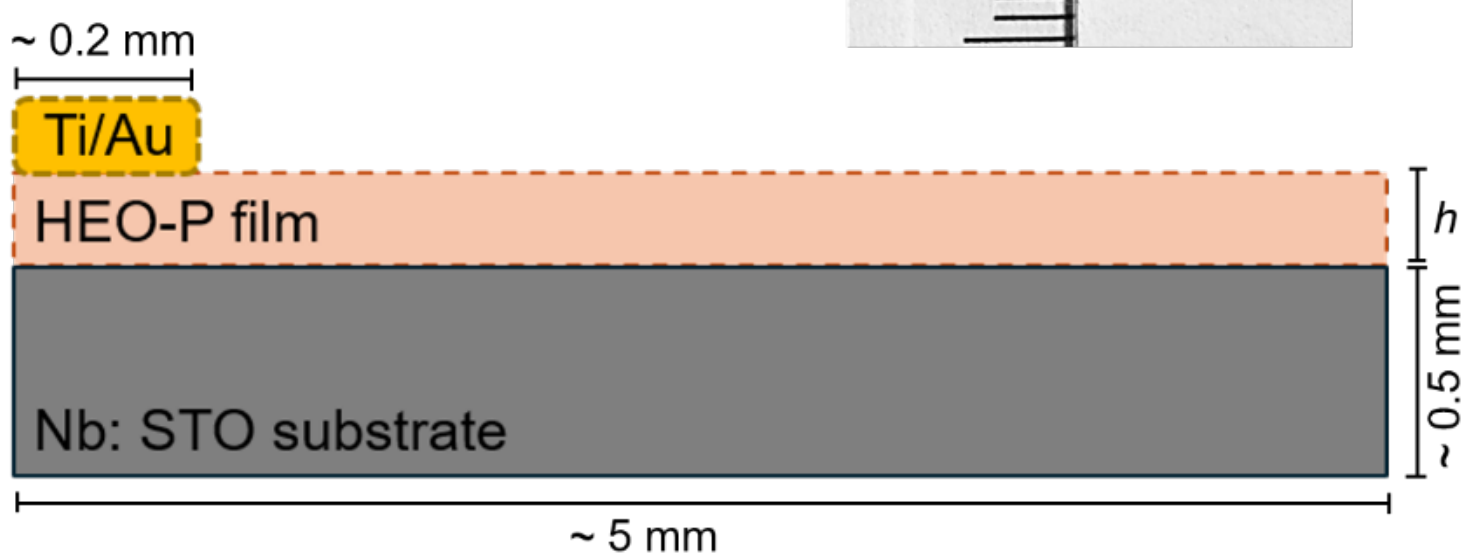

Figure S1. Schematic of HEO-P electrodes on Nb: STO substrate and a representative image of the electrode are given at the inset (right).

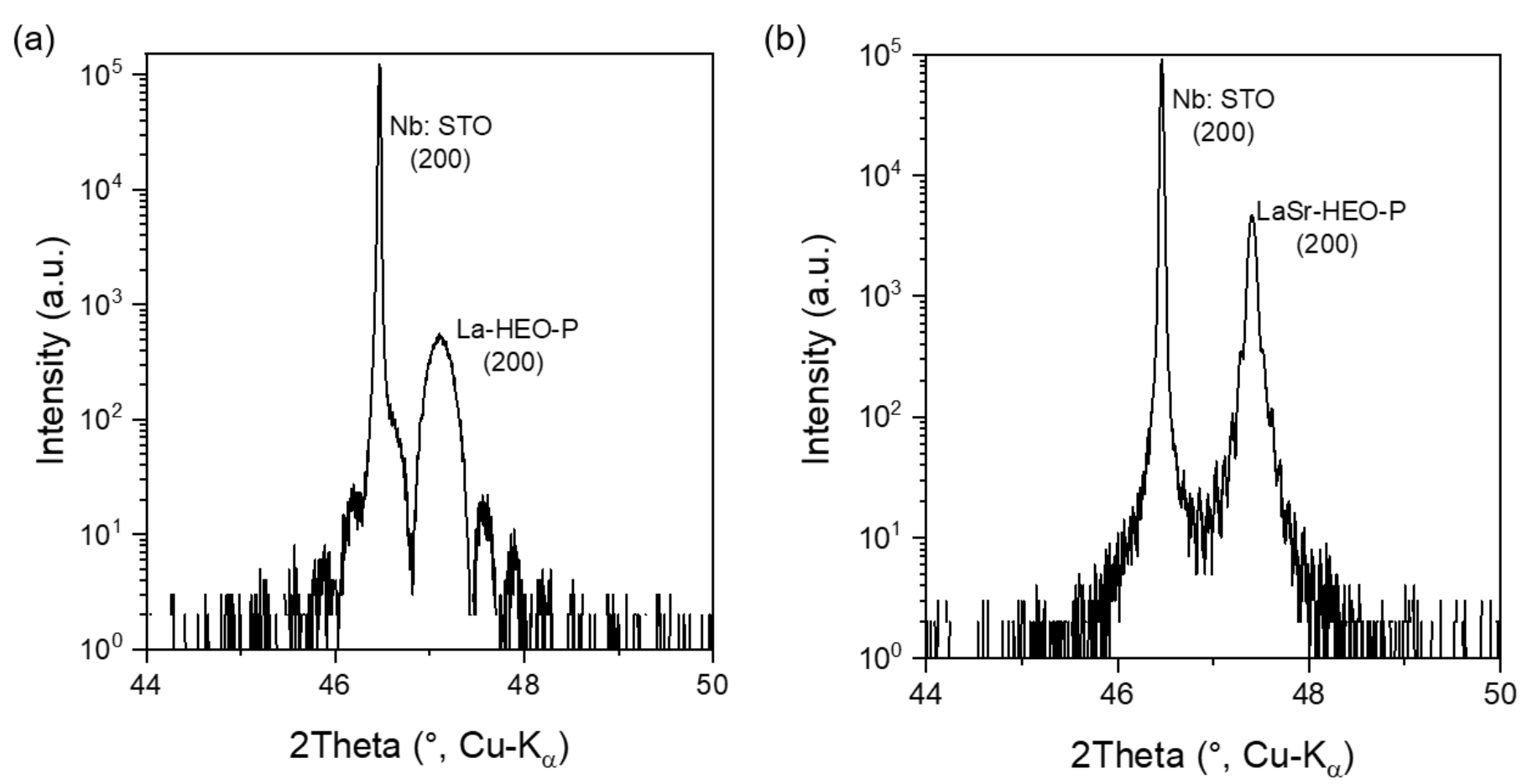

Figure S2. (a) HRXRD patterns of the La-HEO-P, and (b) LaSr-HEO-P indicating epitaxial growth of the HEO-P thin film in the (001) orientation of the perovskite structure.

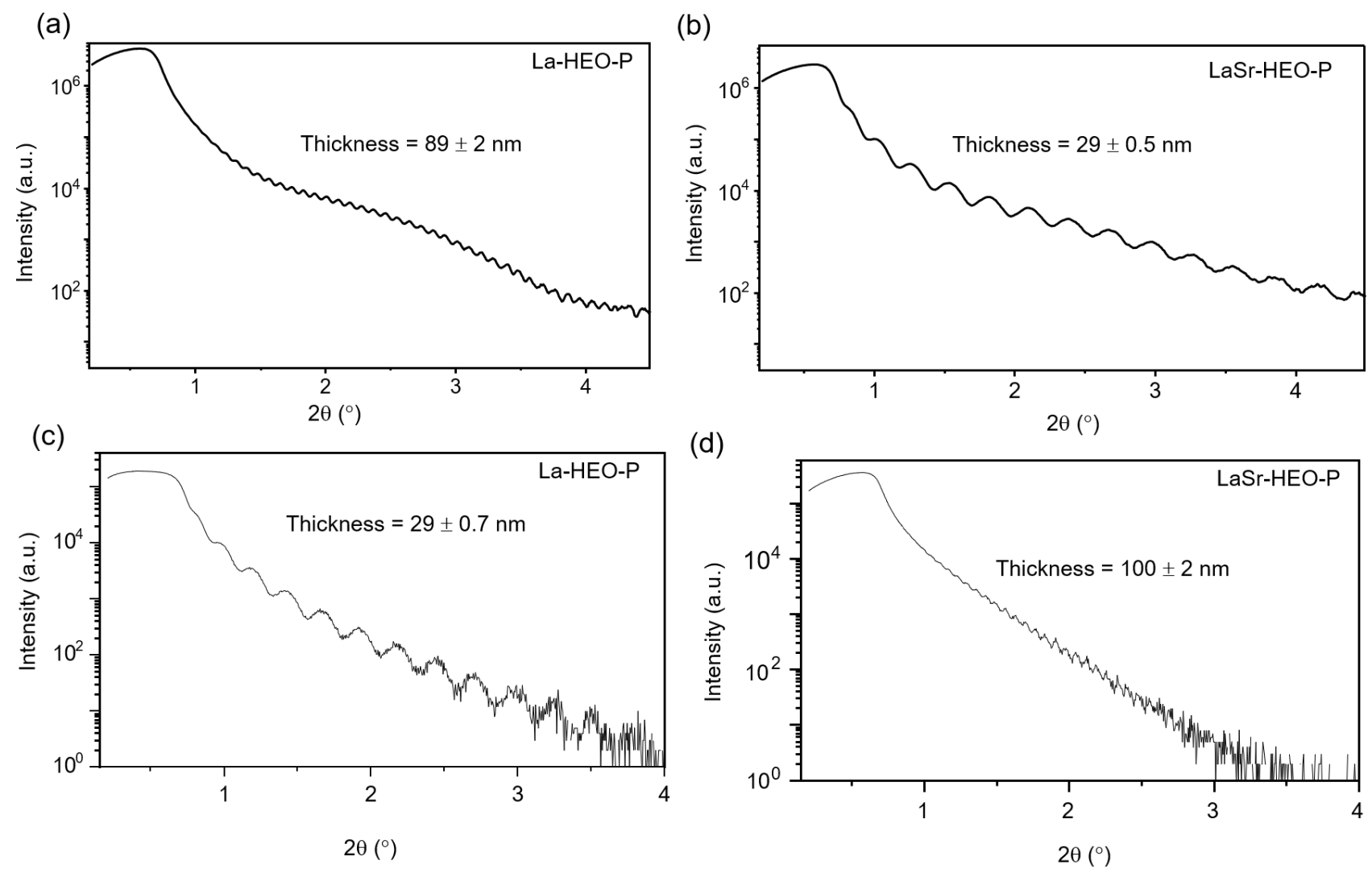

Figure S3. X-ray reflectivity (XRR) profiles of (a) La-HEO-P-89 nm; (b) LaSr-HEO-P-29 nm ; (c) La-HEO-P-29 nm and (d) LaSr-HEO-P-100 nm.

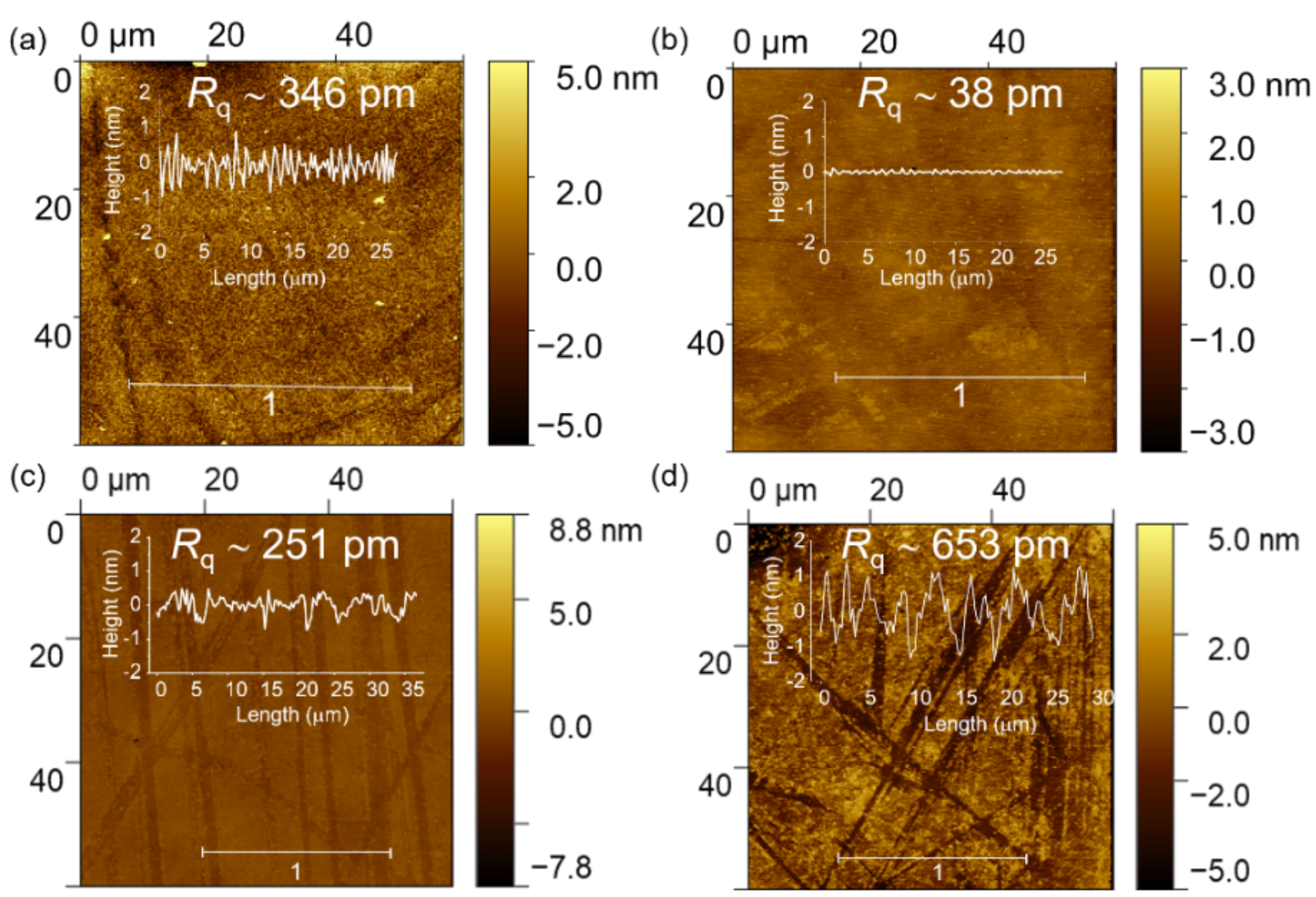

Figure S4. The AFM images of RMS roughness (*R*q) for (a) La-HEO-P-89 nm and (b) LaSr-HEO-P-29 nm electrode; (c) La-HEO-P-29 nm; (d) LaSr-HEO-P-100 nm.

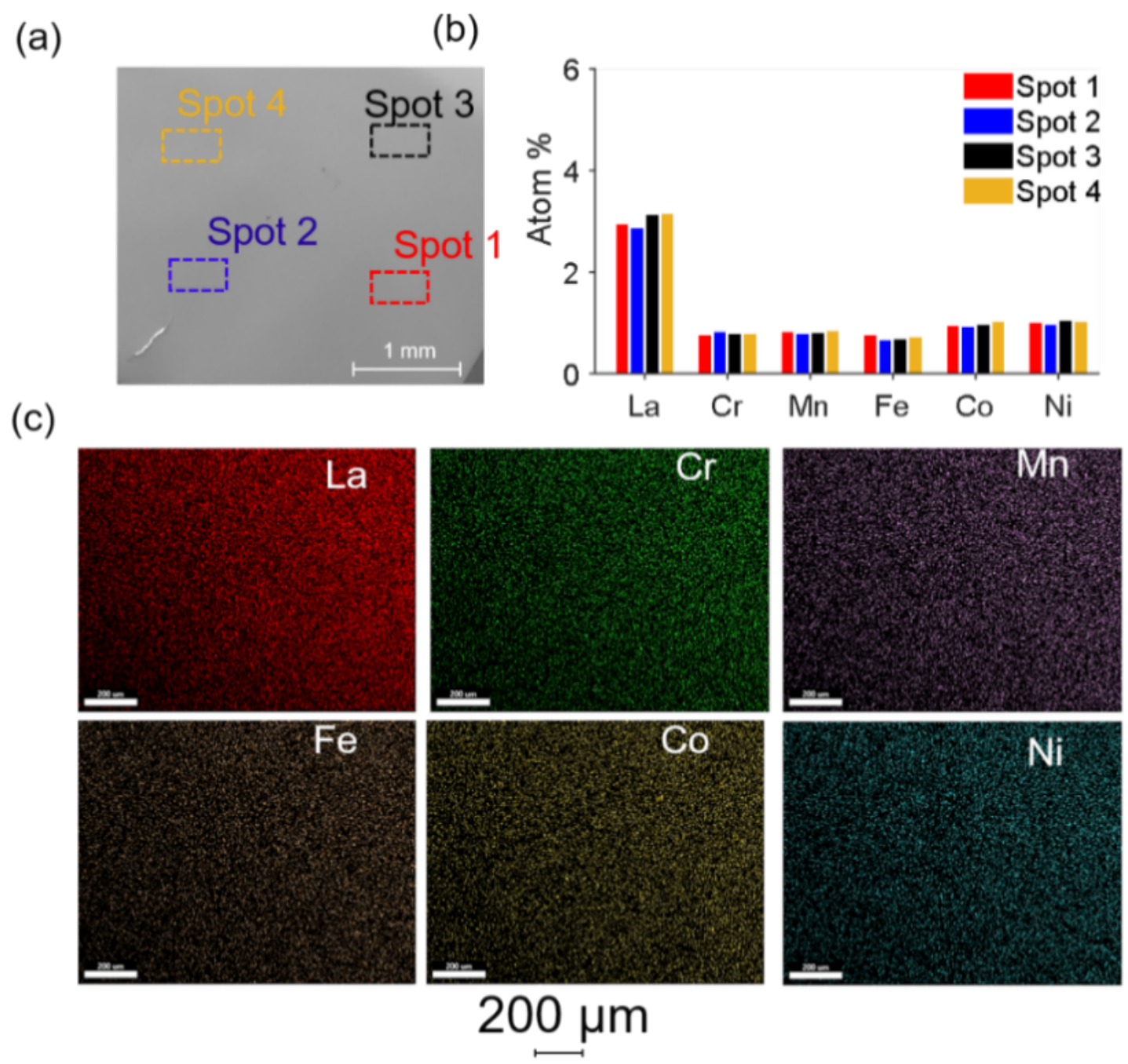

Figure S5. Compositional homogeneity of a representative La-HEO-P

(a) SEM image of La-HEO-P-29 nm where four (red, blue, yellow and black) rectangular areas are the locations selected for EDS analysis. (b) Quantitative EDS analysis acquired from the four locations demonstrates excellent agreement in the atomic fractions of La, Cr, Mn, Fe, Co, and Ni, confirming negligible compositional variation across the film. The Sr and O images were excluded from the quantitative analysis because of the contribution from the underlying Nb: STO substrate. (b) Representative SEM–EDS elemental maps of La, Cr, Mn, Fe, Co, Ni, and O collected over a 500 µm × 500 µm area.

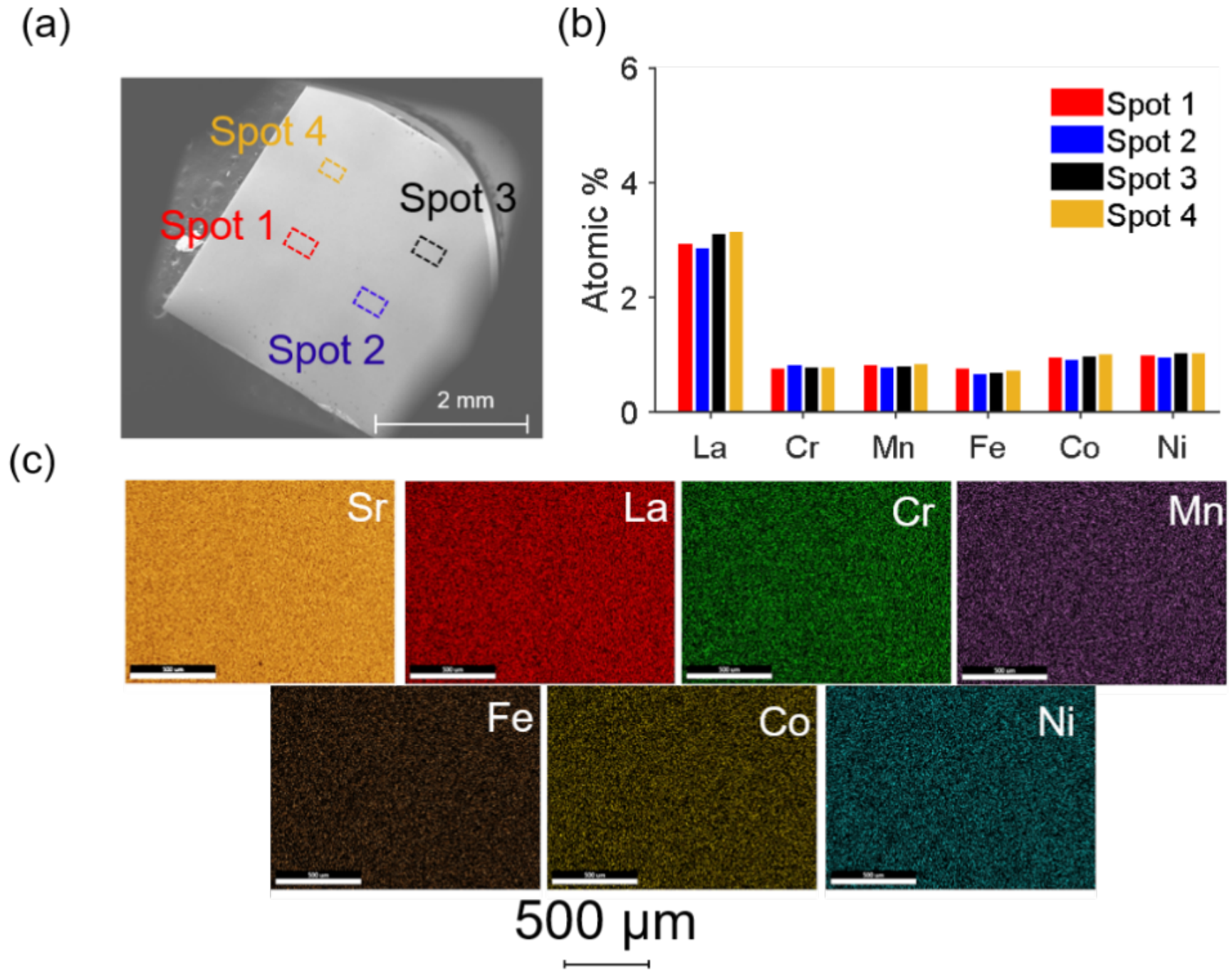

Figure S6. Compositional homogeneity of a representative LaSr-HEO-P

(a) SEM image of La-HEO-P-29 nm where four (red, blue, yellow and black) rectangular areas are the locations selected for EDS analysis. (b) Quantitative EDS analysis acquired from the four locations demonstrates excellent agreement in the atomic fractions of La, Cr, Mn, Fe, Co, and Ni, confirming negligible compositional variation across the film. The Sr and O images were excluded from the quantitative analysis because of the contribution from the underlying Nb: STO substrate. (b) Representative SEM–EDS elemental maps of La, Cr, Mn, Fe, Co, Ni, and O collected over a 500 µm × 500 µm area.

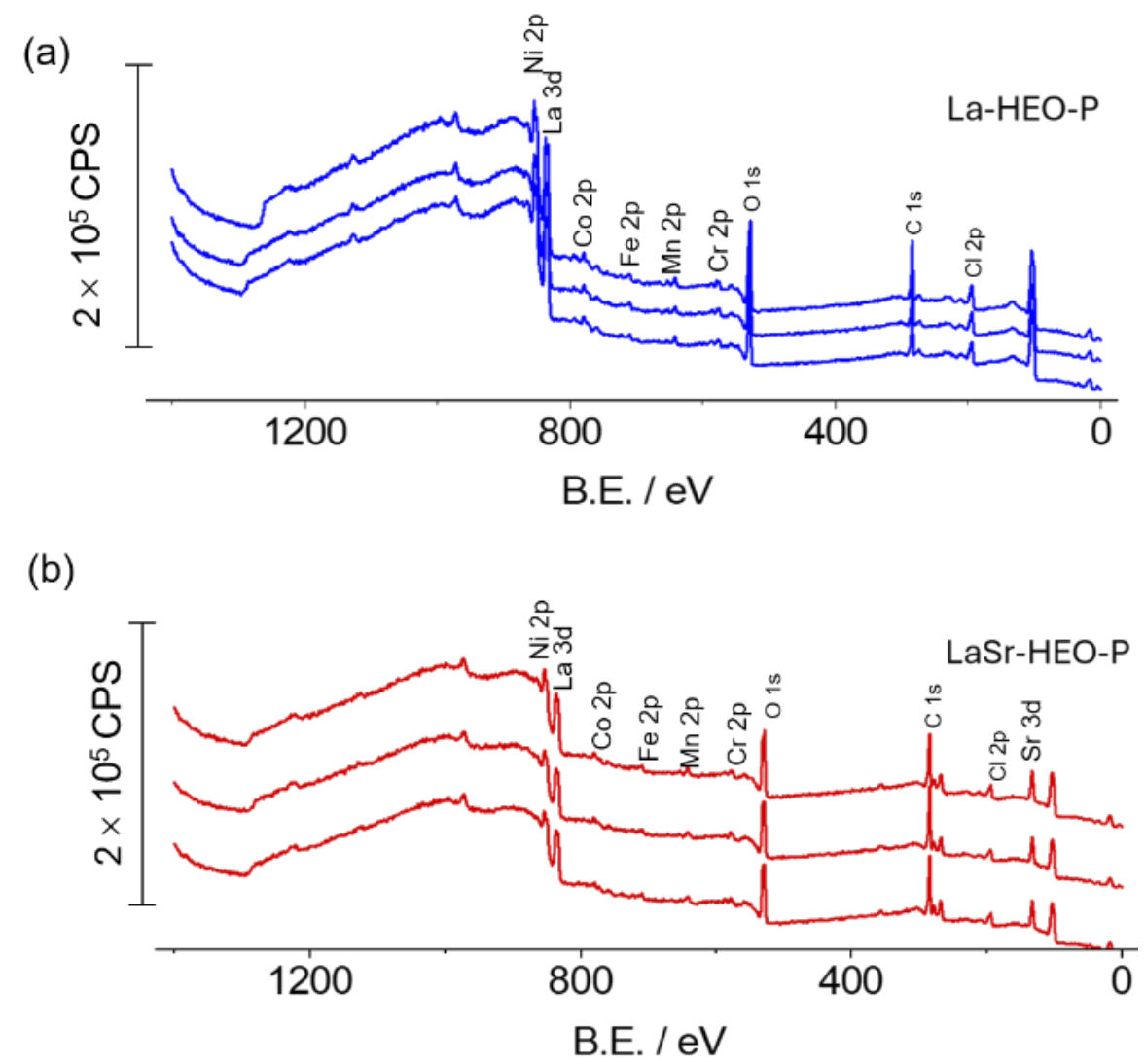

Figure S7. (a) XPS survey for La-HEO-P on the film and for (b) LaSr-HEO-P from three different location.

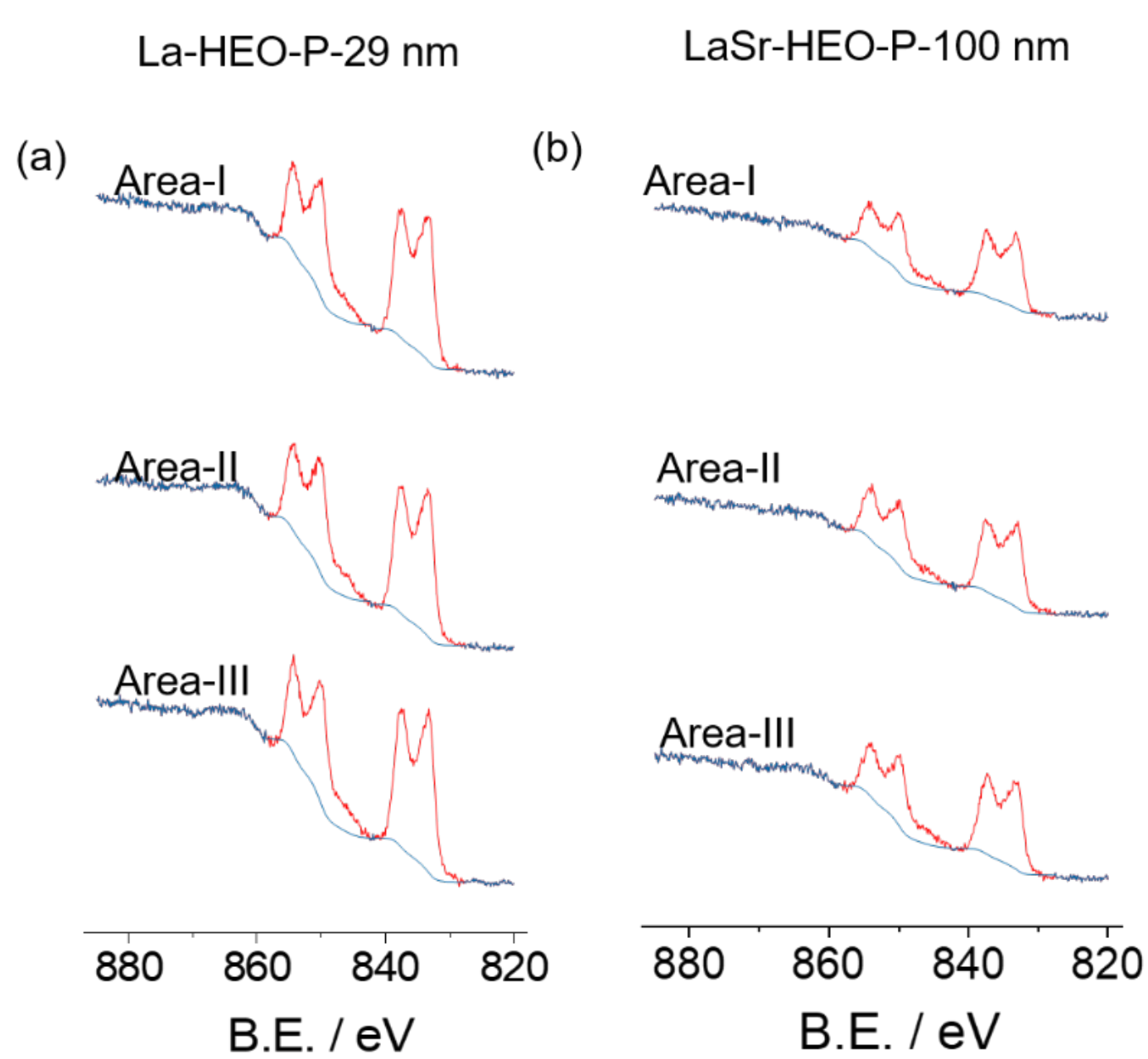

Figure S8. (a) Representative La3d and Ni2p XPS spectra for La-HEO-P on the film and for (b) LaSr-HEO-P from three different locations.

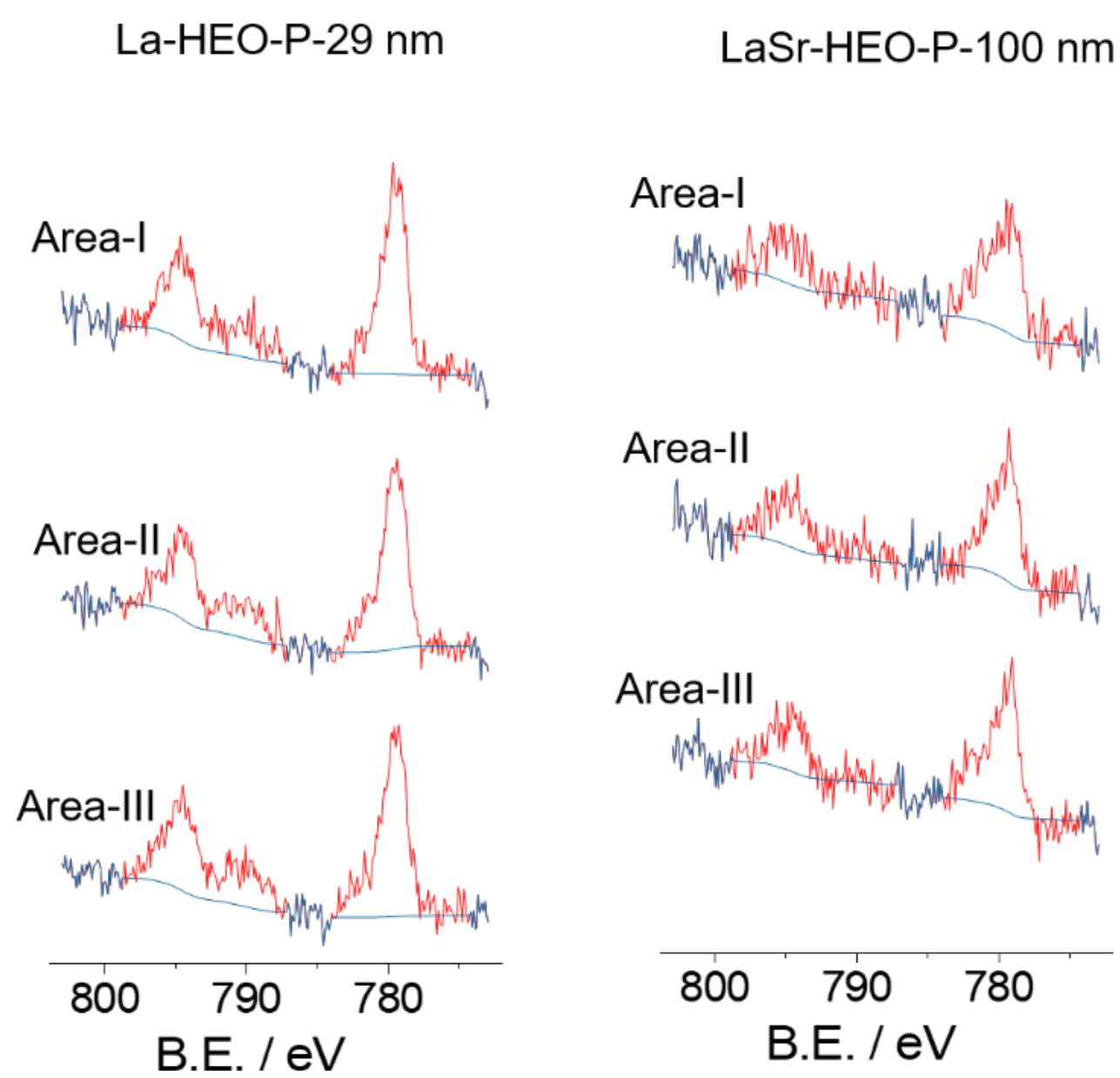

Figure S9. (a) Representative Co2p XPS spectra for La-HEO-P and for (b) LaSr-HEO-P from three different locations.

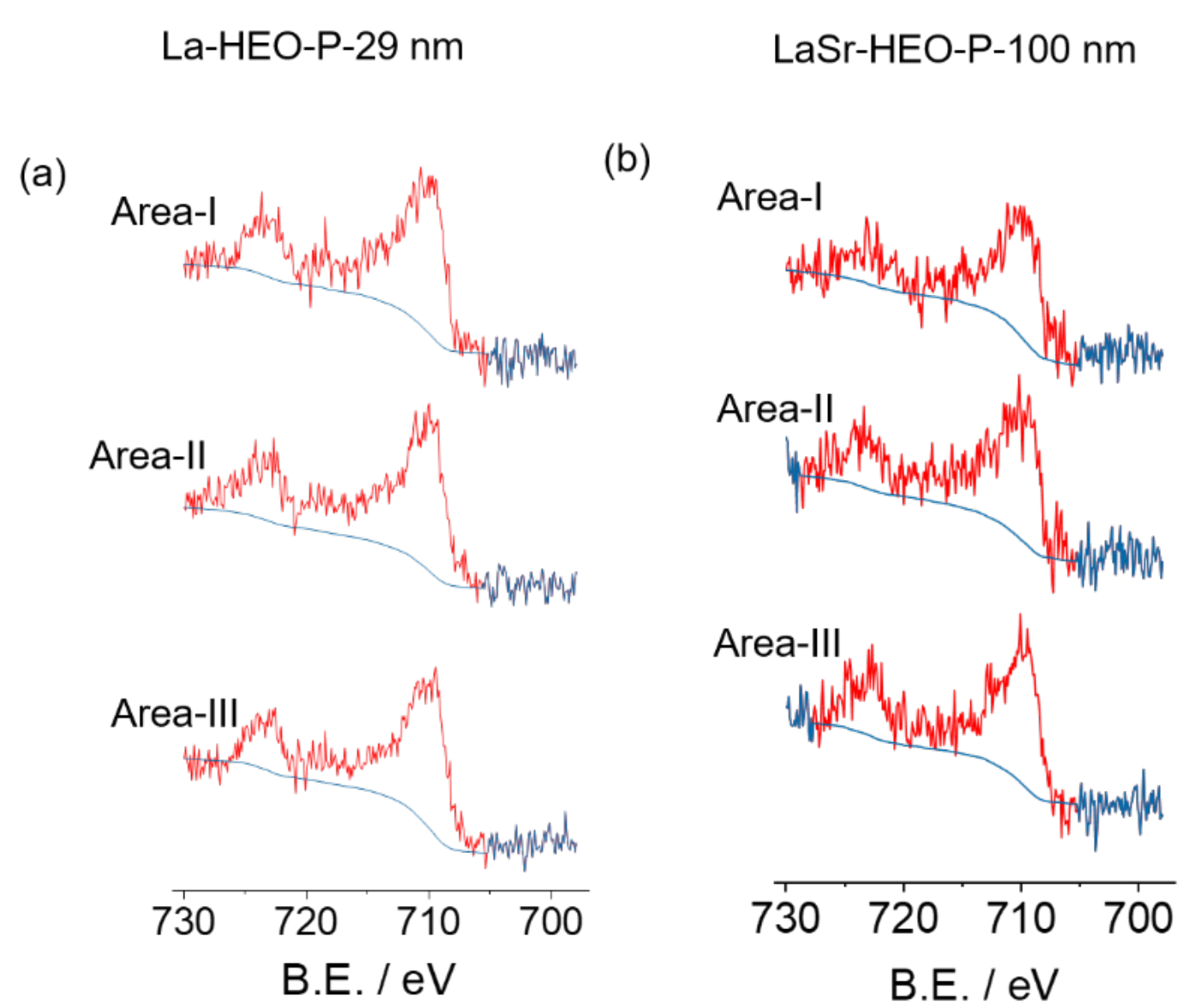

Figure S10. (a) Representative Fe2p XPS spectra for La-HEO-P and for (b) LaSr-HEO-P from three different locations.

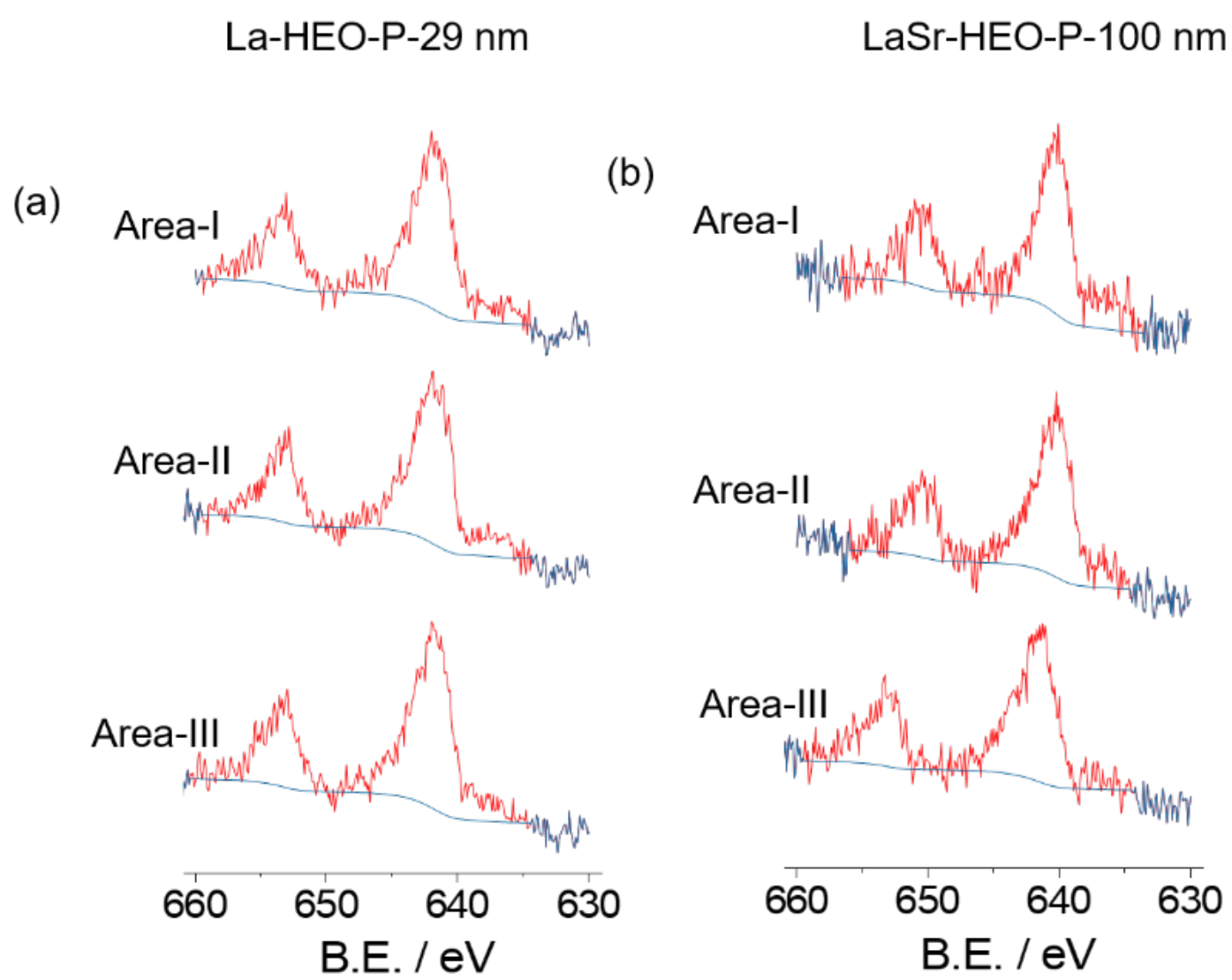

Figure S11. (a) Representative Mn2p XPS spectra for La-HEO-P and for (b) LaSr-HEO-P from three different locations.

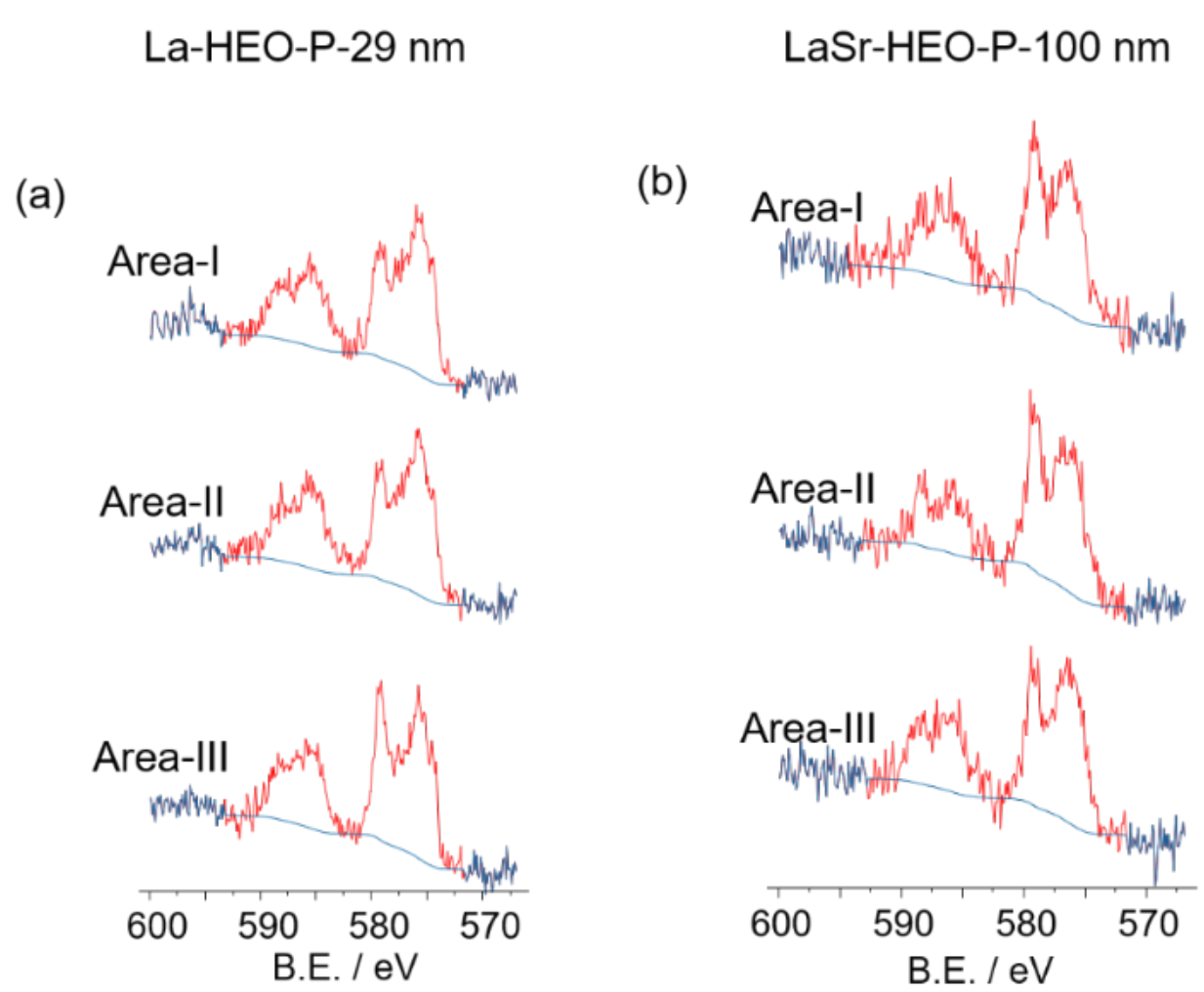

Figure S12. (a) Representative Cr2p XPS spectra for La-HEO-P and for (b) LaSr-HEO-P from three different locations.

Section S1. Characterization of Ag/AgCl QRCE

Ag/AgCl quasi-reference counter electrodes (QRCEs) were freshly prepared prior to each experiment from commercial Ag wire. The wires were mechanically polished using P400 and P1200 sandpapers (Buehler, USA), followed by immersion in 0.2 M $FeCl_3$ solution for 30 min to form a surface AgCl layer. After removal from the $FeCl_3$ solution, the wire tips were lightly polished and thoroughly rinsed with ultrapure water to ensure complete removal of residual $FeCl_3$.The potential of the QRCE was calibrated by measuring the open-circuit potential (OCP) against a Hg/Hg_2Cl_2 (sat. KCl) reference electrode before each SECCM measurement. For calibration, the QRCE was placed inside a pipette filled with the same electrolyte used for SECCM, and the pipette was immersed in a bulk electrolyte bath containing an identical solution along with the commercial Hg/Hg_2Cl_2 (sat. KCl) electrode to record the OCP-*t* trace. The OCP-*t* traces show the QRCE potential is stable after ~ 500 s and, is pH independent.

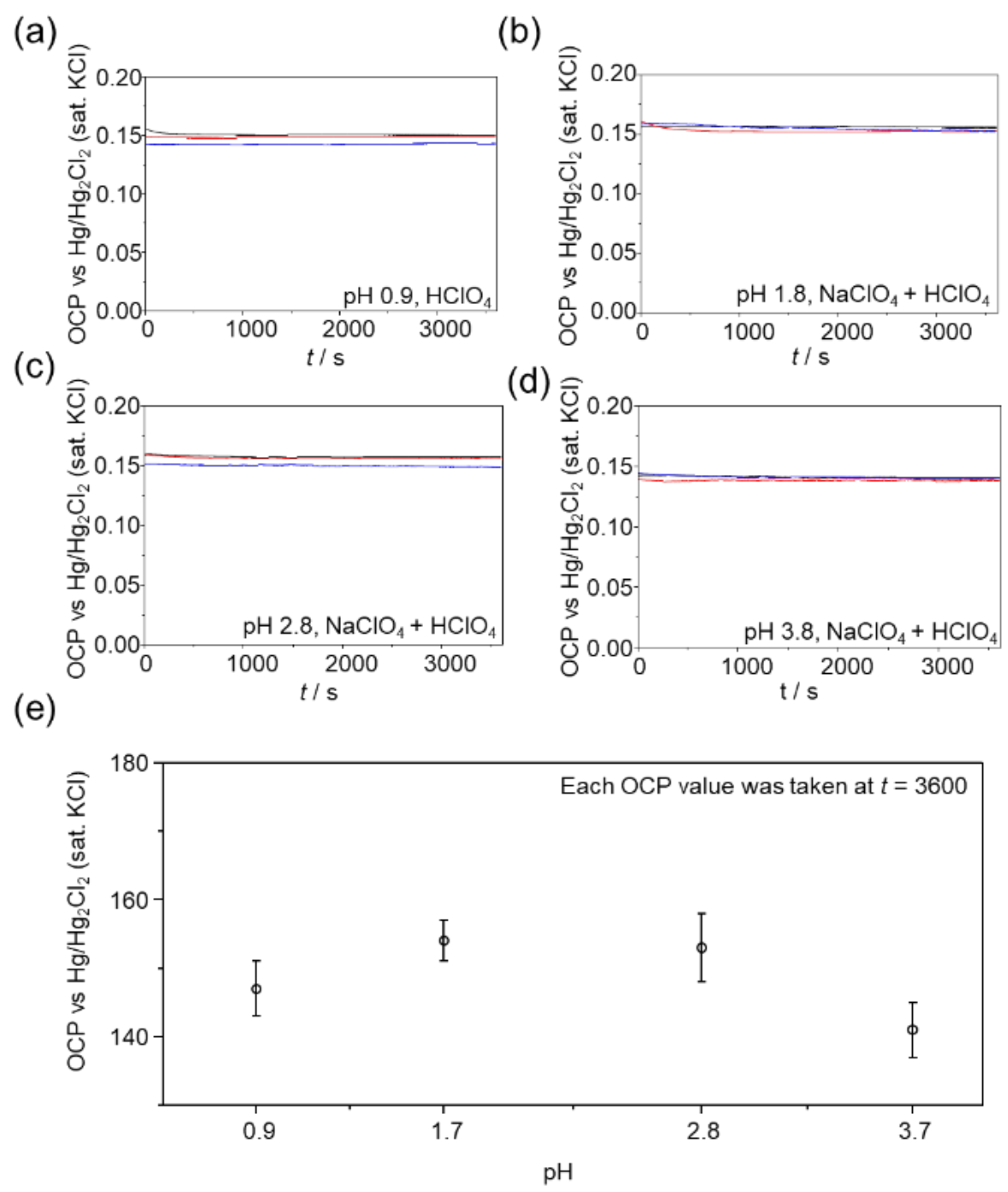

Figure S13. OCP vs *t* plot for QRCEs from pH 0.9 to 3.8, where each pH experiment was carried out three times using three different freshly prepared QRCEs to check the reproducibility (a-d); (e) OCP as a function of pH, where standard deviation was derived from three independent measurement using three QRCE

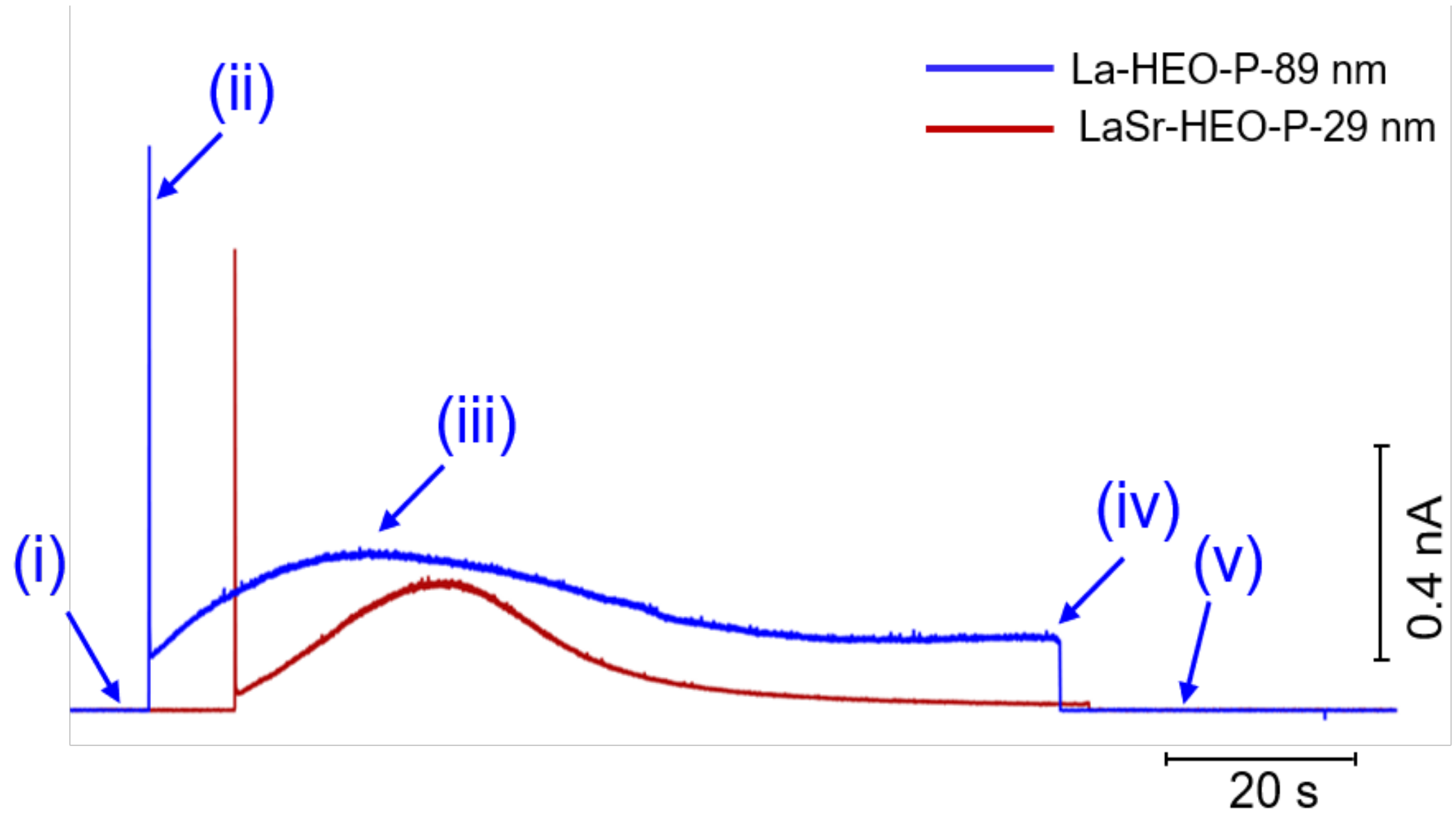

Figure S14. *i*– *t* traces from single landing (at 1.72 V) on La-HEO-P-89 nm and LaSr-HEO-P-29nm electrode. The whole trace is composed of five consecutive events, (i) approach; (ii) landing; (iii) *i*–*t* recording; (iv) retract and (v) move to the next spot. In the above plot, the current follows the baseline during approach followed by a current spike (i_{surf}), which is typical feedback from the surface upon meniscus landing. After the spike, the *i*–*t* trace is recorded until the tip is retracted. The pipette is moved to next spot and the process repeated.

Section S2. Experimental details of hop scanning with *i-t* on HEO-P electrodes as a function of τ

The *i*–*t* traces were recorded using a single nanopipette with a radius of approximately 800 nm. Before the scan-hopping experiments, the QRCE was activated by anodizing in a saturated KCl solution. The approach voltage and feedback threshold current were optimized to minimize false landing events during measurements. Lower threshold values resulted in false landings, whereas higher values caused early perturbation of the pristine HEO-P surface, occurring on timescales shorter than those relevant to the experiment. The scan-hopping experiments were conducted using the parameters summarized in Table S1.

Table S1. Parameters for the scan-hopping experiments

Parameters	Value
Voltage to working electrode (vs RHE)	~ 475 mV vs E^0_{OER}
Feedback threshold	13 pA
Hopping distance	6 μm
Retract rate	1 μm/s
Approach rate	0.15 μm/s
Scan area	36 × 36 μm^2
Sampling resolution	1028 μs

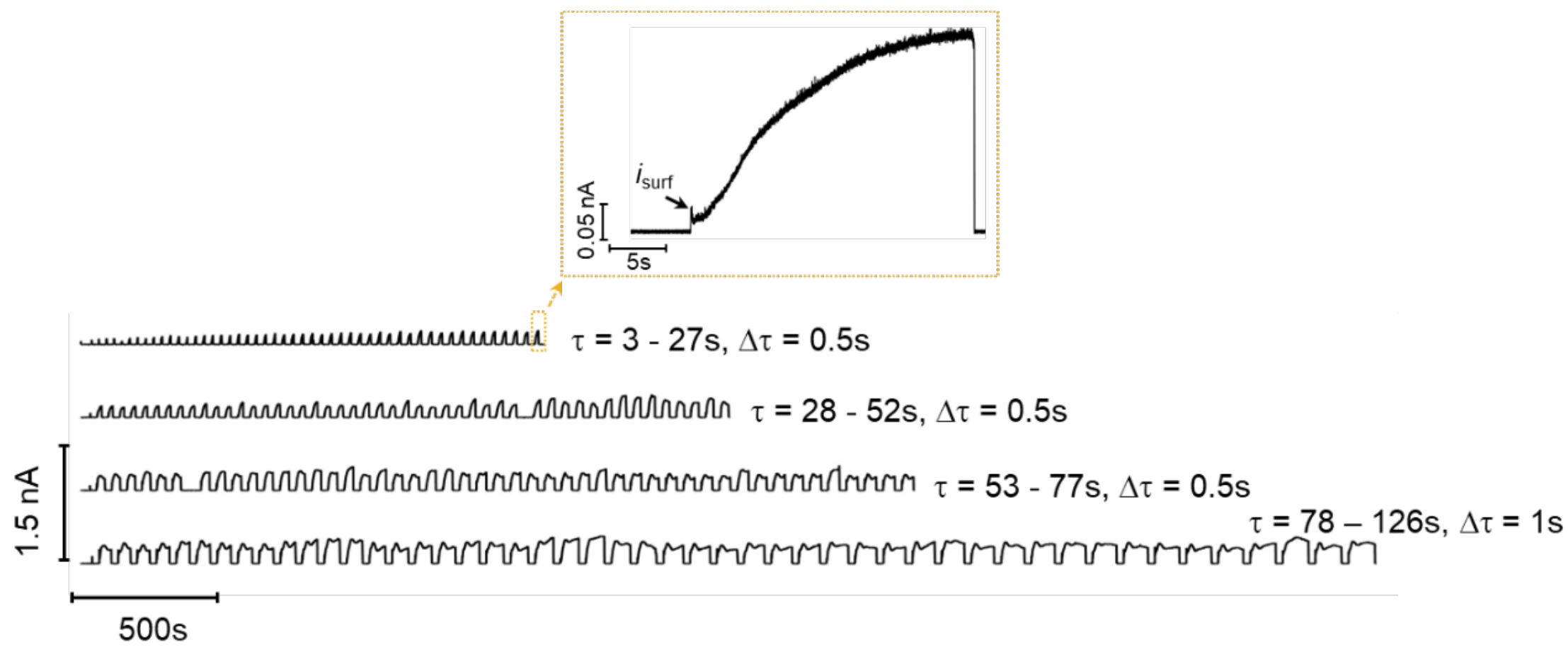

Figure S15. *i–t* traces from each scan-hopping on La-HEO-P-89 nm. The traces are arranged from top to bottom, where each scan-hopping sequence consists of 49 *i–t* measurements recorded from the initial pulse time τ to $\tau + (48 \times \Delta\tau)$ at constant overpotential. Missing *i–t* traces correspond to false-approach events (no meniscus contact with surface).

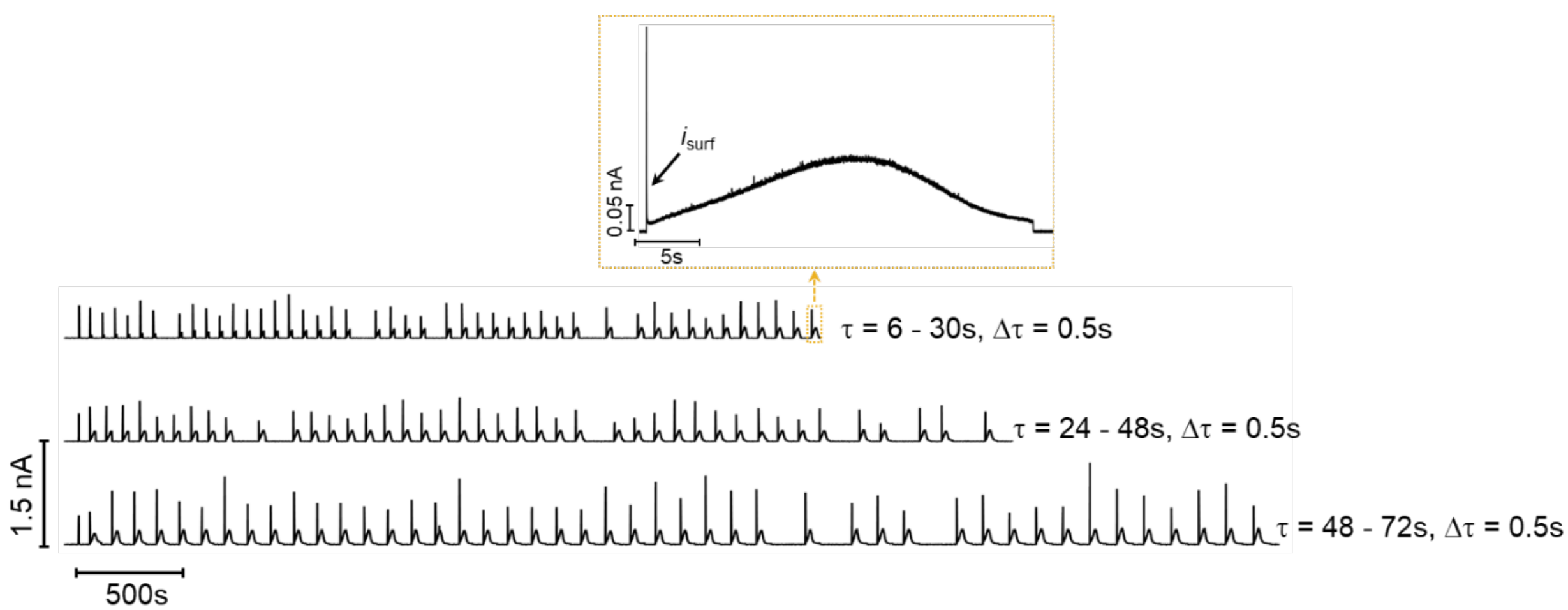

Figure S16. *i–t* traces from scan-hopping experiments on LaSr-HEO-P-29 nm. The traces are arranged from top to bottom, where each scan-hopping sequence consists of 49 *i–t* measurements recorded from the initial pulse time τ to $\tau + (48 \times \Delta\tau)$. Missing *i–t* traces correspond to false-approach events.

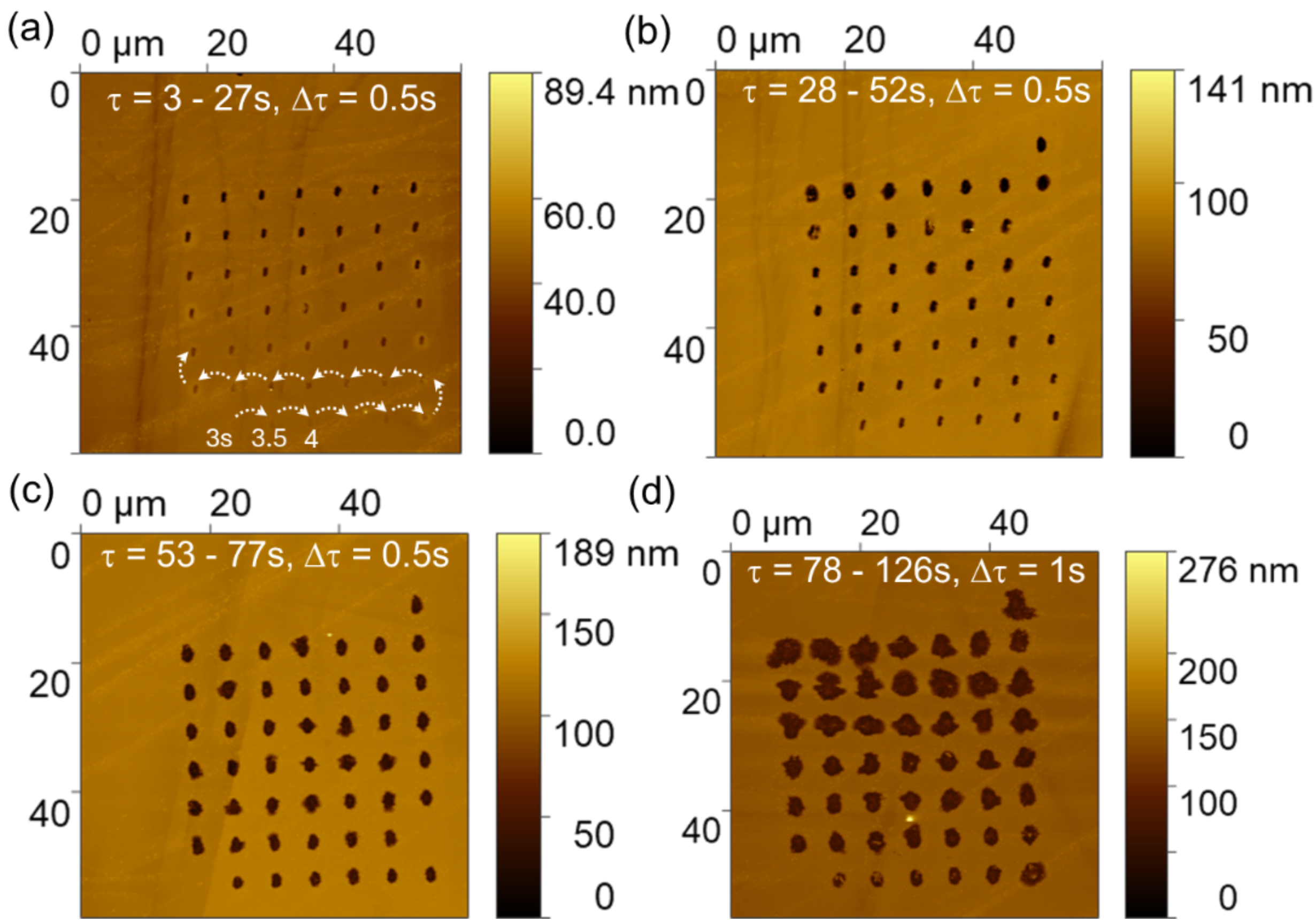

Figure S17. *Ex-situ* AFM characterization of La-HEO-P-89 nm electrode after scan-hopping experiment within the pulse time window τ = 3–126 s (a, d). The white arrows indicate the hopping trajectory of the nanopipette.

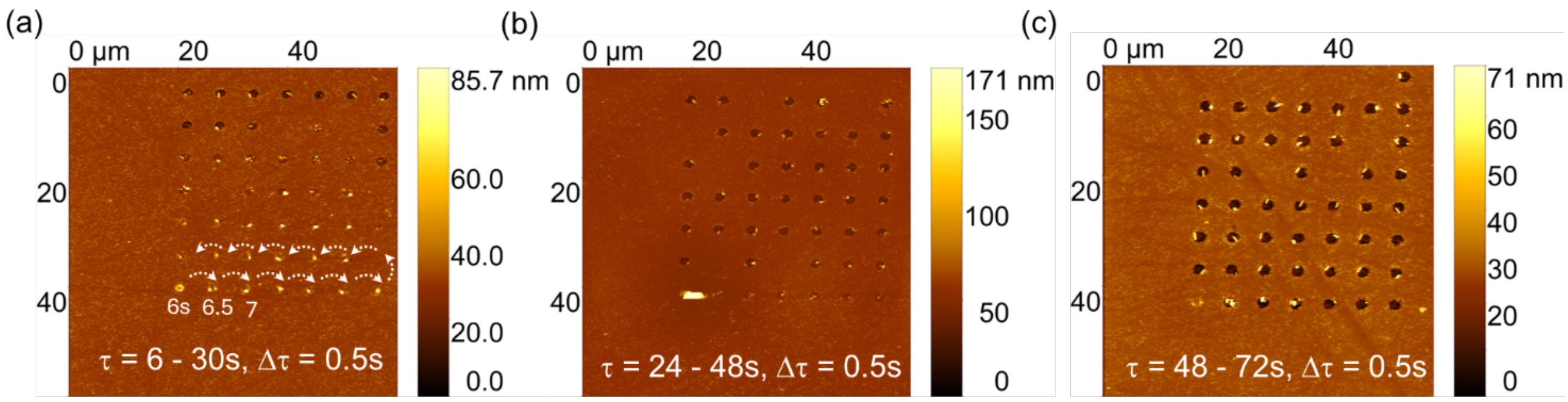

Figure S18. *Ex-situ* AFM characterization of LaSr-HEO-P-29 nm electrode after scan-hopping experiment within the pulse time window $\tau = 6–72$ s (a, c). The white arrows indicate the hopping trajectory of the nanopipette.

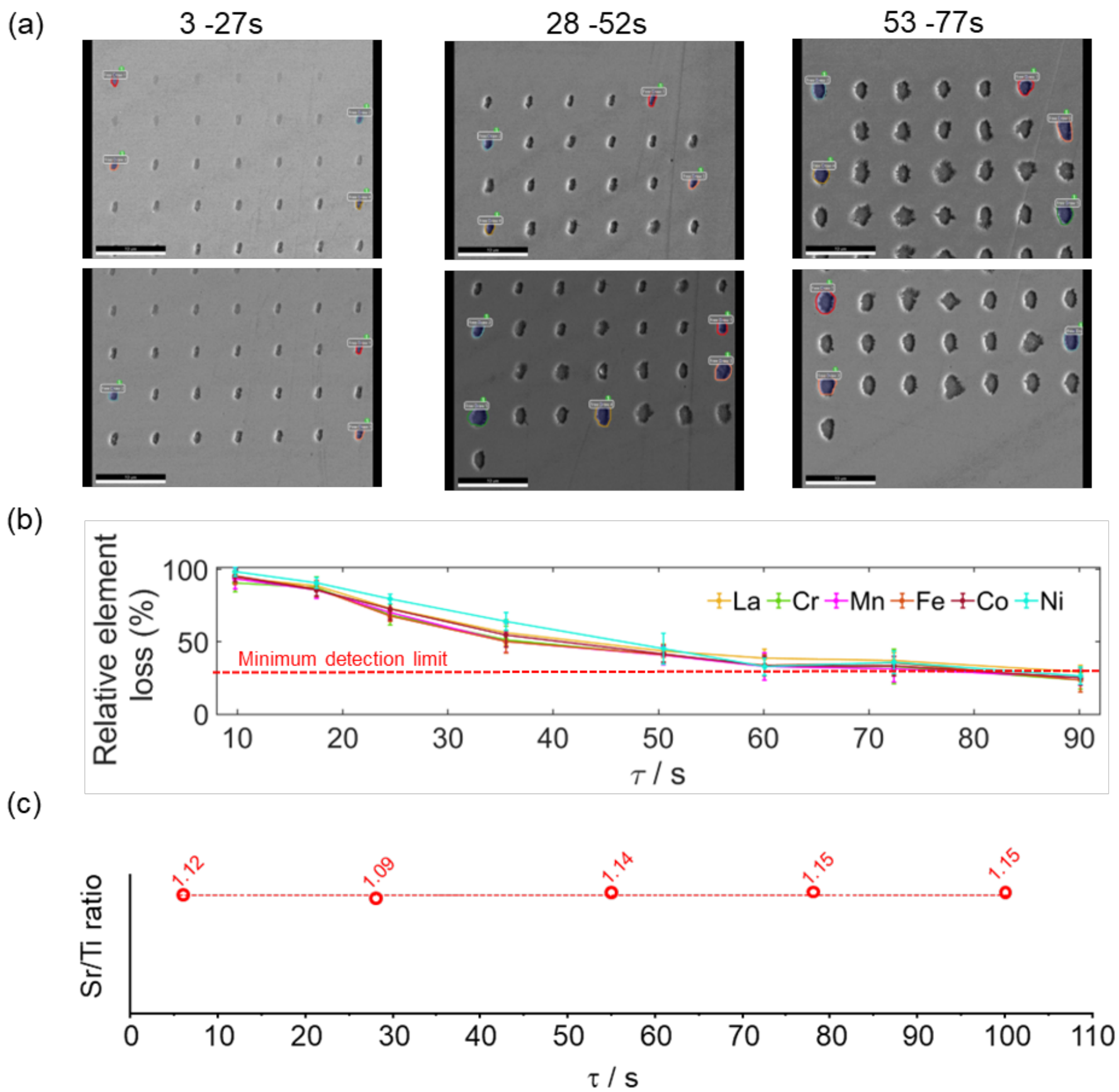

Figure S19. EDS spectra analysis for time-dependent *i–t* hopping experiments on La-HEO-P-89 nm. (a) Representative SEM image of EDS analysis; (b) Relative element loss for each element, present as a function of τ period; (c) substrate (Nb: STO) composition ratio as a

Section S3. AFM image analysis

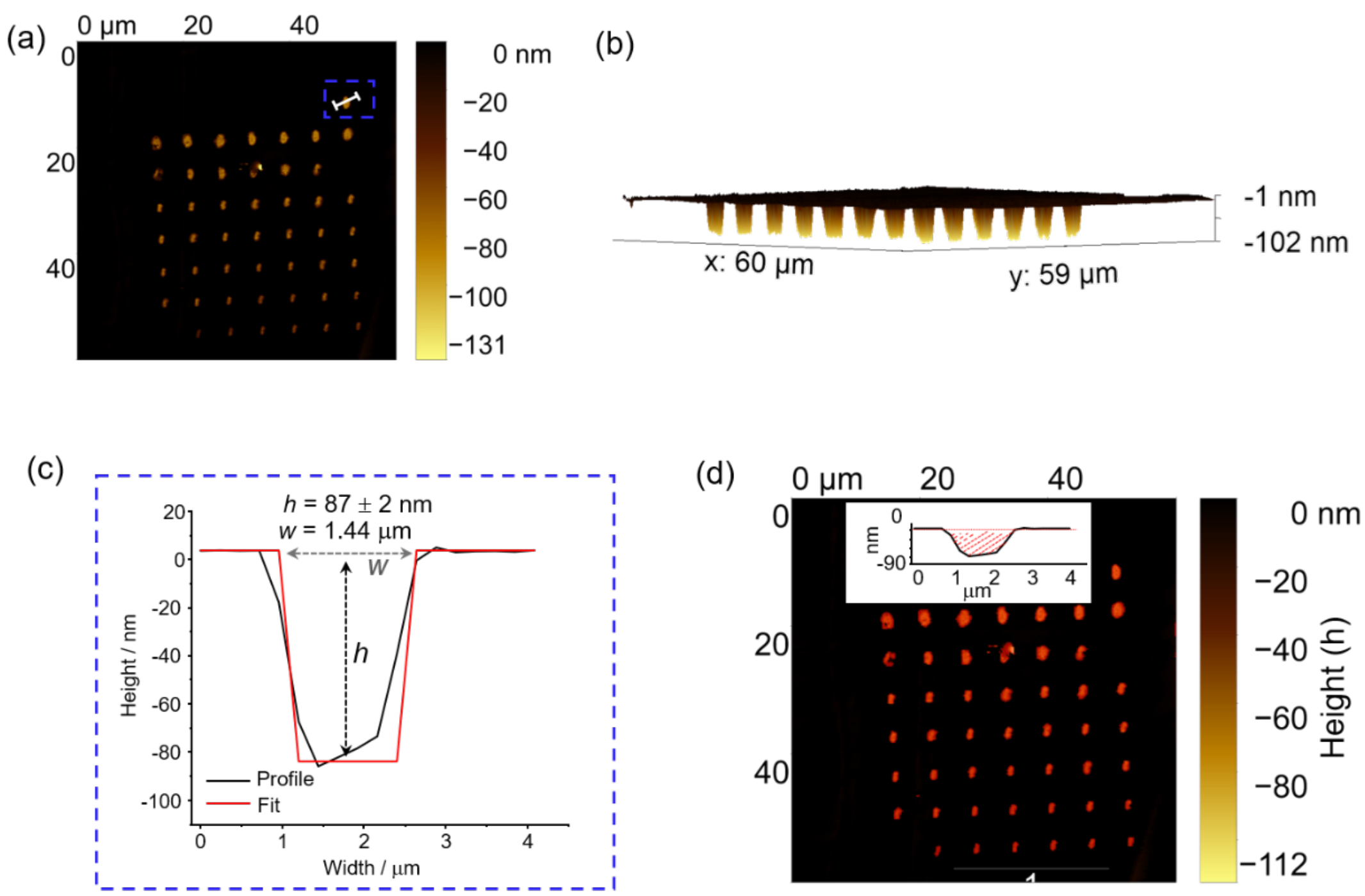

Figure S20. AFM image analysis for OER reaction area on La-HEO-P-89 nm.

(a,b) Top view and the side view of the AFM image of scan-hopping region electrode after background correction, where pristine surface height was referenced to zero, and blue squared area denotes the electrochemically probed region; (c) representative line-profile fit used to determine dissolution pit depth; and (d) Estimation of pit volume and surface area by marking (red) the pit by height thresholding (inset) where pits were identified as inverted grain.

Dissolution pit area and volume were extracted from AFM images using height-threshold segmentation following background leveling. Notably, the height threshold defines the reference level used to identify the pit opening. Thresholds set closer to the mean surface height approach the intrinsic surface roughness of the pristine film and can result in spurious inclusion of few surface regions. Conversely, more negative threshold values define the pit boundary deeper within

the pit, thereby excluding shallow regions near the pit opening and leading to an underestimation of pit area and volume. An intermediate threshold was therefore selected to balance these effects and provide a reliable representation of pit geometry. The same thresholding criterion was applied to all datasets to ensure consistency across measurements. All pit volumes and areas corresponding to the reaction times (τ) are provided in Table S2 and S3

Table S2. AFM analysis results as a function of pulse time for La-HEO-P-89 nm.

Experiment	Pulse time / s	Pit volume / um^3	Area / um^2
Scan hopping–I, Figure 5a	3		
	3.5		
	4		
	4.5		
	5		
	5.5		
	6		
	6.5		0.220
	7		0.385
	7.5	0.002	0.824
	8	0.004	0.714
	8.5		
	9	0.003	0.549
	9.5	0.007	0.824
	10	0.008	1.044
	10.5	0.008	0.934
	11	0.007	0.934
	11.5	0.011	1.044
	12	0.012	1.154
	12.5		
	13	0.013	1.045
	13.5	0.01	0.989
	14	0.017	1.265
	14.5		
	15	0.017	1.265
	15.5	0.018	1.155
	16	0.01	0.935
	16.5	0.018	1.210
	17	0.02	1.375
	17.5	0.019	1.101
	18	0.021	1.321
	18.5	0.022	1.375
	19	0.025	1.486
	19.5	0.02	1.047

	20	0.027	1.431
	20.5	0.025	1.322
	21	0.032	1.597
	21.5	0.03	1.377
	22	0.028	1.212
	22.5	0.036	1.653
	23	0.028	1.487
	23.5	0.034	1.433
	24	0.039	1.654
	24.5	0.036	1.488
	25	0.038	1.763
	25.5	0.04	1.653
	26	0.038	1.763
	26.5	0.04	1.873
	27		
Scan hopping–II, Figure 5b	28	0.041	1.434
	28.5	0.045	1.436
	29	0.05	1.438
	29.5	0.051	1.658
	30		1.549
	30.5	0.052	1.493
	31	0.068	1.551
	31.5	0.059	1.828
	32	0.064	1.715
	32.5	0.058	2.154
	33	0.066	1.716
	33.5	0.075	1.829
	34	0.069	
	34.5	0.075	1.886
	35	0.08	
	35.5		
	36	0.065	2.213
	36.5	0.077	1.773
	37	0.096	1.940
	37.5	0.079	2.652
	38	0.088	1.886
	38.5	0.085	2.166
	39	0.105	2.164
	39.5	0.101	2.767
	40	0.087	2.605
	40.5	0.113	2.111
	41	0.093	2.983
	41.5	0.088	2.219
	42	0.104	2.112
	42.5	0.099	2.551

	43	0.142	2.219
	43.5	0.117	3.702
	44	0.139	2.770
	44.5	0.108	2.385
	45		
	45.5	0.132	3.594
	46	0.205	4.863
	46.5	0.168	5.629
	47	0.174	3.873
	47.5	0.14	2.882
	48	0.106	3.919
	48.5	0.205	5.251
	49	0.244	5.142
	49.5	0.258	5.578
	50	0.231	4.541
	50.5	0.212	4.214
	51	0.2	3.769
	51.5	0.246	4.866
	52	0.193	3.557
Scan hopping–III, Figure 5c	53	0.144	3.098
	53.5	0.186	3.542
	54	0.189	3.929
	54.5	0.229	4.860
	55	0.185	3.655
	55.5	0.211	3.988
	56		
	56.5	0.219	4.911
	57	0.235	4.972
	57.5	0.211	4.204
	58	0.227	4.751
	58.5	0.229	4.697
	59	0.278	5.852
	59.5	0.279	5.474
	60	0.259	5.849
	60.5	0.246	5.080
	61	0.346	7.329
	61.5	0.221	4.367
	62	0.303	6.400
	62.5	0.231	4.423
	63	0.217	4.203
	63.5	0.309	6.179
	64	0.312	6.346
	64.5	0.238	4.537
	65	0.228	4.970
	65.5	0.254	4.814

	66	0.291	5.692
	66.5	0.216	4.262
	67	0.215	4.150
	67.5	0.206	4.205
	68	0.266	5.088
	68.5	0.325	6.840
	69	0.238	4.702
	69.5	0.261	5.140
	70	0.211	4.097
	70.5	0.223	4.700
	71	0.21	4.213
	71.5	0.23	4.372
	72	0.208	4.316
	72.5	0.336	6.950
	73	0.248	4.538
	73.5	0.289	5.473
	74	0.279	5.579
	74.5	0.263	5.085
	75	0.395	7.556
	75.5	0.257	4.868
	76	0.249	4.810
	76.5	0.268	5.143
	77	0.243	5.027
Scan hopping–IV, Figure 5d	78	0.123	3.728
	79	0.119	3.283
	80	0.22	4.579
	81	0.223	4.677
	82	0.289	5.816
	83	0.45	10.705
	84	0.281	6.360
	85	0.284	5.955
	86	0.353	6.601
	87	0.458	8.871
	88	0.42	8.596
	89	0.361	7.337
	90	0.343	6.451
	91	0.355	7.339
	92	0.352	7.142
	93	0.416	8.290
	94	0.387	7.931
	95	0.517	10.348
	96	0.432	8.772
	97	0.387	8.176
	98	0.488	9.018
	99	0.445	8.530

100	0.354	7.144
101	0.399	8.181
102	0.462	8.727
103	0.405	7.887
104	0.403	8.725
105	0.571	12.421
106	0.561	12.680
107	0.529	11.788
108	0.523	11.493
109	0.498	10.946
110	0.443	9.946
111	0.673	12.818
112	0.702	14.002
113	0.67	15.918
114	0.715	17.015
115	0.53	12.962
116		
117	0.599	14.798
118	0.452	9.466
119	0.952	19.785
120	0.749	16.865
121		
122	0.55	11.733
123	0.482	10.294
124	0.488	10.986
125	0.416	9.169
126	0.785	16.889

Table S3. AFM analysis results as a function of pulse time for LaSr-HEO-P-29 nm.

Experiment	Pulse / s	Volume / um^3	Area / um^2
Scan hopping–I, Figure 6a	6		
	6.5		0.11
	7		
	7.5		
	8		
	8.5		
	9		
	9.5		
	10		0.165
	10.5		0.111
	11		0.111

	11.5		0.111
	12		0.112
	12.5	0.001	0.11
	13		0.110
	13.5		0.111
	14		
	14.5	0.002	0.165
	15		
	15.5		0.659
	16		
	16.5		
	17	0.004	0.607
	17.5		1.322
	18	0.003	0.824
	18.5	0.008	
	19	0.007	1.103
	19.5	0.004	1.541
	20	0.009	1.374
	20.5	0.005	1.158
	21	0.007	1.377
	21.5	0.017	0.938
	22	0.021	1.367
	22.5		
	23		
	23.5		
	24	0.017	1.762
	24.5	0.019	1.928
	25	0.025	1.87
	25.5	0.015	1.542
	26	0.02	1.708
	26.5	0.024	1.926
	27	0.022	1.984
Scan hopping–II, Figure 6b	27.5	0.028	1.982
	28	0.022	1.762
	28.5	0.026	2.038
	29		
	29.5		
	30	0.027	1.872
	30.5	0.03	1.762
	31	0.028	1.763
	31.5	0.025	1.873
	32	0.019	1.762
	32.5	0.025	1.489
	33	0.024	1.763
	33.5	0.025	1.598

	34	0.026	1.654
	34.5	0.03	1.764
	35	0.026	1.763
	35.5	0.026	1.597
	36	0.029	1.654
	36.5	0.026	1.875
	37	0.026	1.817
	37.5	0.026	1.818
	38	0.03	1.816
	38.5	0.032	1.927
	39	0.036	1.872
	39.5	0.031	1.873
	40	0.027	1.598
	40.5	0.033	1.983
	41	0.027	1.651
	41.5		
	42	0.031	1.928
	42.5		
	43	0.03	1.817
	43.5	0.03	1.762
	44		
	44.5	0.034	1.709
	45	0.036	1.983
	45.5		
	46	0.03	1.817
	46.5		
	47		
	47.5		
	48		
Scan hopping–III, Figure 6c	48.5	0.039	2.647
	49	0.026	2.513
	49.5	0.045	3.686
	50	0.047	3.3
	50.5	0.049	3.519
	51	0.035	2.697
	51.5	0.039	2.805
	52	0.034	2.585
	52.5	0.042	3.192
	53	0.042	3.025
	53.5	0.047	3.189
	54	0.045	2.915
	54.5	0.04	2.915
	55	0.035	2.917
	55.5	0.039	2.804
	56	0.043	2.861

56.5	0.042	3.027
57	0.036	2.695
57.5	0.044	3.134
58	0.038	2.642
58.5	0.036	2.584
59	0.034	2.696
59.5	0.041	2.971
60	0.045	3.245
60.5	0.04	2.751
61	0.04	2.861
61.5	0.039	2.696
62	0.047	3.19
62.5		
63	0.038	2.75
63.5		
64	0.036	2.639
64.5	0.043	3.138
65	0.04	3.026
65.5		
66	0.035	3.3
66.5	0.036	3.521
67	0.04	3.52
67.5	0.027	2.916
68	0.04	3.355
68.5	0.043	3.19
69	0.044	3.465
69.5	0.043	3.578
70	0.041	3.244
70.5	0.035	3.024
71	0.044	3.301
71.5	0.041	3.41
72	0.035	2.916

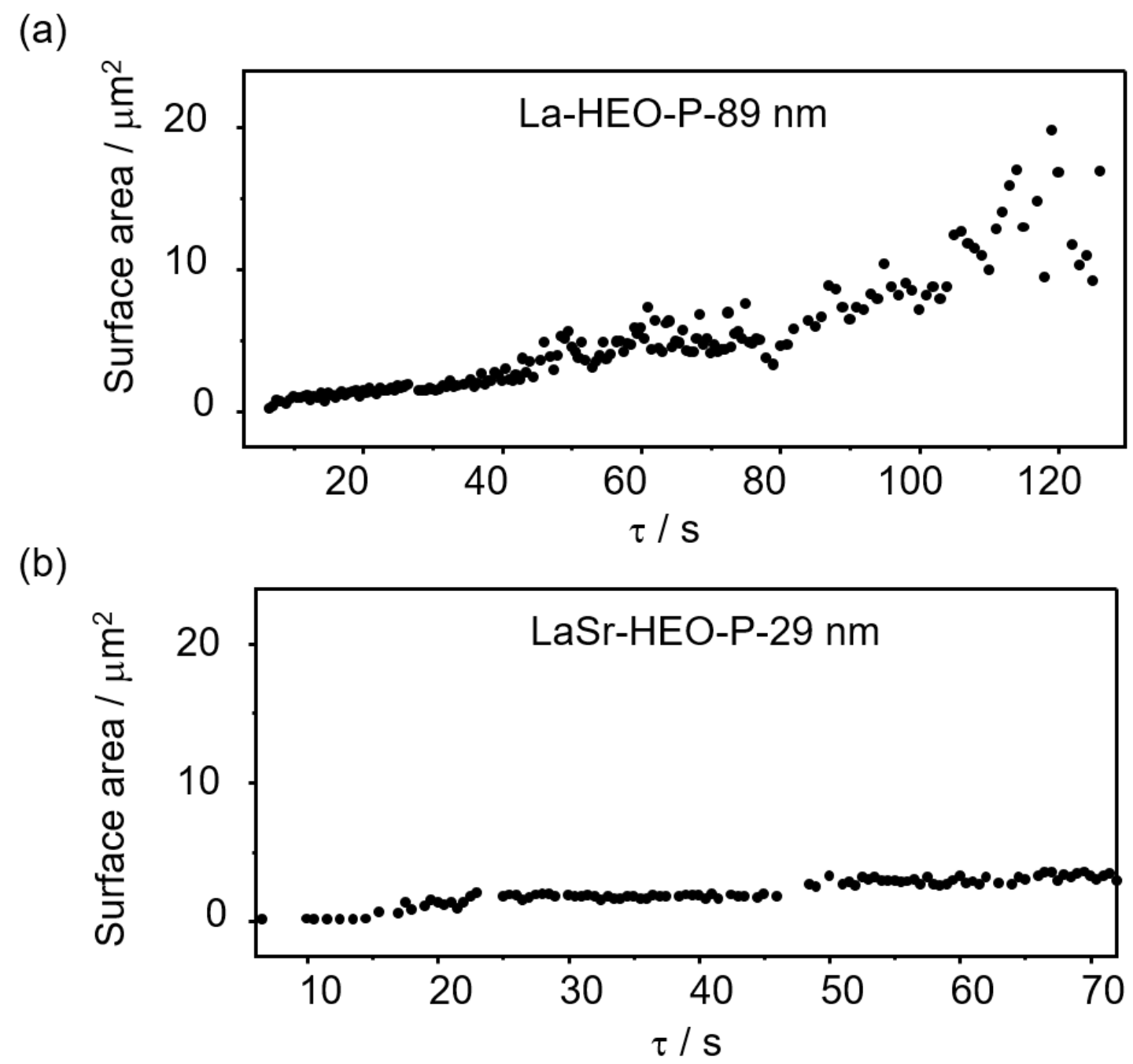

Figure S21. (a) Surface area of the dissolution pits as a function of τ for La-HEO-P-89 nm; (b) for LaSr-HEO-P-29 nm.

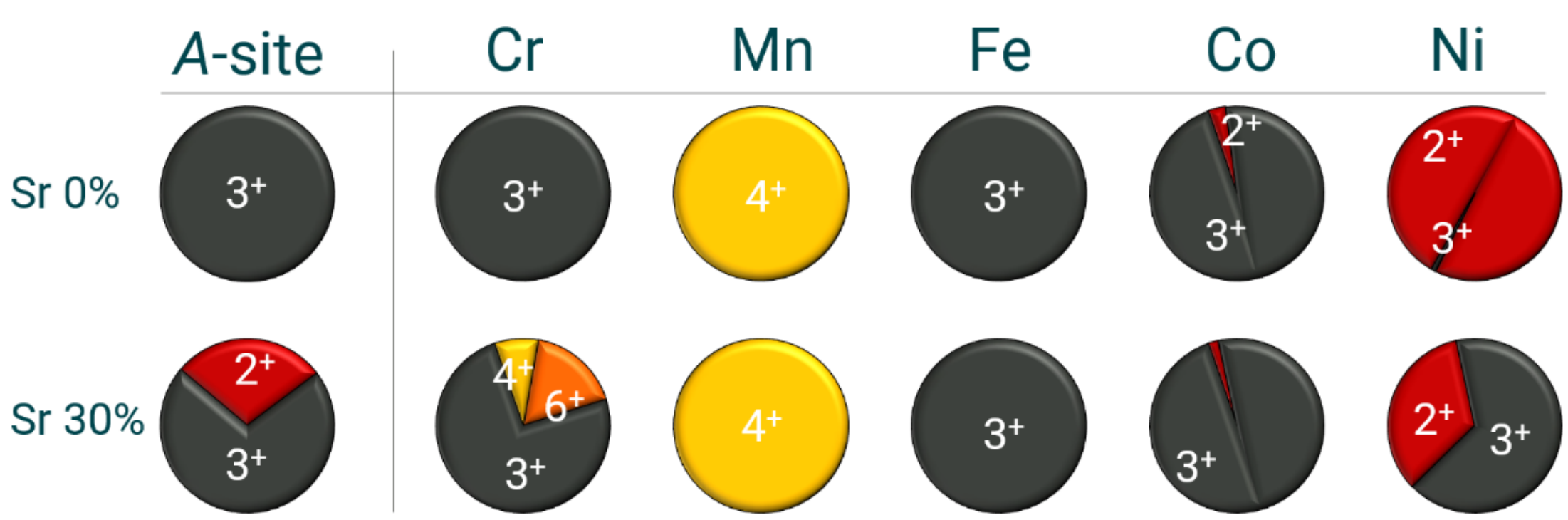

Figure S22. Observed changes to cation charge state population relative to *A*-site doping.

Section S4. Conversion of pit volume to mass and charge

The density (ρ) and unit cell parameters of HEO-P materials were obtained from previous literature. For La-HEO-P, the refined orthorhombic *Pbnm* structure gives a theoretical density of 6.92 g cm^{-3}.[1]

For LaSr-HEO-P, the cubic lattice parameter (a_0 = 3.890 Å) was used from the previous literature to calculate the ρ -[2]

$$\rho = \frac{M}{N_A a_0^3} \tag{S1}$$

where M (≈ 227.60 g mol^{-1}) is the molar mass of LaSr-HEO-P, a_0^3 is the unit cell volume from pseudo-cubic lattice parameter (a_0), and N_A is Avogadro's number. The ρ for the LaSr-HEO-P was calculated to be ~ 6.42 g cm^{-3}.

(2) Conversion of pit volume to theoretical upper-limit charge

The faradaic charge equivalent of the HEO-P mass (from AFM pit volume) was calculated from the following formula

$$Q_{pit,HEO-P} = \rho_{HEO-p} \times V_{pit,HEO-P} \times \frac{Q}{m} \tag{S2}$$

where ρ is the density of HEO-P, V is the pit volume, and Q/m is the conversion factor which defines how much charge is needed for 1 picogram (pg) HEO-P dissolution.

Q/m was derived using the following equation

$$Q/_{m} = {(n_e \times F)}/_{M_{\mathrm{HEO-P}}} \tag{S3}$$

where n_e is the total number of electrons theoretically involved during oxidation of the one formula unit lattice.

Upper limit of n_e can be calculated from the contribution from oxidation of metals to the highest thermodynamically stable oxidation state (n_metal), and the other contributions from oxide oxidation from the budget of total lattice oxygen (n_oxide) to oxygen.

$$n_\mathrm{e} = n_\mathrm{metal} + n_\mathrm{oxide} \tag{S4}$$

where,

$$n_{metal} = \sum_i f_i \Delta n_i \tag{S5}$$

n_{metal} is the total effective metal contribution per formula unit, f_i is the molar fraction of metal i (for equimolar B-site metals, f_i = 0.2), Δn_i is the oxidation-state change (number of electrons transferred) from the initial oxidation state associated with metal i in the lattice, and n_{oxide} is the oxidation charge from the total lattice oxygen budget involves 6 electrons (1.5 mol oxygen) oxidation per formula unit (ABO_3).

$$3O^{2-} = \frac{3}{2}O_2 + 6e^-$$

F = 96485 C/mol and $M_{\mathrm{HEO-P}}$ is the molar mass (242.98 g/mol and 227.60 g/mol for La-HEO-P and LaSr-HEO-P, respectively).

The effective Δn_i was calculated as the difference between the highest thermodynamically stable and soluble oxidation state and the initial oxidation state of ith metal, weighted by the relative population of each oxidation state present in the pristine lattice (Figure S22).[3]

Table S4. Change of Oxidation states for HEO-P compositions

Catalyst	Composition	Δn_{Cr}*	Δn_{Mn}*	Δn_{Fe}*	Δn_{Co}*	Δn_{Ni}*	$\Delta n_{\mathrm{La,Sr}}$*
La-HEO-P	$La(Cr_{0.2}Mn_{0.2}Fe_{0.2}Co_{0.2}Ni_{0.2})O_3$	+3	+3	0	+0	+2	0
LaSr-HEO-P	$La_{0.7}Sr_{0.3}(Cr_{0.2}Mn_{0.2}Fe_{0.2}Co_{0.2}Ni_{0.2})O_3$	+1.5	+3	0	0	+1	0

*The highest thermodynamically stable and soluble oxidation state was assigned from Pourbaix diagrams and previously reported literature data.[4-6]

For La-HEO-P,

B-site ($Cr_{0.2}Mn_{0.2}Fe_{0.2}Co_{0.2}Ni_{0.2}$) contribution = n_{metal} = (3 + 3 + 0 + 0 + 2) × 0.2 = 1.6e⁻/formula unit. Lattice-oxygen contribution (n_{oxide}) ~ 6e⁻/formula unit.

n_{e} = 7.6e⁻/formula unit.

$$Q/m = \frac{n_e \times F}{M_{\mathrm{La-HEO-P}}} \times 10^{-3} = 3.02 \text{ nC/picogram}$$

For LaSr-HEO-P,

B-site ($Cr_{0.2}Mn_{0.2}Fe_{0.2}Co_{0.2}Ni_{0.2}$) contribution = n_{metal} = (1.5 + 3 + 0 + 0 + 1) × 0.2 = 1.1e⁻/formula unit. Lattice-oxygen contribution (n_{oxide}) ~ 6e⁻/formula unit.

n_e = 7.1e$^-$/formula unit

$$Q/m = \frac{n_e \times F}{M_{\text{LaSr-HEO-P}}} \times 10^{-3} = 3.01 \text{ nC/picogram}$$

$$Q_{\text{pit,La-HEO-P}} = \rho_{\text{La-HEO-P}} \times V_{\text{pit,La-HEO-P}} \times 3.02$$

$$Q_{\text{pit,LaSr-HEO-P}} = \rho_{\text{LaSr-HEO-P}} \times V_{\text{pit,LaSr-HEO-P}} \times 3.01$$

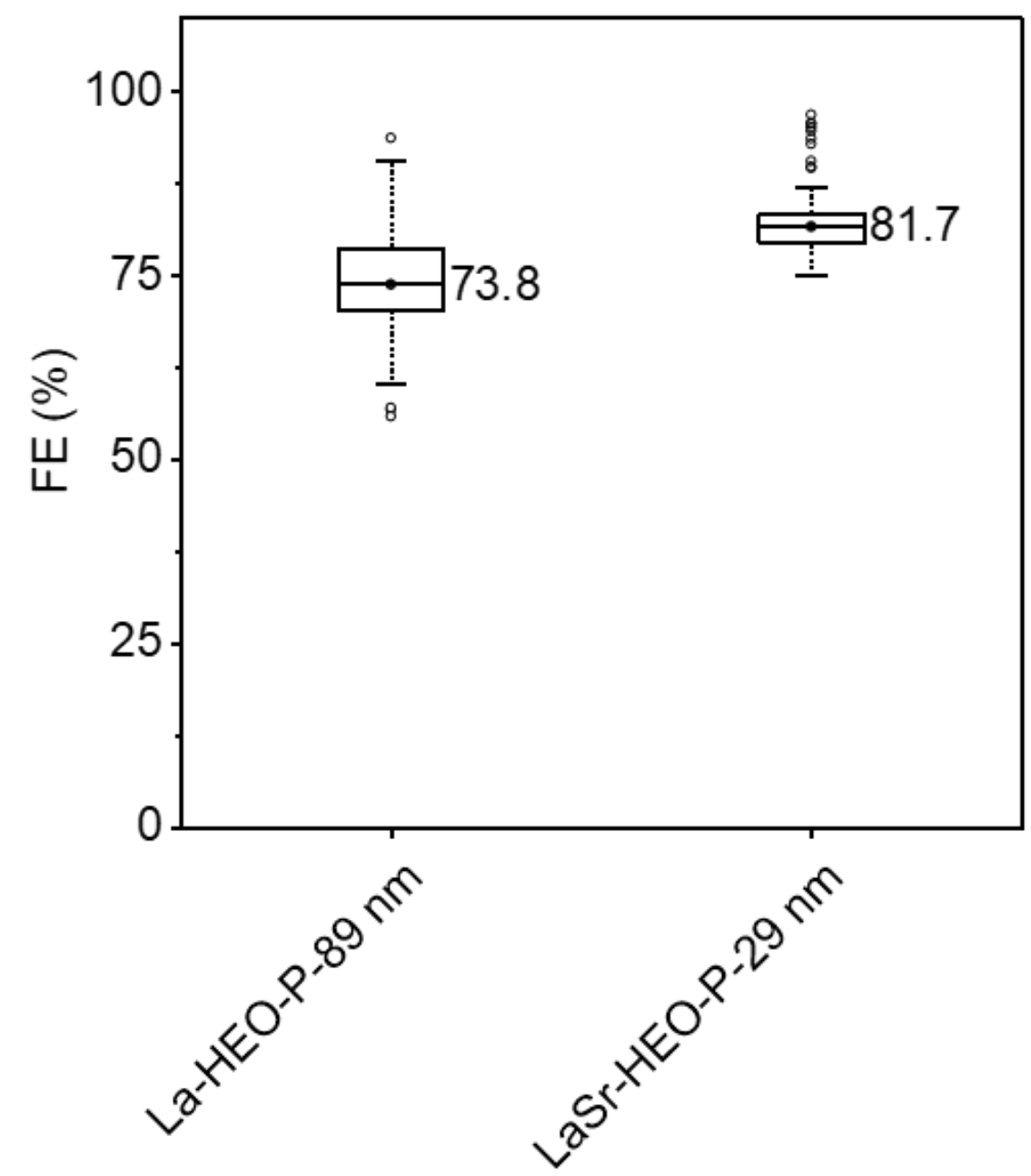

Figure S23. Box plot with median faradaic efficiency value, where box width represents the difference between the 1st quartile and the 3rd quartile of the dataset, and black circles are outlier points.

Faradaic efficiency (FE) of OER was calculated based on the charge expended for dissolution (Q_{pit}) and OER (Q_{total} - Q_{pit}) using the following equation-

$$FE\ (\%) = \frac{Q_{total} - Q_{pit}}{Q_{total}} \times 100$$

Section S5 Thickness dependence study for both HEO-P electrode

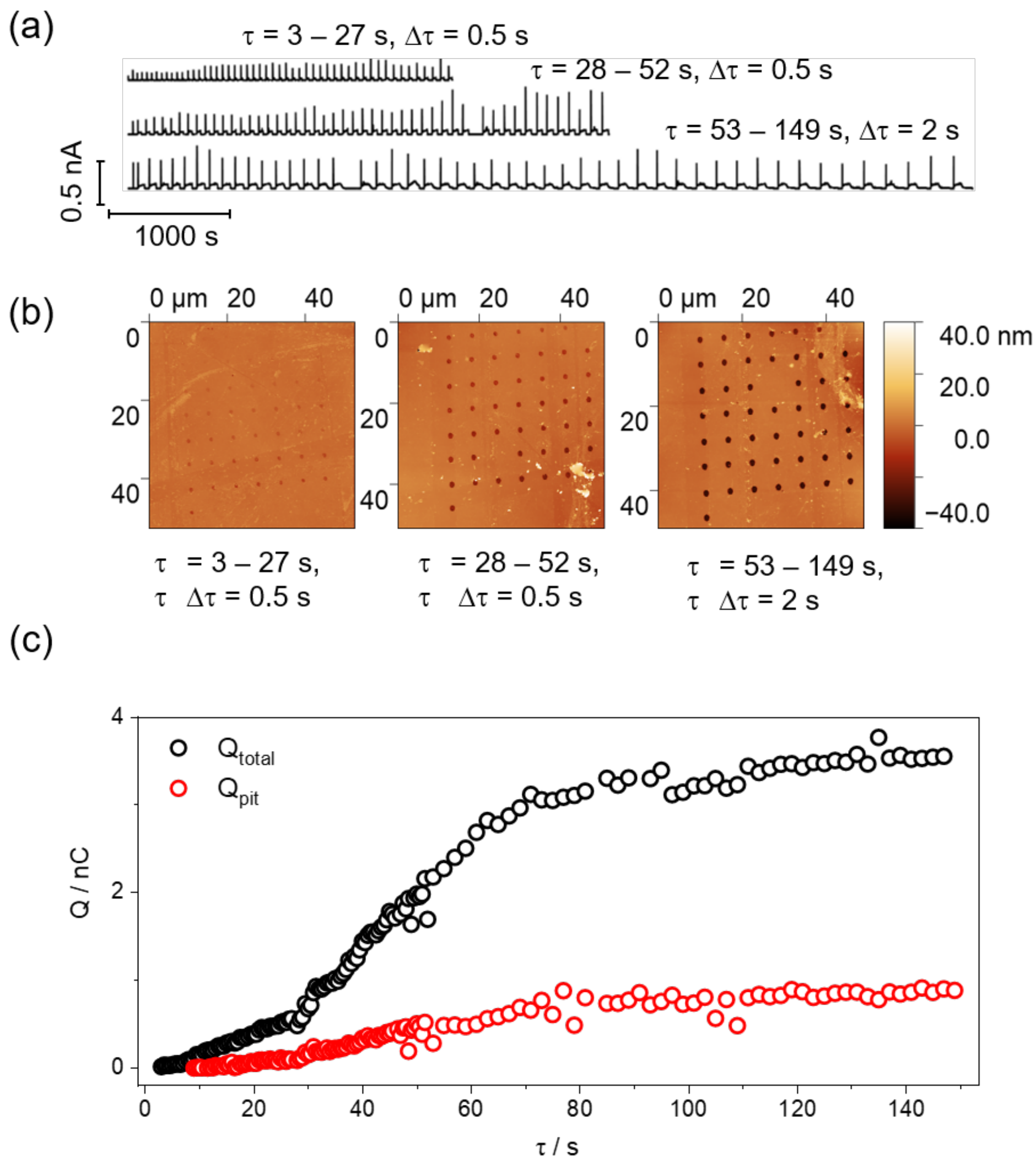

Figure S24. (a) *i–t* traces from each scan-hopping on La-HEO-P-29 nm. The traces are arranged from top to bottom, where each scan-hopping sequence consists of 49 *i–t* measurements recorded from the initial pulse time τ to $\tau + (48 \times \Delta\tau)$ at constant overpotential. Missing *i–t* traces correspond to false-approach events (no meniscus contact with surface); (b) *Ex-situ* AFM characterization of La-HEO-P-29 nm electrode after scan-hopping experiment; (c) Q_{total} and Q_{pit} as a function of τ for La-HEO-P-29 nm.

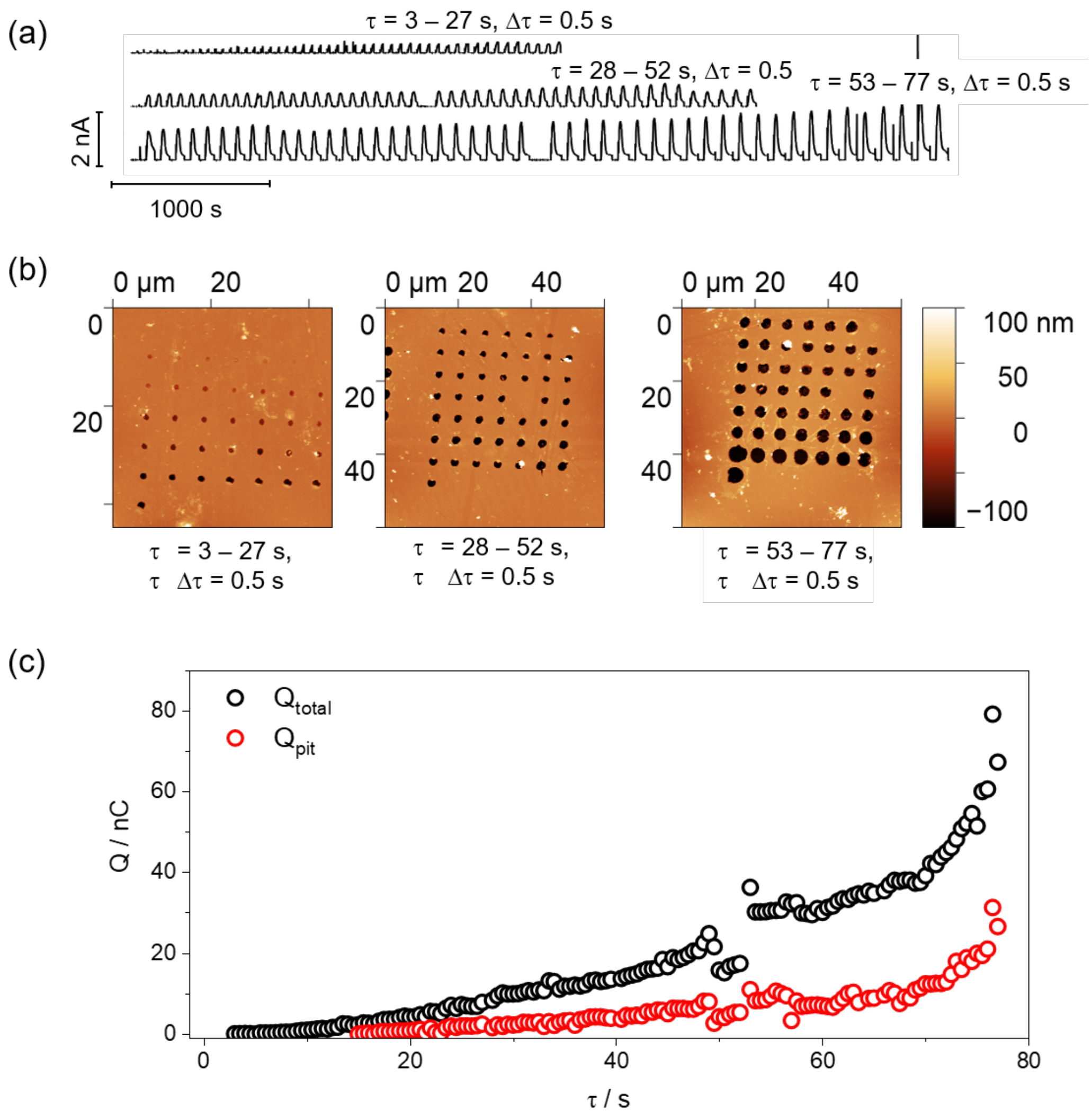

Figure S25. (a) *i*–*t* traces from each scan-hopping on LaSr-HEO-P-100 nm. The traces are arranged from top to bottom, where each scan-hopping sequence consists of 49 *i*–*t* measurements recorded from the initial pulse time τ to $\tau + (48 \times \Delta\tau)$ at constant overpotential. Missing *i*–*t* traces correspond to false-approach events (no meniscus contact with surface); (b) *Ex-situ* AFM characterization of LaSr-HEO-P-100 nm electrode after scan-hopping experiment; (c) Q_{total} and Q_{pit} as a function of τ for LaSr-HEO-P-100 nm.

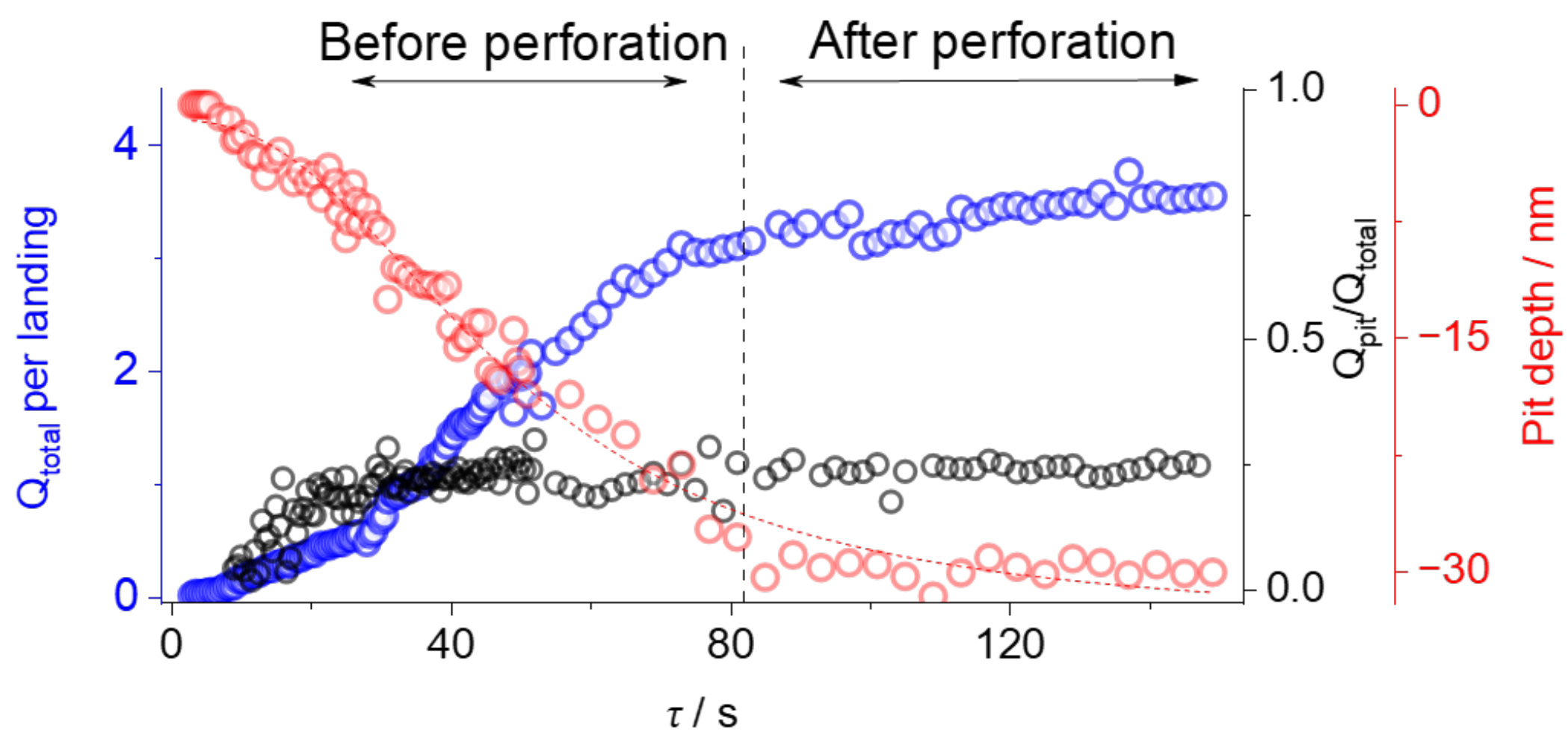

Figure S26. Correlation among Q_{total} (blue circle plot), attenuation of pit depth (red circle plot) and Q_{pit}/Q_{total} (black circle) as a function τ for La-HEO-P-29 nm. The vertical dashed line in the figure represents the τ when the pit depth reaches the substrate, as determined by AFM.

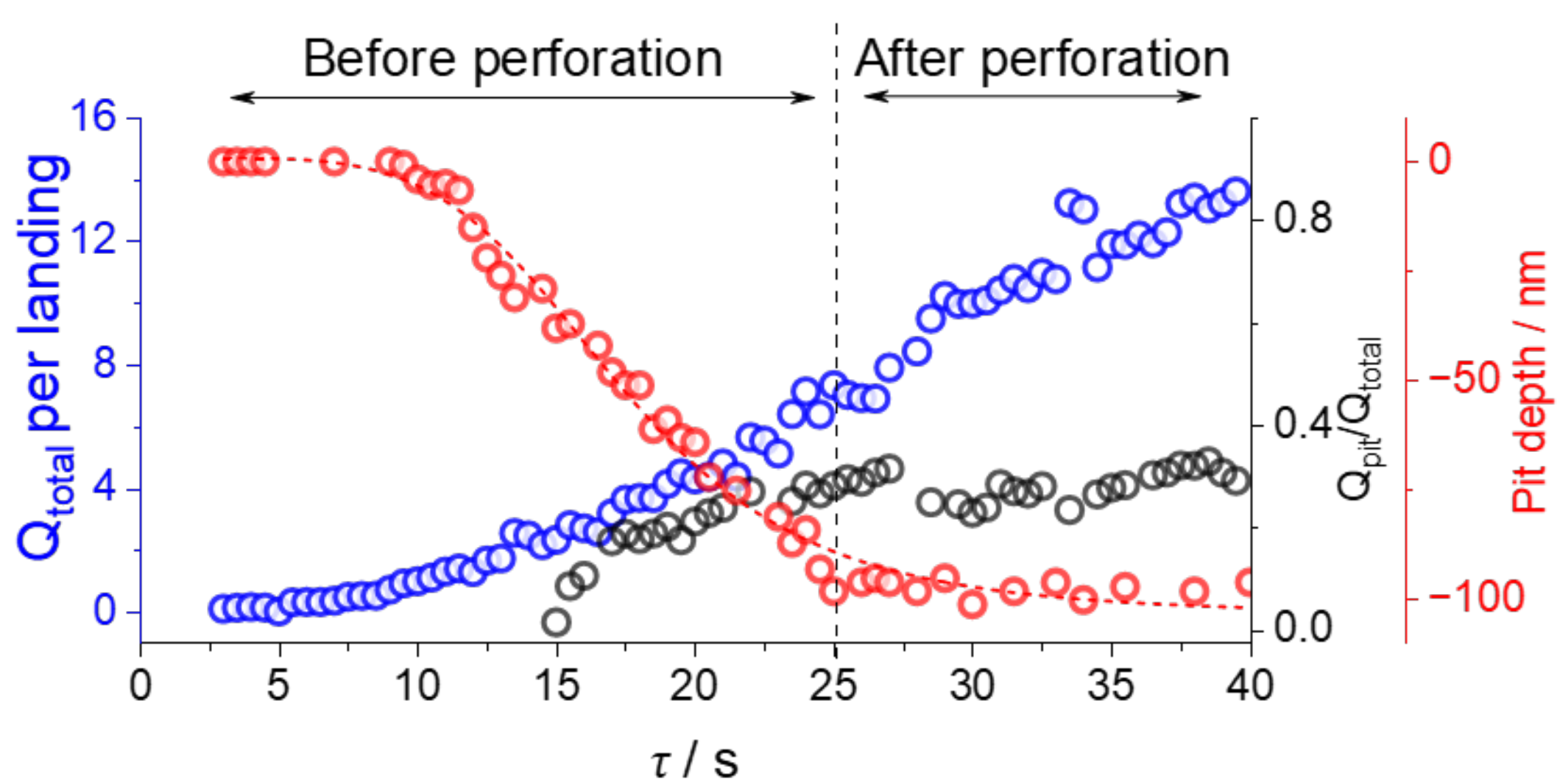

Figure S27. Correlation among the Q_{total} (blue circle plot), attenuation of pit depth (red circle plot) and Q_{pit}/Q_{total} (black circle) as a function τ for LaSr-HEO-P-100 nm. The vertical dashed line in the figure represents the τ, when the pit depth reaches the substrate, as determined by AFM.

Section S6. Experimental details of hop scanning with *i-t* on HEO-P electrode as a function of η

The *i–t* traces were recorded using a single nanopipette with a radius of approximately ~ 740 nm. Before the scan-hopping experiments, the QRCE was activated by anodizing in a saturated KCl solution. The approach voltage and feedback threshold current were optimized to minimize false landing events during measurements. Lower threshold values resulted in false landings, whereas higher values caused early perturbation of the pristine HEO-P surface, occurring on timescales shorter than those relevant to the experiment. The scan-hopping experiments were conducted using the parameters summarized in Table S5.

Table S5. Parameters for the scan-hopping experiments

Parameters	Values
Voltage to working electrode (V vs RHE)	414–514 mV vs E^0_{OER}
Feedback threshold (pA)	11 pA
Hopping distance (µm)	6
Retract rate (µm/s)	1
Approach rate (µm/s)	0.15
Scan area (µm^2)	36 × 36

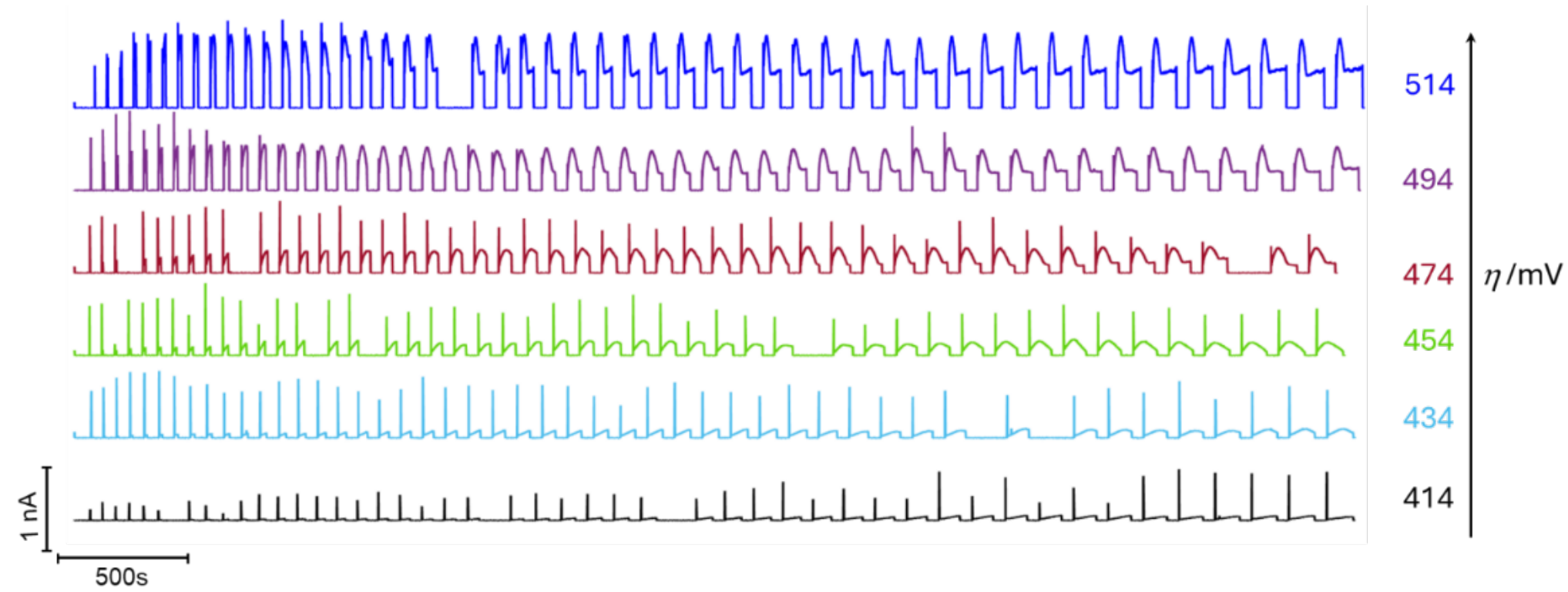

Figure S28. *i–t* traces collected from each scan-hopping experiment as a function of η for La-HEO-P-89 nm. Traces are arranged from top to bottom, where each scan-hopping sequence consists of 49 *i–t* measurements recorded within a fixed time window (4–100 s, τ = 4 s, $\Delta\tau$ = 2 s) at different overpotentials. Missing *i–t* traces correspond to false-approach events.

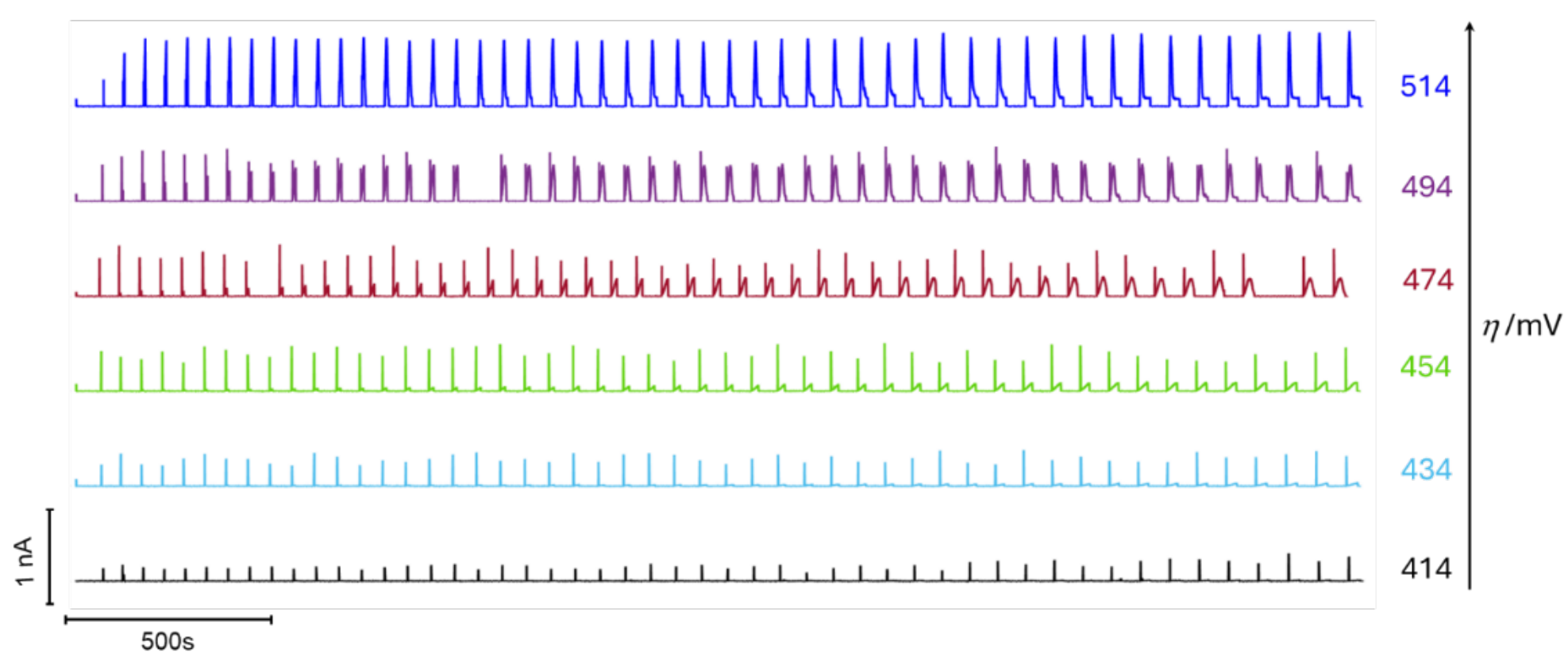

Figure S29. *i–t* traces collected from each scan-hopping experiment as a function of η for LaSr-HEO-P-29 nm. Traces are arranged from top to bottom, where each scan-hopping sequence consists of 49 *i–t* measurements recorded within a fixed time window (3–27 s, τ = 3 s, $\Delta\tau$ = 0.5 s) at different overpotentials. Missing *i–t* traces correspond to false-approach events.

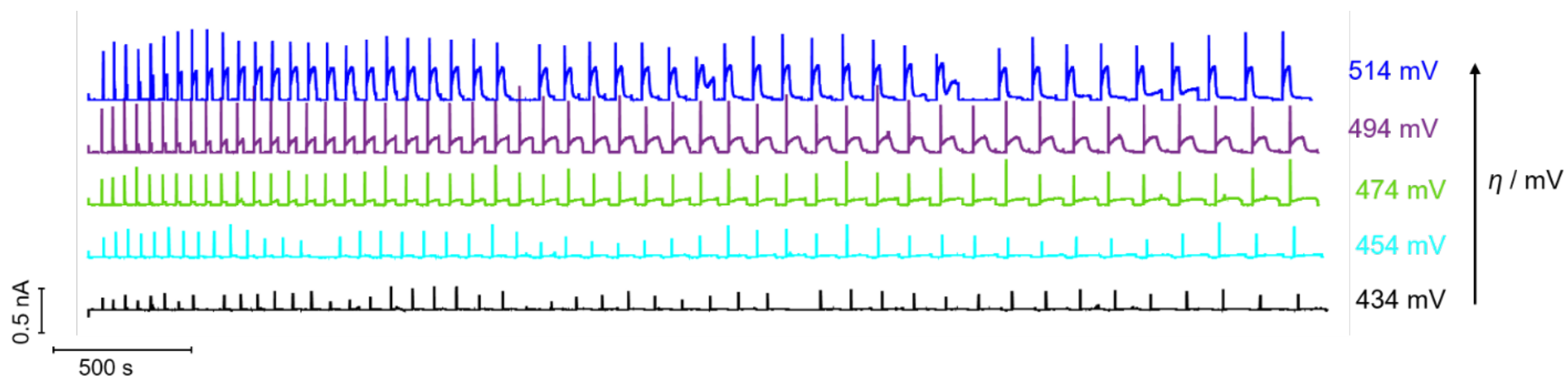

Figure S30. *i–t* traces collected from each scan-hopping experiment as a function of η for La-HEO-P-29 nm. Traces are arranged from top to bottom, where each scan-hopping sequence consists of 49 *i–t* measurements recorded within a fixed time window (4–100 s, τ = 4 s, $\Delta\tau$ = 2 s) at different overpotentials. Missing *i–t* traces correspond to false-approach events.

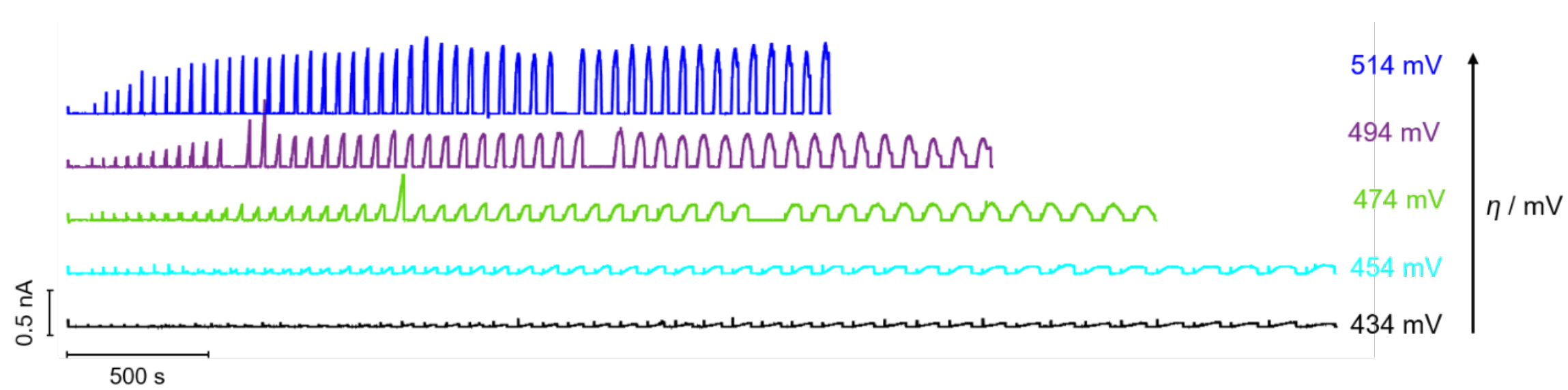

Figure S31. *i–t* traces collected from each scan-hopping experiment as a function of η for LaSr-HEO-P-100 nm. Traces are arranged from top to bottom, where each scan-hopping sequence consists of 49 *i–t* measurements recorded within a optimal time window (τ = 3 s, $\Delta\tau$ = 2 s for 434 mV, 454 mV; τ = 2 s, $\Delta\tau$ = 1.5 s for 474 mV; τ = 2 s, $\Delta\tau$ = 1 s for 494 mV ; and τ = 2 s, $\Delta\tau$ = 0.5 s for 514 mV) at different overpotentials. Missing *i–t* traces correspond to false-approach events.

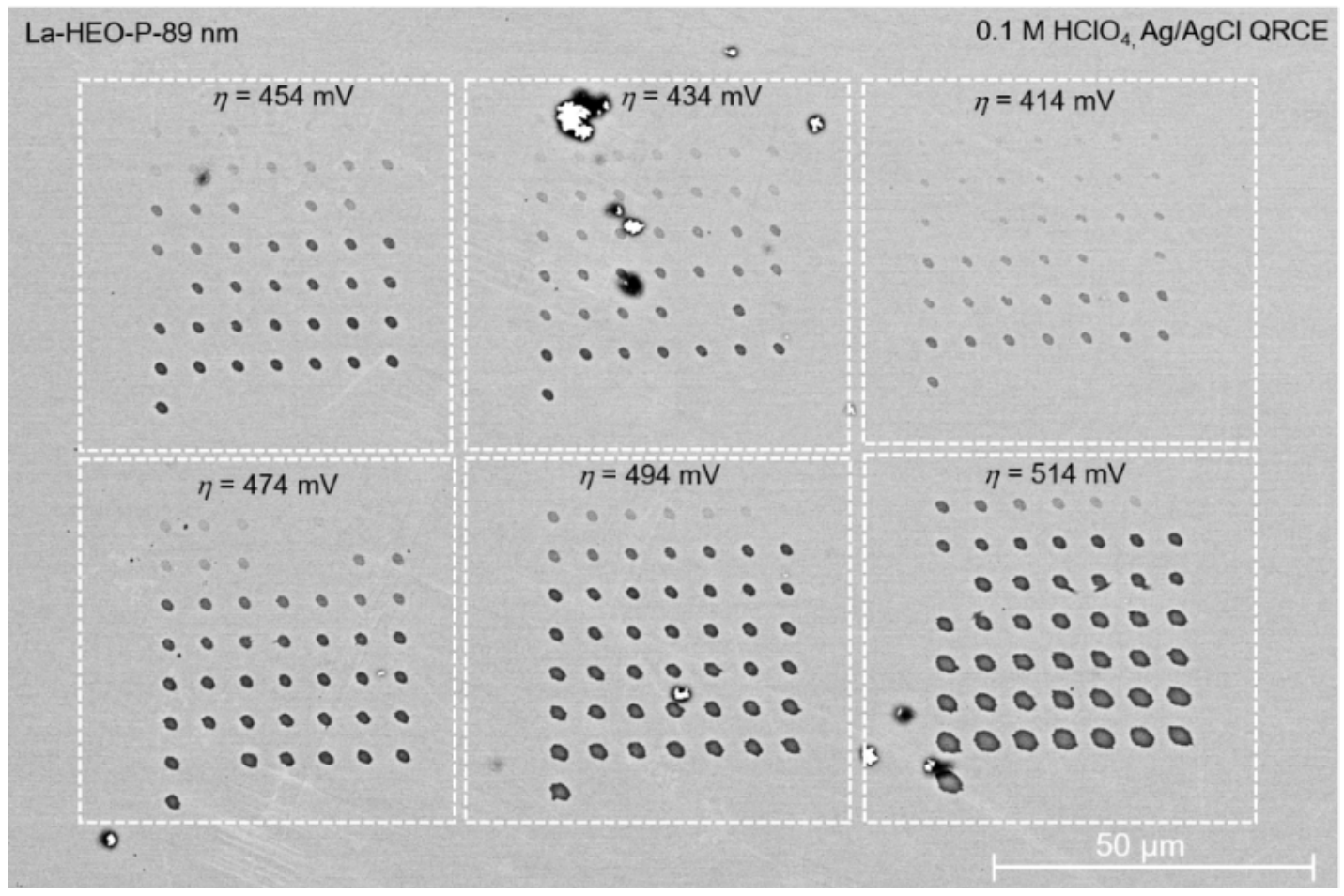

Figure S32. Representative SEM image of the *i–t* hopping experiments as a function of η on the La-HEO-P-89 nm electrode. The dashed boxes (white) indicate regions where measurements were performed at different η within a fixed time window (4–100 s, τ = 4 s, $\Delta\tau$ = 2 s).

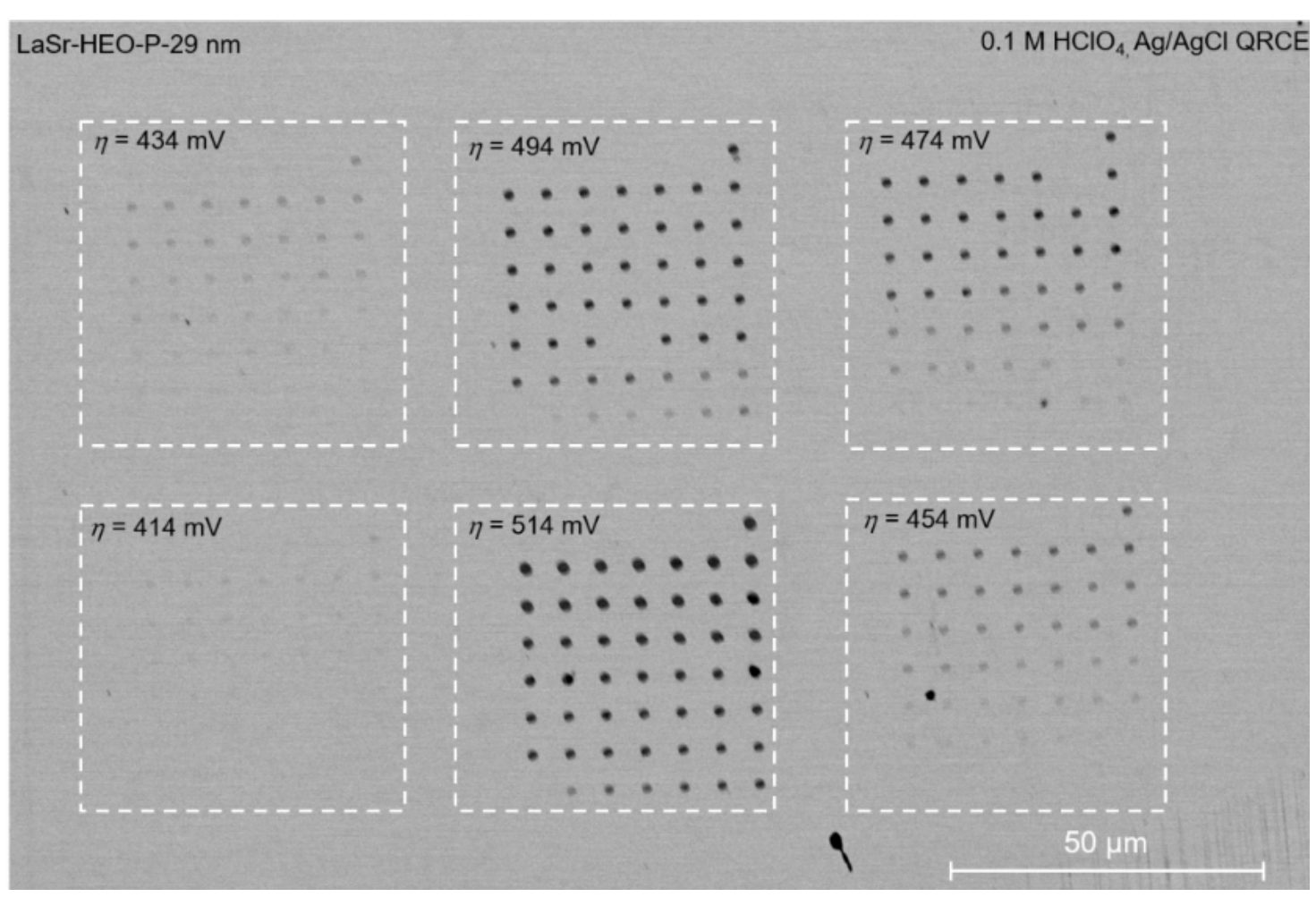

Figure S33. Representative SEM image of the *i–t* hopping experiments as a function of η at different overpotentials on LaSr-HEO-P-29 nm electrode. The dashed boxes (white) indicate regions where measurements were performed at different η within a fixed time window (3–27 s, τ = 3 s, $\Delta\tau$ = 0.5 s).

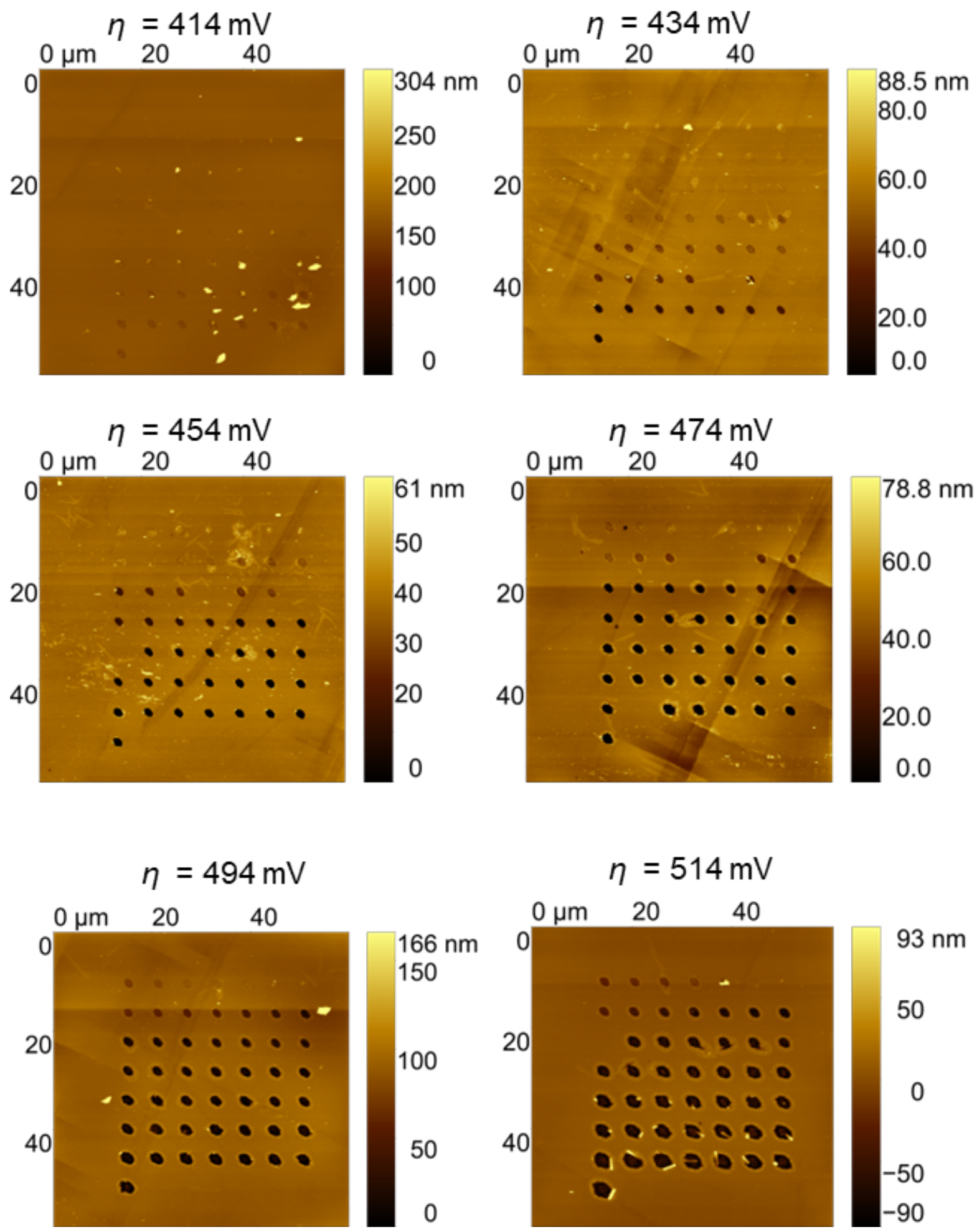

Figure S34. AFM image of the area where *i–t* hopping experiments as a function of η were performed on La-HEO-P-89 nm electrode.

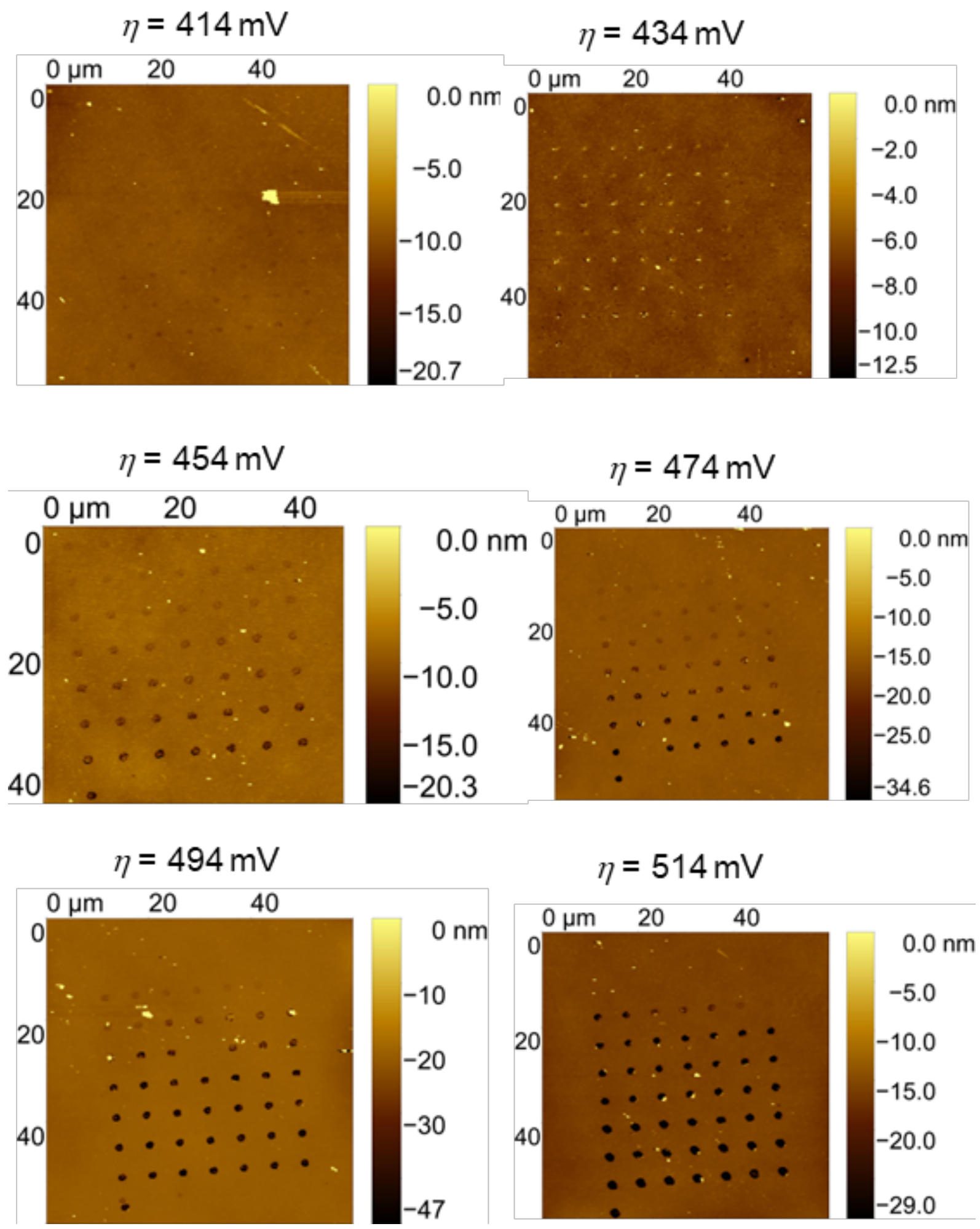

Figure S35. AFM image of the area where *i–t* hopping experiments as a function of η were performed on LaSr-HEO-P-29 nm electrode.

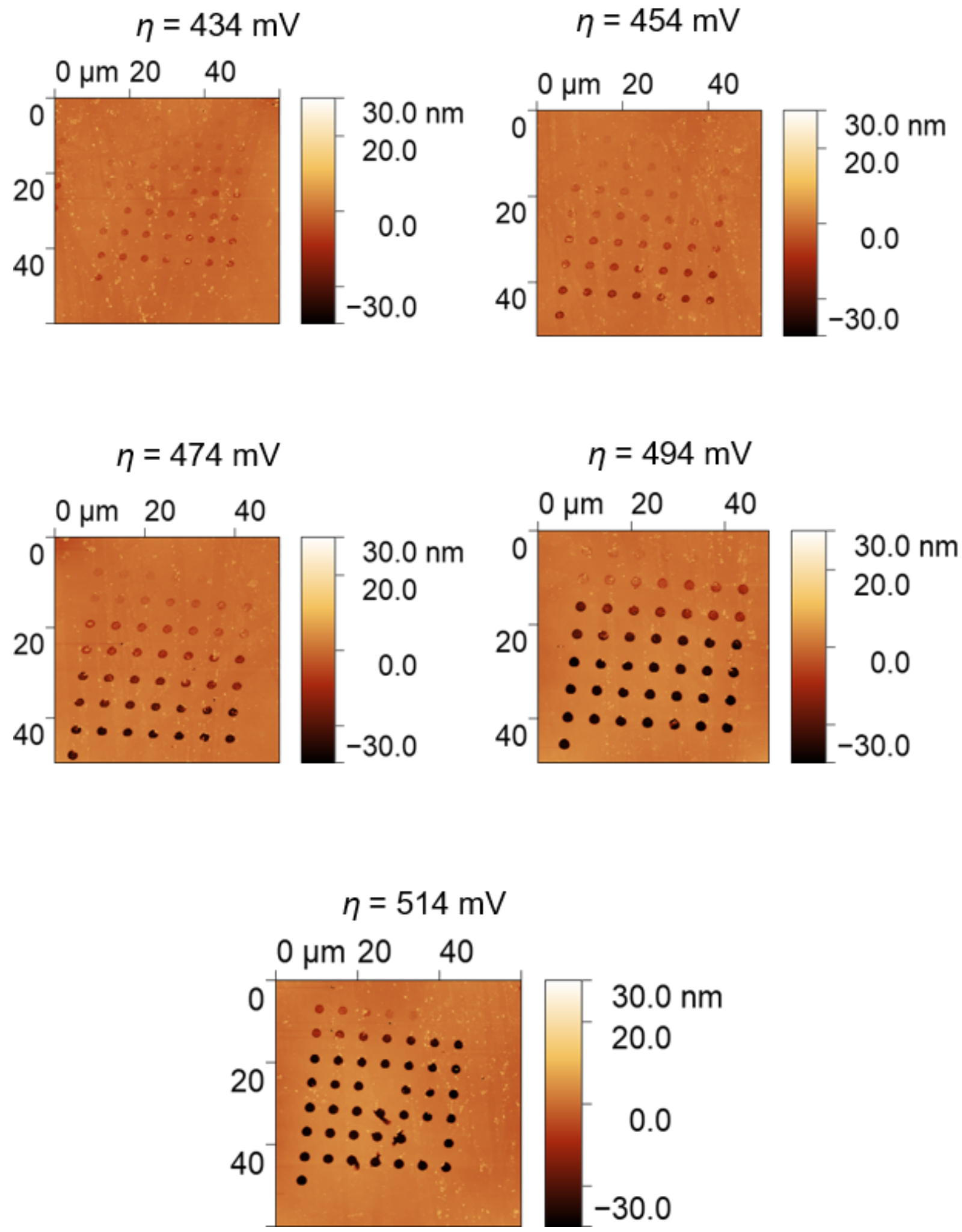

Figure S36. AFM image of the area where *i*–*t* hopping experiments as a function of η were performed on La-HEO-P-29 nm electrode.

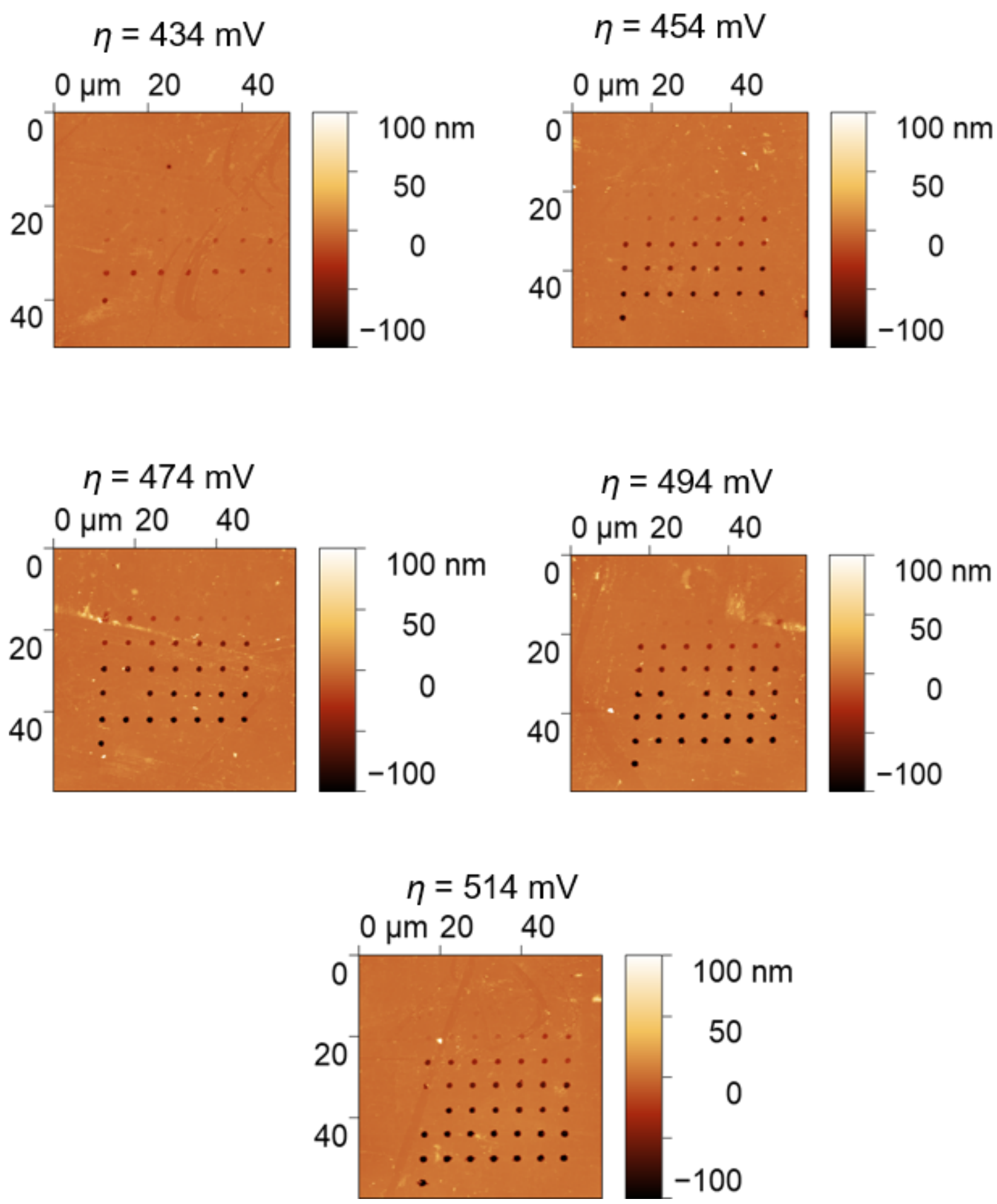

Figure S37. AFM image of the area where *i–t* hopping experiments as a function of η were performed on LaSr-HEO-P-100 nm electrode.

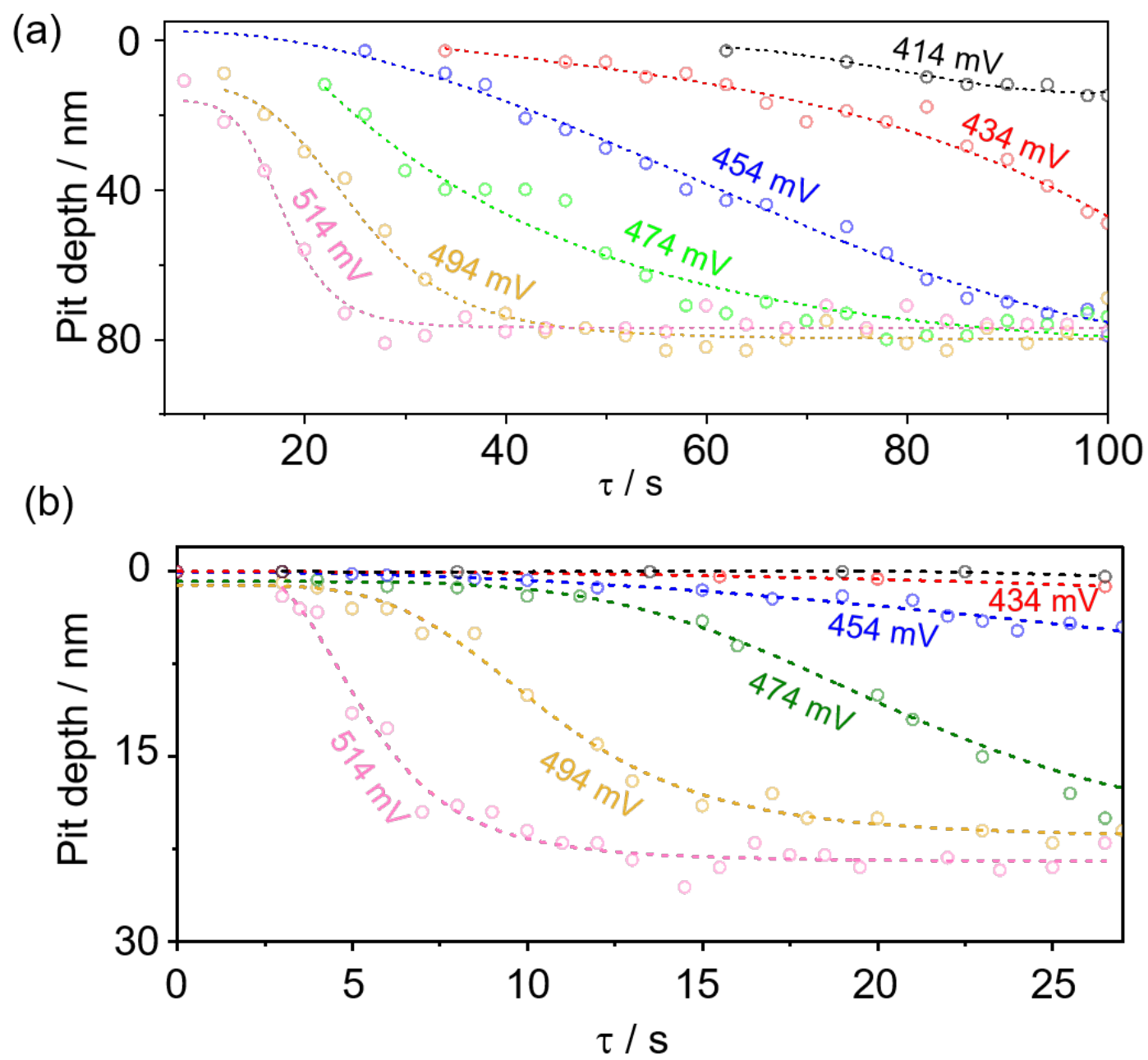

Figure S38. (a) Representative plot for pit depth attenuation as a function of η for La-HEO-P-89 nm and for (b) LaSr-HEO-P-29 nm.

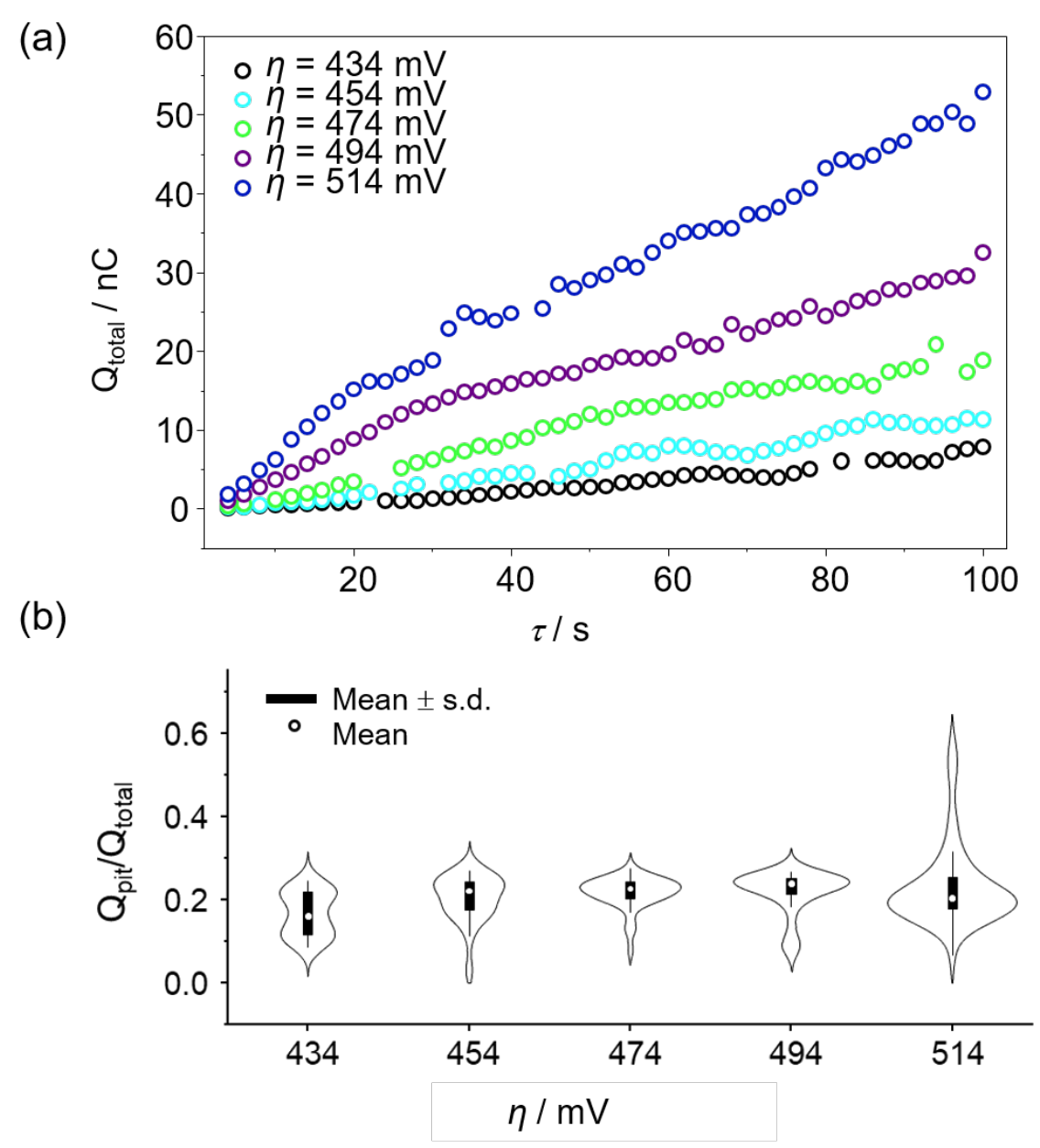

Figure S39. (a) Q_{total} as a function of τ for La-HEO-P-89 nm at different η ; (b) Violin plot of $Q_{\mathrm{pit}} / Q_{\mathrm{total}}$ as a function of η.

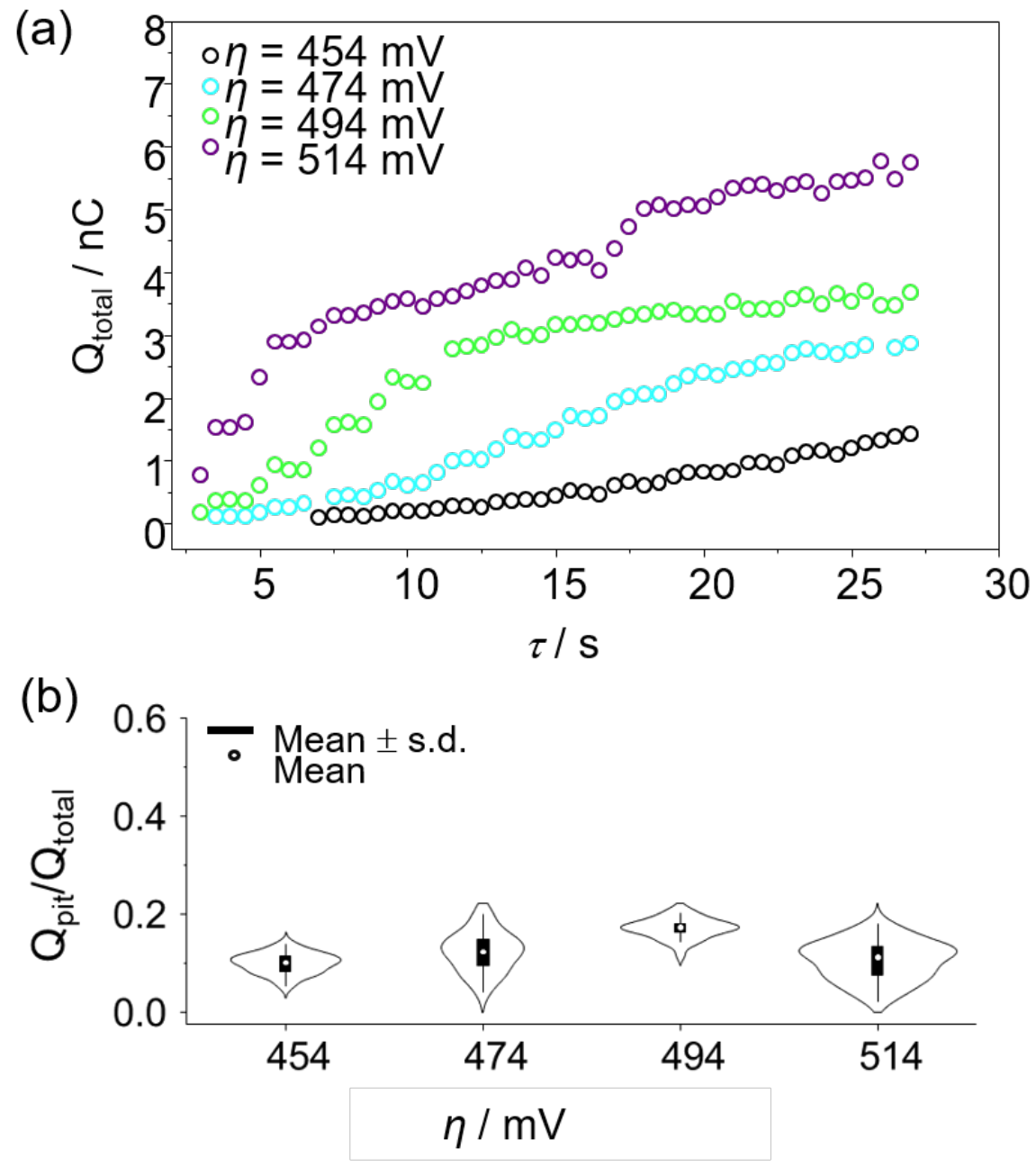

Figure S40. (a) Q_{total} as a function of τ for LaSr-HEO-P-29 nm at different η; (b) Violin plot of Q_{pit} / Q_{total} as a function of η.

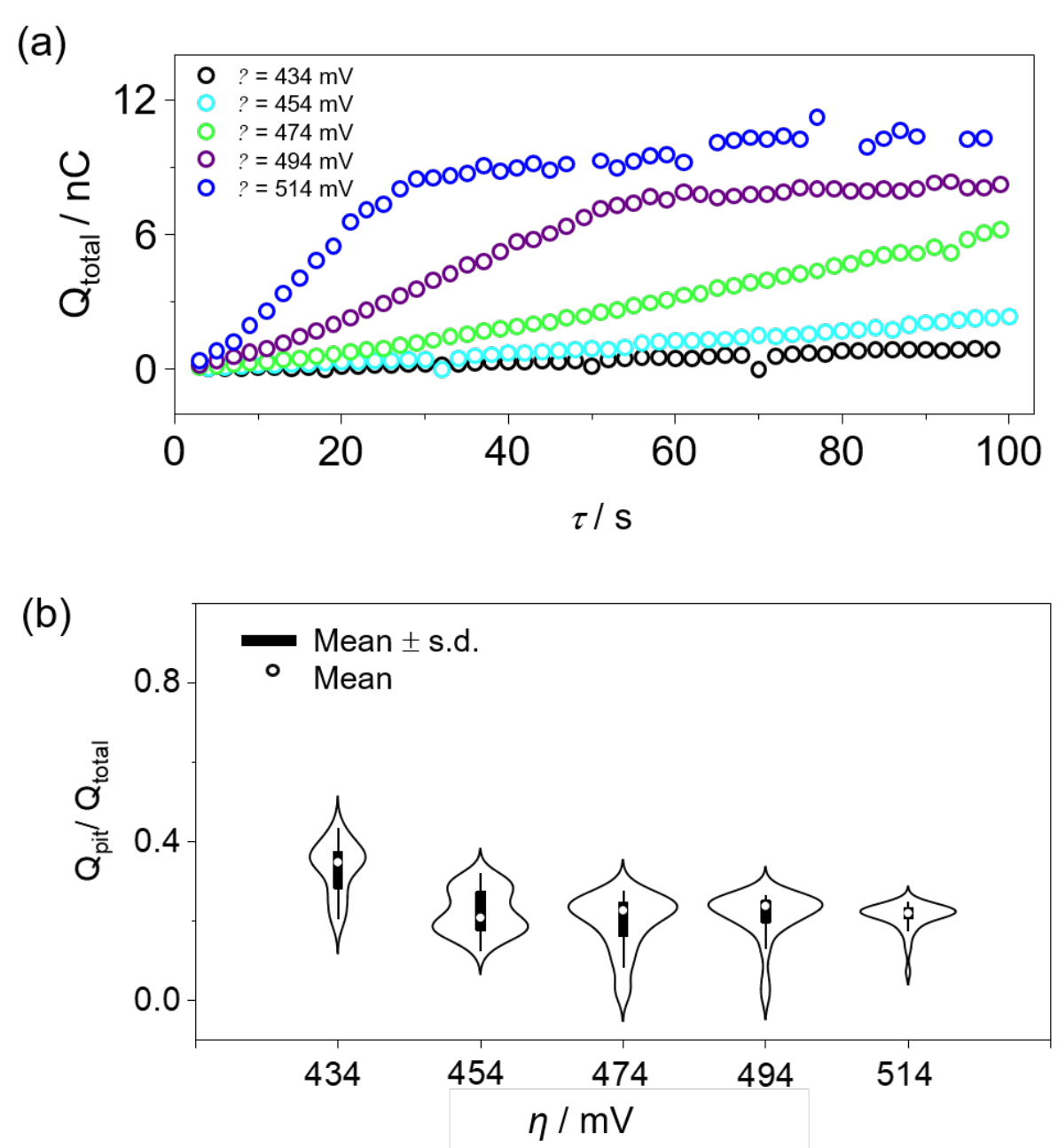

Figure S41. (a) Q_{total} as a function of τ for La-HEO-P-29 nm at different η; (b) Violin plot of Q_{pit} / Q_{total} as a function of at different η.

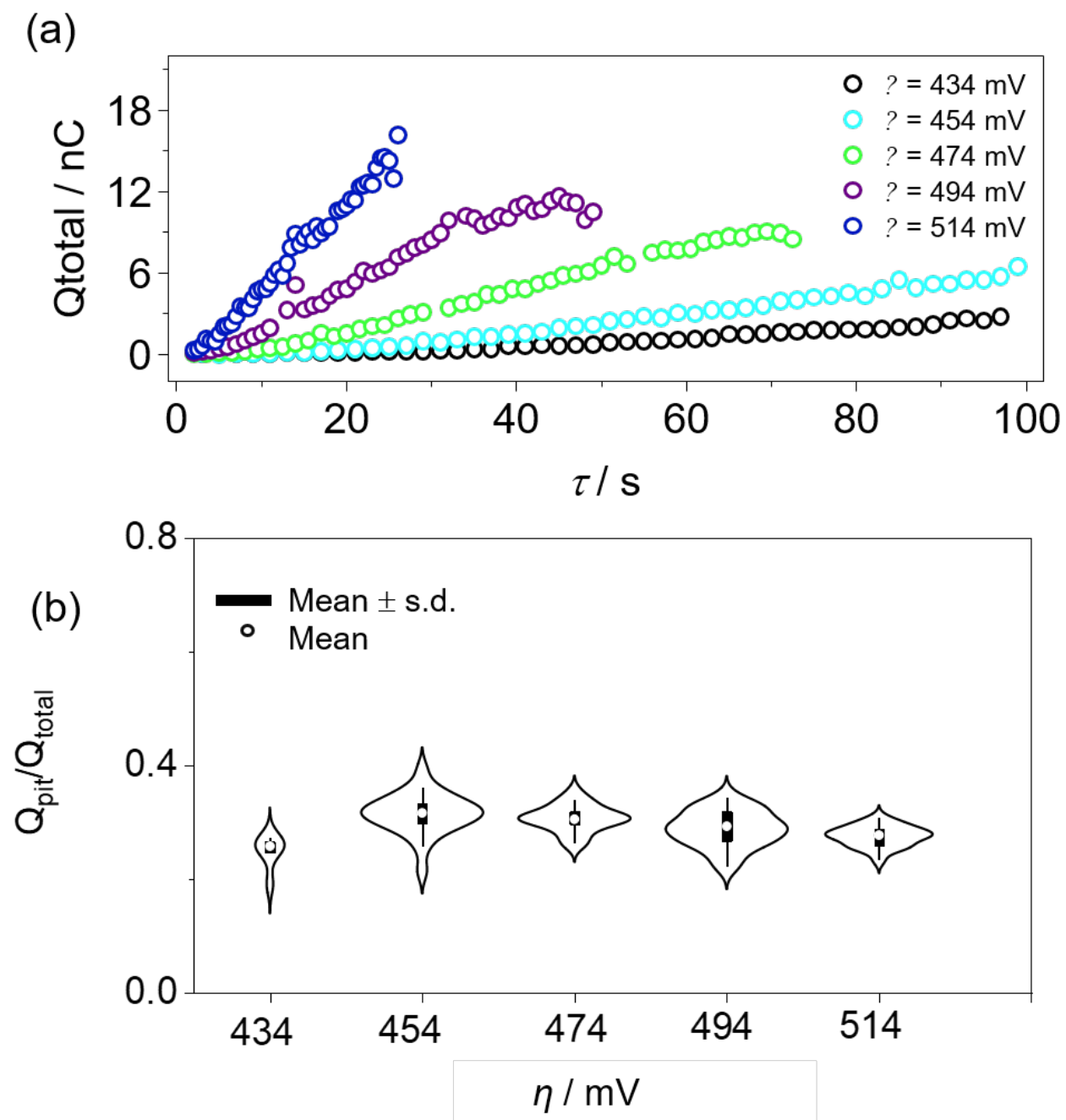

Figure S42. (a) Q_{total} as a function of τ for LaSr-HEO-P-100 nm at different η; (b) Violin plot of Q_{pit} / Q_{total} as a function of η.

Section S7. Experimental details of hop scanning with *i*–*t* on HEO-P electrode at different pH

Prior to the scan-hopping experiments, the QRCE was activated by anodization in saturated KCl solution and subsequently calibrated. 25 mM $HClO_4$ electrolyte was prepared with 100 mM $NaClO_4$ as the supporting electrolyte to maintain the same ionic strength and solution conductivity as in the previous experiments. The approach voltage and feedback threshold current were optimized to minimize false landing events while preventing premature perturbation of the pristine HEO-P surface. Lower threshold currents resulted in false landing detection, whereas higher threshold currents induced surface perturbation on timescales shorter than those relevant to the electrochemical measurements. The applied QRCE potential was then adjusted to maintain the same OER overpotential (η vs E^0_{OER}) used in the previous η dependent scan-hopping experiments, enabling direct comparison between the two datasets.

Scan-hopping experiments using a nanopipette filled with 25 mM $HClO_4$ were performed only on the La-HEO-P-29 nm and LaSr-HEO-P-100 nm, assuming that the pH dependence of the electrochemical response is independent of film thickness. For, La-HEO-P-29 nm, 6×6 scan hopping (49 spots) was done, starting $\tau = 3$s with $\Delta\tau = 3$s (at $\eta = 514$ mV). For, LaSr-HEO-P-100 nm, 6×6 scan hopping (49 spots), starting $\tau = 2$s with $\Delta\tau = 2$s (at $\eta = 494$ mV).

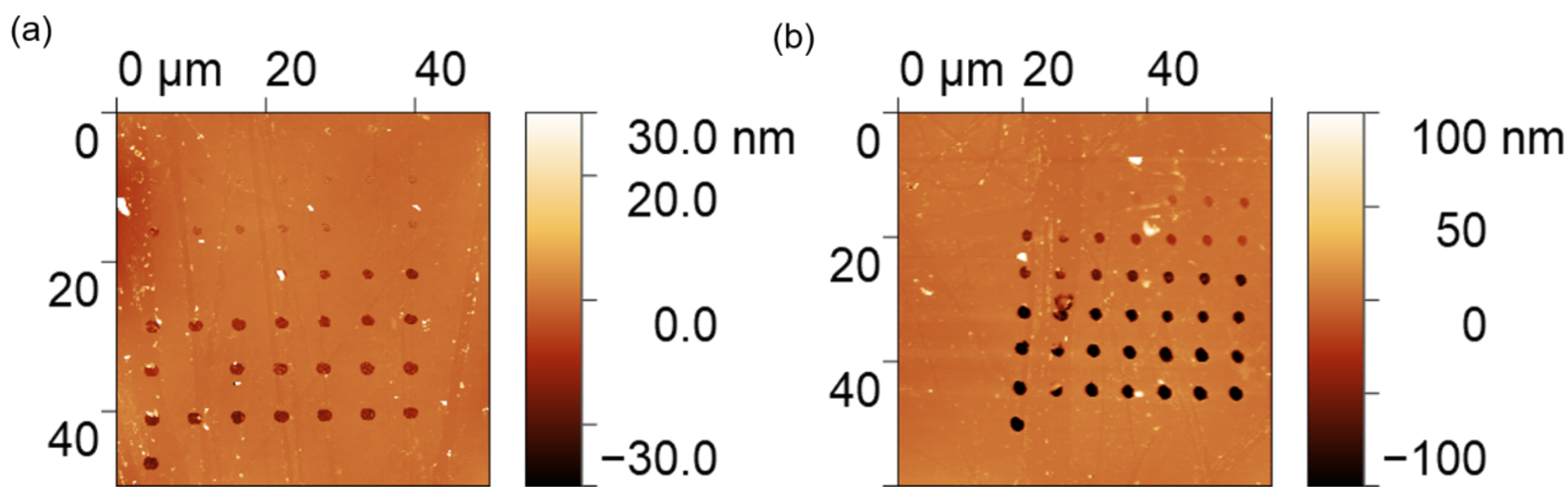

Figure S43. (a) AFM image of the area of La-HEO-P-29 nm; (b) LaSr-HEO-P-100 nm where *i–t* hopping experiments were performed using 25 mM $HClO_4$.

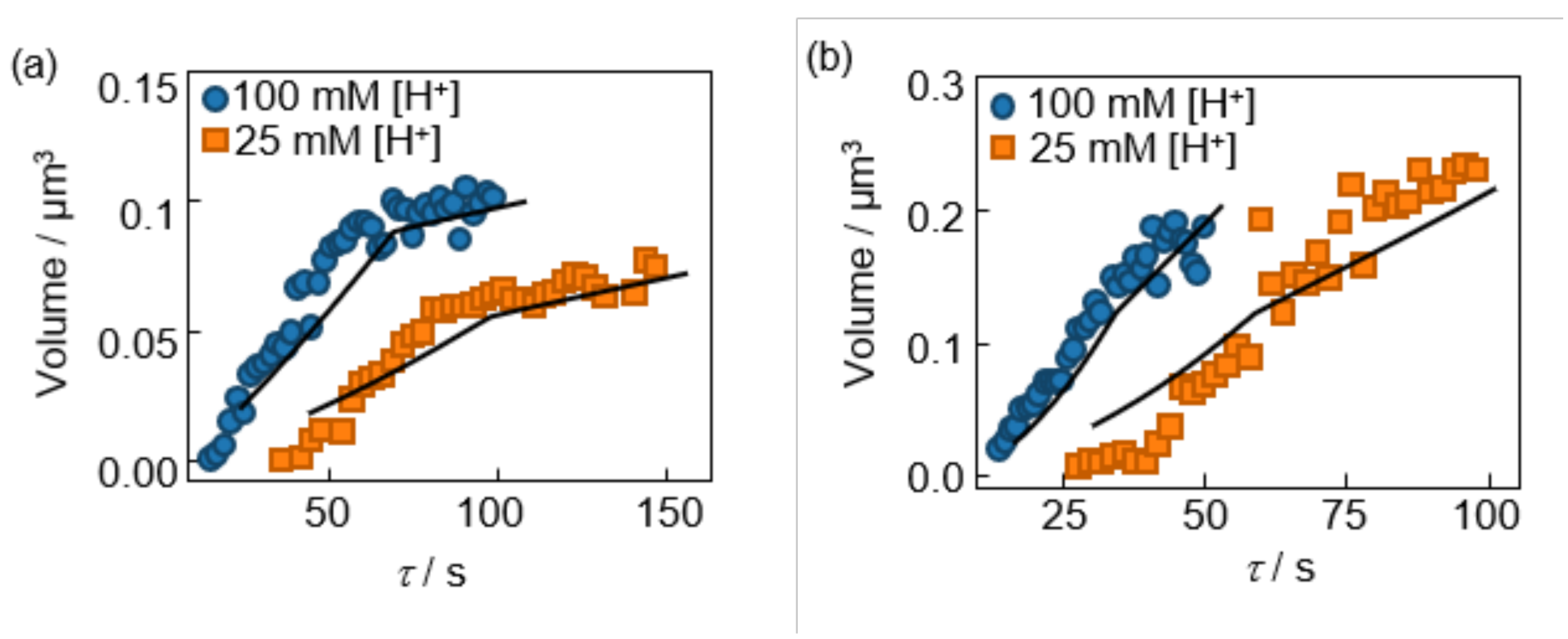

Figure S44. (a) Volume loss profile as a function τ at from the hop-scanning experiment for La-HEO-P-29 nm using 100 mM $HClO_4$ and 25 mM $HClO_4$ (100 mM $NaClO_4$ as supporting electrolyte) ; (b) for LaSr-HEO-P-100 nm. Black solid line represents the fit of the volume loss profile from the pit growth simulation.

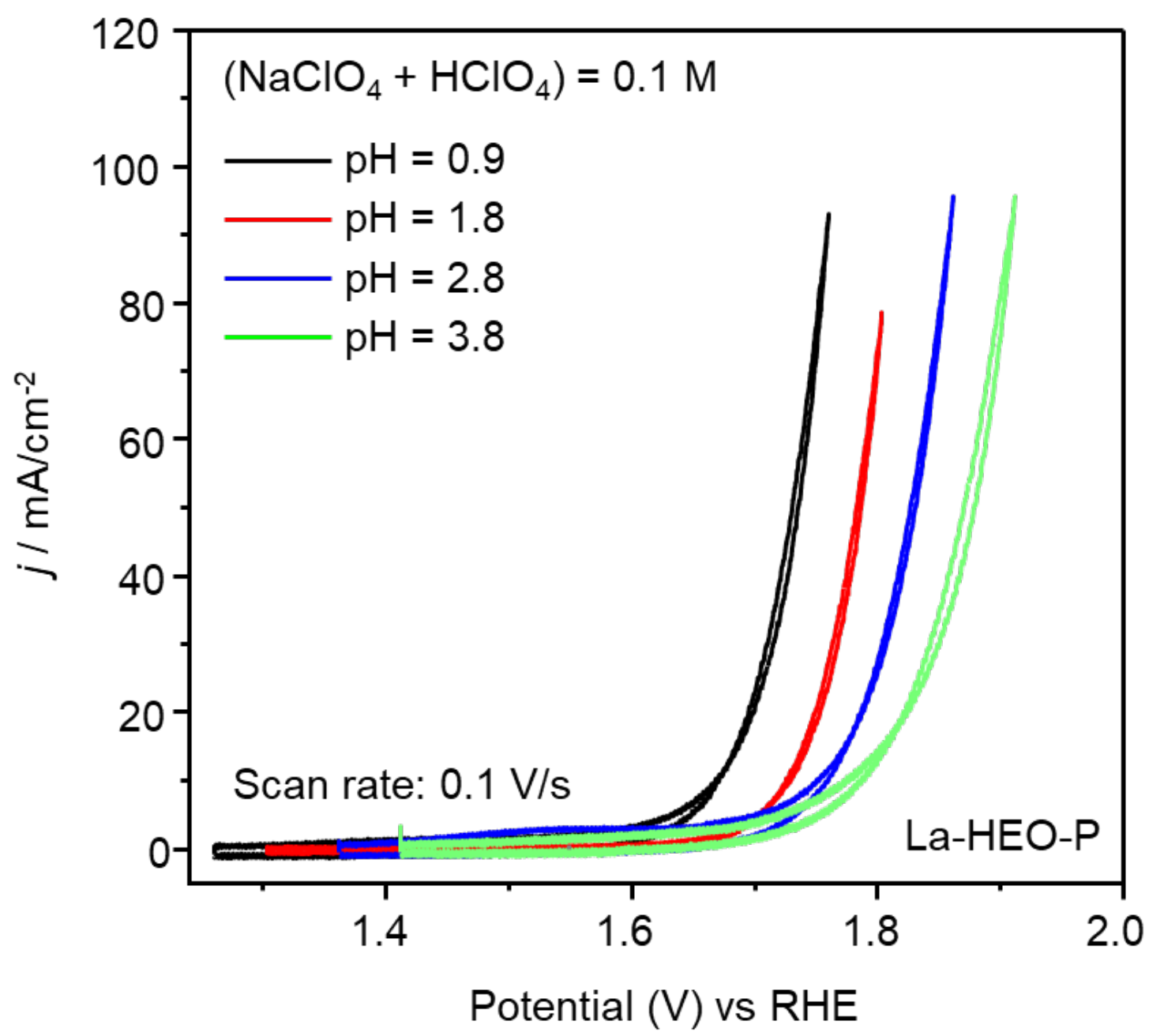

Figure S45. CV curves at different pH for La-HEO-P

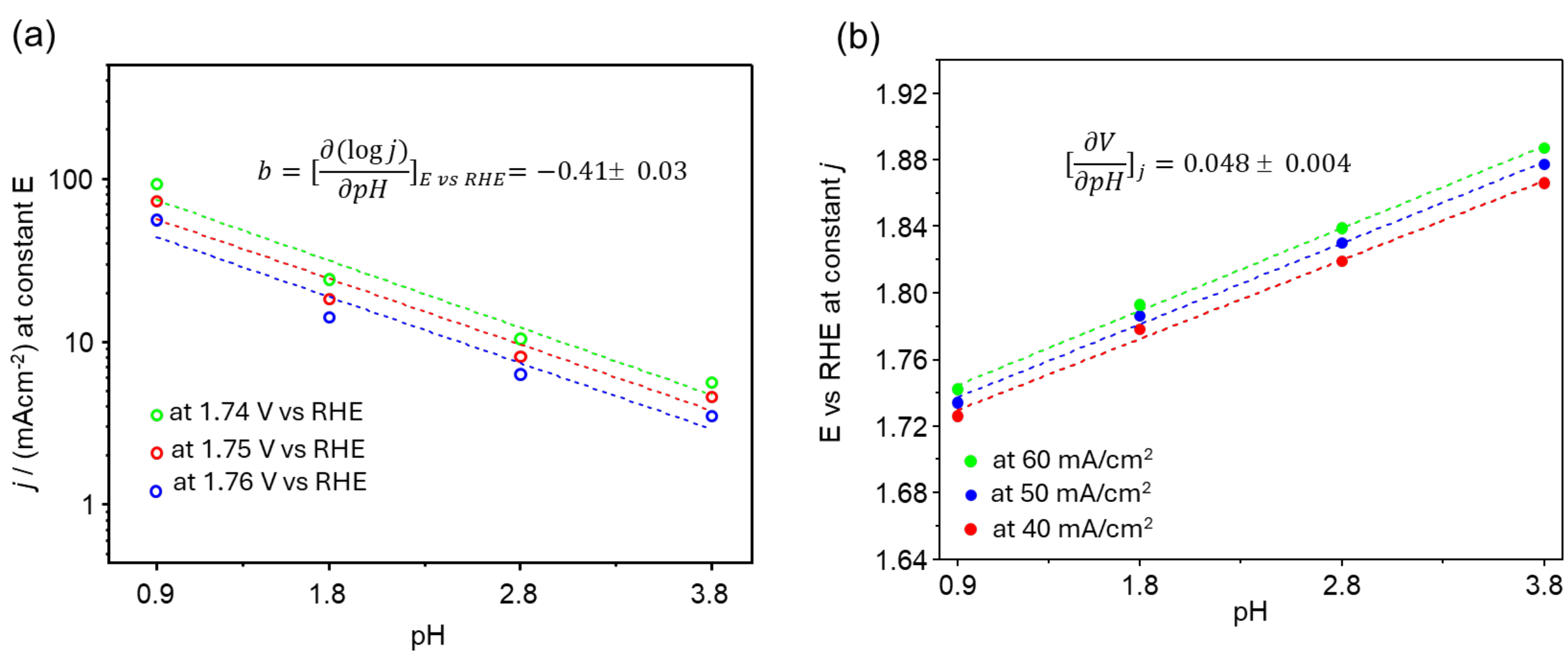

Figure S46. (a) Current density vs pH plot at different overpotential; (b) V vs pH plot for La-HEO-P at different current density.

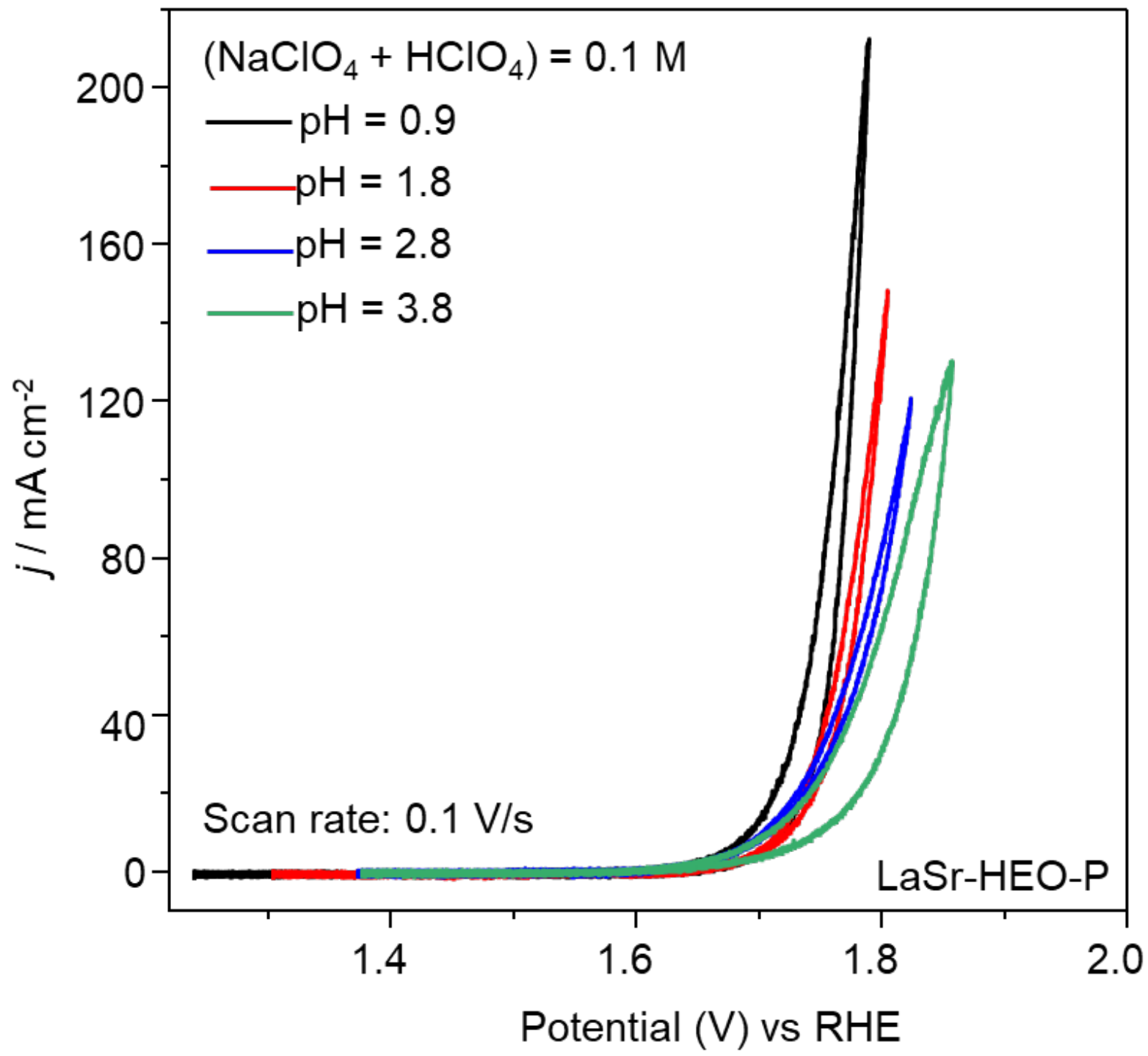

Figure S47. CV curves at different pH for LaSr-HEO-P .

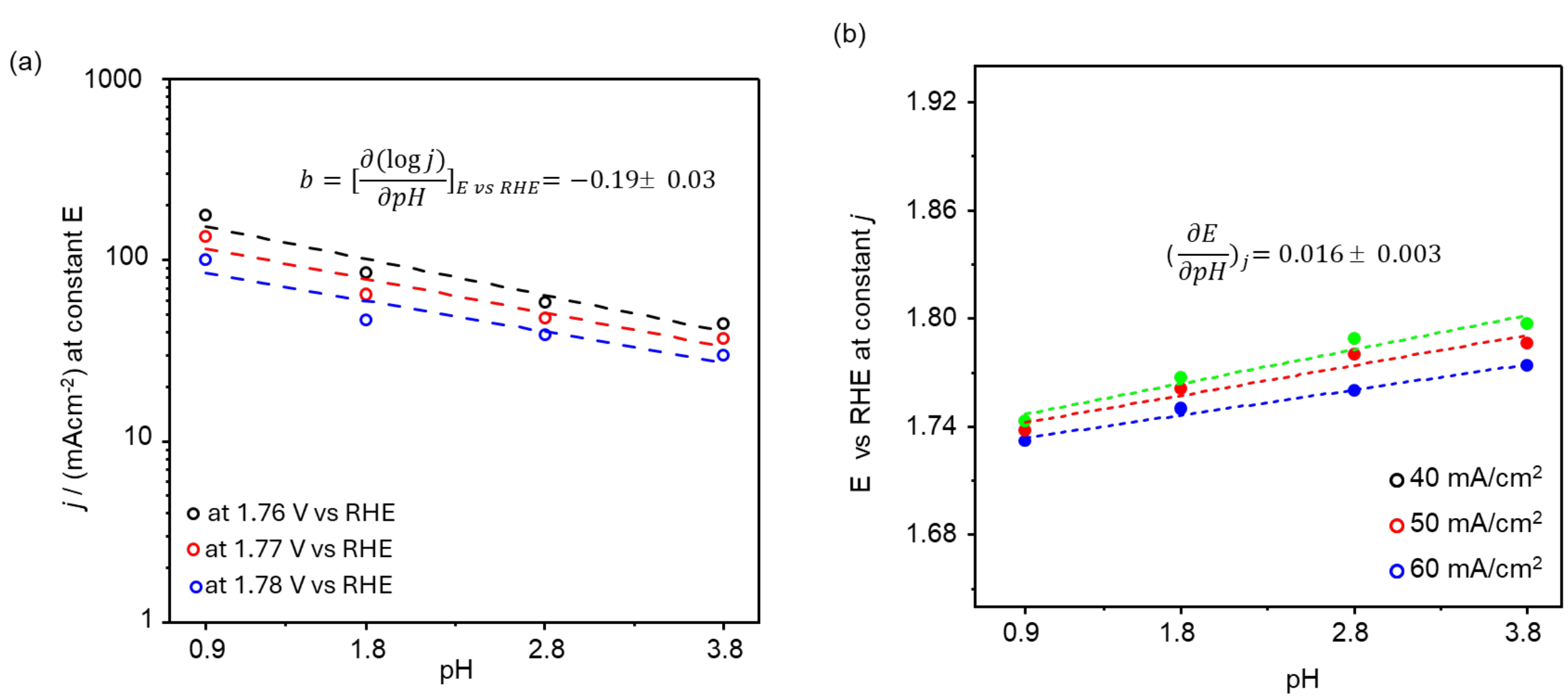

Figure S48. (a) Current density vs pH plot at different overpotential; (b) *E* vs pH plot for LaSr-HEO-P at different current density.

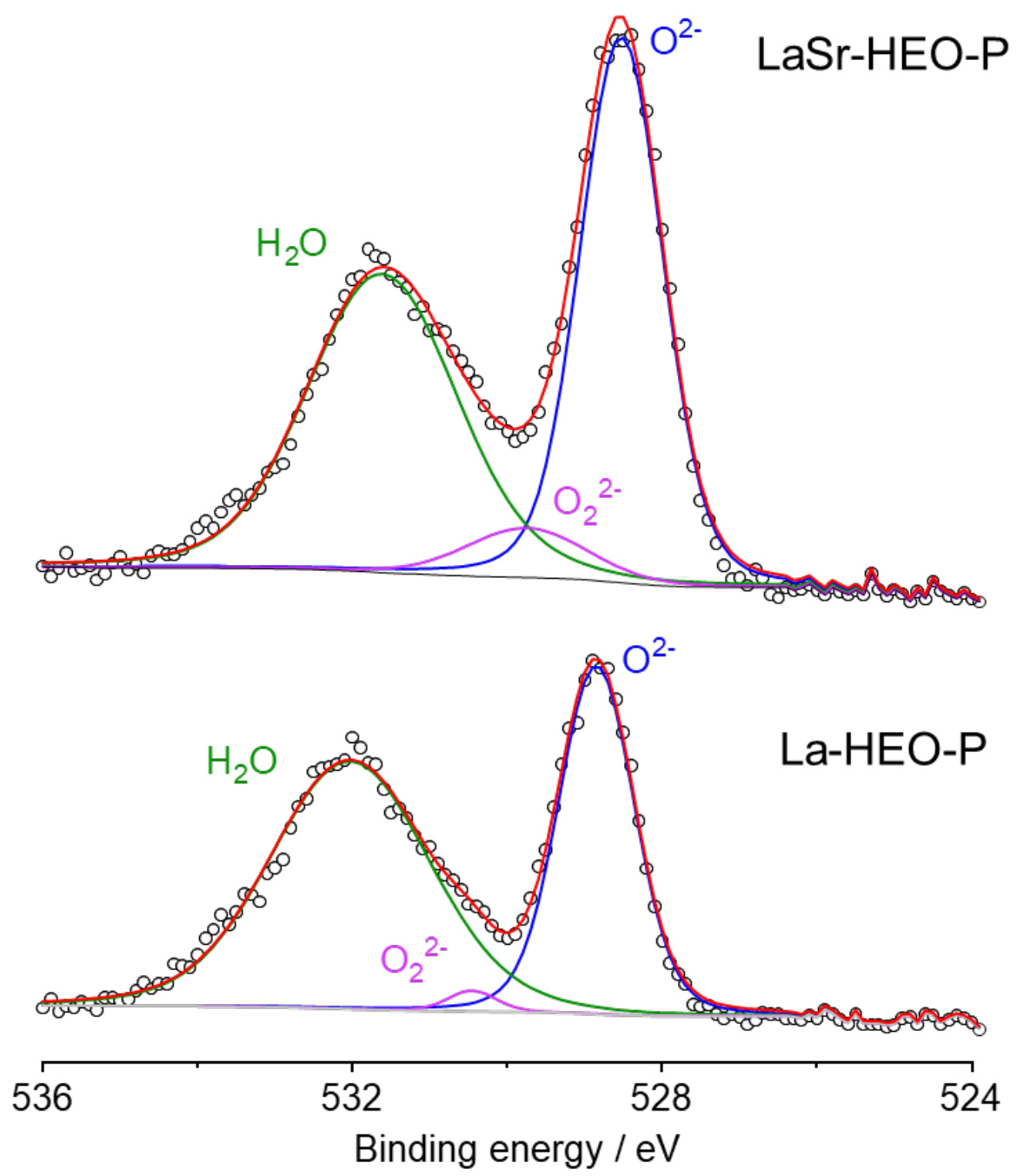

Figure S49. O1s XPS spectra for La-HEO-P and LaSr-HEO-P

Table S6. Binding energy (B.E.) of various components of O1s spectra of HEO-P electrodes .

O1s, LaSr-HEO-P	Description	B.E.
O^{2-}	Lattice oxygen	528.52
O_2^{2-}	Oxidized oxygen species	529.8
H_2O	Adsorbed water	531.7
O1s, La-HEO-P	Description	B.E.
O^{2-}	Lattice oxygen	528.9
O_2^{2-}	Oxidized oxygen species	530.5
H_2O	Adsorbed water	532.1

References

(1) Dąbrowa, J.; Zielińska, K.; Stępień, A.; Zajusz, M.; Nowakowska, M.; Moździerz, M.; Berent, K.; Szymczak, M.; Świerczek, K. Formation of Solid Solutions and Physicochemical Properties of the High-Entropy $Ln_{1-x}Sr_x(Co,Cr,Fe,Mn,Ni)O_{3-\delta}$ (Ln = La, Pr, Nd, Sm or Gd) Perovskites. *Materials* **2021**, *14* (18), 5264.

(2) Dąbrowa, J.; Olszewska, A.; Falkenstein, A.; Schwab, C.; Szymczak, M.; Zajusz, M.; Moździerz, M.; Mikuła, A.; Zielińska, K.; Berent, K.; Czeppe, T.; Martin, M.; Świerczek, K. An innovative approach to design SOFC air electrode materials: high entropy $La_{1-x}Sr_x(Co,Cr,Fe,Mn,Ni)O_{3-\delta}$ (x = 0, 0.1, 0.2, 0.3) perovskites synthesized by the sol–gel method. *J. Mater. Chem. A* **2020**, *8* (46), 24455-24468.

(3) Wang, L.; Koirala, K. P.; Wu, S.; Shi, J.; Kao, H.-M.; Ho, A.; Choi, M.-J.; Mantilla, A. B. C.; Qi, D.; Tadich, A.; Bowden, M. E.; Matthews, B. E.; Zhou, H.; Yang, Y.; Chang, C.-h.; Zhu, Z.; Wang, C.; Du, Y. Selective Oxidation and Cr Segregation in High-Entropy Oxide Thin Films. *Nano Lett.* **2025**, *25* (33), 12719-12727.

(4) Wetzel, A.; Morell, D.; von der Au, M.; Wittstock, G.; Ozcan, O.; Witt, J. Transpassive Metal Dissolution vs. Oxygen Evolution Reaction: Implication for Alloy Stability and Electrocatalysis. *Angew. Chem. Int. Ed.* **2024**, *63* (18), e202317058.

(5) Beverskog, B.; Puigdomenech, I. Revised pourbaix diagrams for chromium at 25–300 °C. *Corros. Sci.* **1997**, *39* (1), 43-57.

(6) Beverskog, B.; Puigdomenech, I. Revised Pourbaix diagrams for nickel at 25–300 °C. *Corros. Sci.* **1997**, *39* (5), 969-980.
